# Identification of genome size and heterozygosity in 510 Jujube (*Ziziphus jujuba* Mill.) germplasms based on deep resequencing

**DOI:** 10.3389/fpls.2026.1777223

**Published:** 2026-07-16

**Authors:** Wenshu Zhao, Yihan Yang, Hao Wu, Jiaqing Cai, Weiquan Zhou, Meng Yang, Mengjun Liu

**Affiliations:** 1 College of Horticulture/Research Center of Chinese Jujube, Hebei Agricultural University, Baoding, China; 2 College of Horticulture, Xinjiang Agricultural University, Urumqi, China

**Keywords:** classification, genome heterozygosity, genome size, jujube, k-mer analysis, resequencing depth

## Abstract

**Introduction:**

Genome size and heterozygosity represent the fundamental genetic attributes of a species, but their intraspecies diversity and evolution remain largely elusive.

**Methods:**

To address this, we developed a deep-resequencing-based approach for genome estimation by optimizing resequencing depth (30×) and kmer value (21) in Chinese jujube (*Ziziphus jujuba* Mill.) to achieve the best balance among accuracy, stability, and cost. Using this optimized pipeline, we estimated genome size and heterozygosity across a large panel of jujube accessions.

**Results:**

We estimated the genome size of 296 cultivated jujube genotypes from 293 to 409 Mb (mean 347 Mb, CV 5.94%) with heterozygosity of 1.30% to 2.01% (mean 1.72%, CV 8.21%); for 214 wild jujube, genome size varies between 311 and 391 Mb (mean 339 Mb, CV 3.40%), and heterozygosity 1.22% to 2.13% (mean 1.74%, CV 6.79%). Additionally, analyzing 798 jujube accessions with resequencing data in NCBI (using k-mer 21) revealed that low sequencing depths (10∼20×) of most accessions (98.61%) results in underestimated genome sizes (4.30–309 Mb) and overestimated heterozygosity (2.17-11.8%). Shannon-Wiener Index analysis for genome heterozygosity and size in jujube indicate a medium diversity level with cultivated jujube higher that wild one. Both genome size and heterozygosity show normal distribution, and there is a significant negative correlation between them.

**Discussion:**

During domestication from wild to cultivated jujube, average genome size increased by 8 Mb, while heterozygosity slightly decreased. A reference standard for genome size and heterozygosity is proposed and jujube is characterized as a small genome with mediumtohigh heterozygosity. This study establishes a powerful approach for estimating genome characteristics, enriched genomic resources for jujube, and provides insights for plant intraspecific genome diversity and evolution.

Genome size and heterozygosity are fundamental genetic characteristics of plants. Their accurate estimation is a critical prerequisite for high-quality genomic research, as it directly informs reliable genome assembly, evolutionary analysis, and molecular breeding applications. In genome assembly, both genome size and heterozygosity dictate sequencing data requirements and guide the selection of appropriate assembly strategies. Mochizuki et al. (2023) evaluated multiple genomic workflows-including the estimation of genomic features such as genome size and heterozygosity-across six genomes with varying heterozygosity levels. Their work demonstrated how accurate estimation of these parameters facilitates the choosing of suitable assembly methods and proposed specific guidelines for constructing haplotype-resolved assemblies based on heterozygosity degree. In evolutionary studies, genome size serves as a proxy for evolutionary divergence and a key genomic characteristic. Yan et al. (2016) estimated the genome sizes across 26 oat species, revealing correlations between genome size and evolutionary relationships. Moreover, genome size has been linked to adaptive traits in plants, underscoring its relevance for cultivation and stress resistance research (Kuo et al., 2021). In plant breeding, Chen et al. (2025) identified significant correlations between genomic heterozygosity and multiple agronomic traits in walnut, showing positive correlations with 13 traits and negative correlations with 8 traits, thereby offering practical insights for optimizing breeding strategies.

However, accurate estimation of genome size and heterozygosity remains challenging in practical research. In the genome regions with high intrinsic polymorphism, alignment difficulties often lead to systematic underestimation of true heterozygosity (Liao et al., 2024). For highly heterozygous species, secondary peaks in the k-mer frequency distribution further complicate the estimation process and compromise accuracy (Rong et al., 2024). Early methods for measuring genome size in plants primarily relied on Feulgen micro-densitometry (Greilhuber, 2008). While this approach allows analysis of small DNA quantities, provides stable results, and is applicable to diverse sample types (Biggiogera et al., 2024; Mello and Vidal, 2017), it also suffers from significant limitations, including hydrolytic instability, low staining efficiency, and susceptibility to interference from processing reagents (e.g., high concentrations or prolonged exposure to KMnO_4_) (Li et al., 2000). Subsequently, flow cytometry (FCM) gained popularity due to its rapid and straightforward protocol (Čertnerová, 2022). Nevertheless, the accuracy of FCM can be compromised by multiple factors in plant samples, such as: interference from secondary metabolites (e.g., phenolics and alkaloids that affect fluorescence detection), composition and concentration of extraction buffers, choice and dosage of fluorescent dyes, and tissue type-as DNA content varies with cell cycle stage across different tissues (Nix et al., 2024; Wang et al., 2015).

Currently, bioinformatics approaches based on genome resequencing and k-mer frequency distribution, owing to their efficiency and accuracy, have become the primary method for estimating genome size and heterozygosity in plants. The reliability of k-mer analysis depends on several factors, including sequencing data quality, the choosing of k-value, and sequencing depth. Contamination during sequencing-whether biological or technical-can introduce bias (Hesse, 2023). The selection of k-value significantly influences genome size and heterozygosity assessments: lower k-values improve the distinction between homozygous regions and sequencing errors, as well as between heterozygous and repetitive regions, but may reduce sequence specificity due to increased short k-mer redundancy. It is widely accepted that k-values below 17 generally fail to yield biologically meaningful information. Conversely, higher k-values reduce repetitive sequence interference and improve mapping specificity but are more susceptible to base-calling errors or insufficient coverage, leading to k-mer misrepresentation and compromised analytical accuracy (Moeckel et al., 2024; Roberts et al., 2025). Notably, k-values must be odd to avoid palindrome-related artifacts in k-mer identification and counting (Schmidt and Alanko, 2023). Furthermore, adequate sequencing depth is essential to capture a complete k-mer frequency profile for accurate estimation, whereas excessive depth incurs unnecessary costs.

Jujube (*Ziziphus jujuba* Mill.), the most economically and ecologically important economic tree in the Rhamnaceae family, exhibits remarkable environmental adaptability. Its fruits possess high nutritional and medicinal value. Originating in China’s Yellow River Basin, jujube is now cultivated across 48 countries in temperate and subtropical regions worldwide, where it plays an increasingly vital role in arid and saline-alkaline areas (Liu et al., 2020). Our previous research revealed that jujube genome is relatively small but very complex (Liu et al., 2014). Up to now, the detailed diversity of genome size and heterozygosity across different genotypes of jujube remain largely unclear. This study aims to establish a robust resequencing-based framework for accurate estimating genome size and heterozygosity in jujube. By applying this framework to a large collection of both cultivated and wild jujube, we seek to characterize their genomic diversity and evolutionary patterns. This work will enrich genomic resources for *Ziziphus* genus and provide valuable insights for genomic studies, breeding, and evolutionary research in jujube and horticultural plants.

## 1 Materials and methods

### 1.1 Plant materials and resequencing data sources

This study analyzed a total of 1,303 diploid *Ziziphus* accessions. The core sample set comprised 510 accessions: of these, 296 were cultivated jujube (*Ziziphus jujuba* Mill.) germplasms, sourced from the National Jujube Germplasm Repository (Taigu, Shanxi). This repository preserves representative landraces and bred cultivars covering major jujube-producing regions in China, including Shanxi, Shaanxi, Hebei, Shandong, and Xinjiang. The remaining 214 were wild jujube germplasms, obtained from the Germplasm Repository of Hebei Agricultural University. These materials were collected from natural wild populations in regions such as Hebei, Henan, and Shaanxi (original collection details are provided in **Supplementary Tables 2**, **3**). To broaden the analytical scope, we systematically retrieved and downloaded 793 published jujube resequencing datasets from the NCBI SRA database (details are provided in **Supplementary Table 1**). Sequencing was performed on the MGISEQ-2000 platform (BGI, Shenzhen, China), generating paired-end reads to support subsequent genomic analyses.

### 1.2 Estimation of genome size and heterozygosity

Genome size and heterozygosity were estimated using a k-mer-based analysis pipeline, with KMC (v3.0) employed for k-mer counting and GenomeScope 2.0 for model fitting. To ensure robust and cost-effective analyses, we first optimized the core parameters of this pipeline using deep-sequencing data from the ‘Dongzao’ jujube (at ~270× coverage). Specifically, to determine a stable k-mer size, we evaluated the impact of k-values ranging from 17 to 35 on genome size and heterozygosity estimates. Subsequently, to identify the optimal sequencing depth, we performed an in silico subsampling analysis using the determined optimal k-value (k=21), generating a gradient of sequencing depths from 10× to 270× for evaluation. The analytical procedure consisted of the following sequential steps:

- Data integrity verification: The integrity of the raw sequencing data files was verified using MD5 checksums to detect any potential corruption or tampering. For data obtained from the sequencing facility, the provided MD5 file was used directly. For publicly downloaded data, an MD5 file was first generated. The commands used were: A. md5sum *.gz > md5.txt. B. md5sum -c md5.txt.
- Quality control and adapter trimming: Raw sequencing reads often contain low-quality bases and adapter contaminants. Quality control was performed to remove these artifacts, ensuring the data met the standards required for robust downstream analysis. This was executed using the following example commands (illustrated for sample SRR10052895): A. fastp -i SRR10052895_1.fastq.gz B. -I SRR10052895_2.fastq.gz C. -o SRR10052895_1.cleandata.gz D. -O SRR10052895_2.cleandata.gz E. -l 36 -q 20 -n 6 -w 30 --compression=6.
- Mkdir -p tmp: This step aims to recursively create a temporary directory (tmp) to ensure the storage path for subsequent analysis files exists, thereby preventing program interruption due to missing directories. The specific code executed is as follows: A. mkdir -p tmp; B. echo SRR10052895_1.clean.gz >> SEQLIST; C. echo SRR10052895_2.clean.gz >> SEQLIST.
- K-mer frequency profiling: Genomic features were characterized by decomposing the quality-filtered reads into short subsequences of length k (k-mers). The frequency of each k-mer was counted to generate a k-mer spectrum. The analysis was performed with a k-mer size of 21 (k=21) using the command: A. kmc -k21 -t40 -m64 -ci1 -cs100000 @SEQLIST kmcdb tmp > SRR10052895.kmer.stat. Here, the -ci1 flag excludes unique k-mers (often indicative of sequencing errors), and -cs100000 sets an upper limit for k-mer coverage.
- Generation of K-mer Frequency Histogram: The raw k-mer counts were converted into a frequency distribution histogram, with k-mer coverage depth on the X-axis and the frequency of k-mers on the Y-axis. This histogram visually represents key genomic characteristics. It was generated using: A. kmc_tools transform kmcdb histogram kmcdb_k21.hist -cx10000.
- Model fitting and parameter estimation: Finally, the k-mer frequency histogram was analyzed using GenomeScope 2.0 to fit a model and estimate key genomic parameters. This included genome size (haploid length), heterozygosity rate, and repeat sequence content. The following command was used: A. Rscript genomescope2.0-master/genomescope.R -i kmcdb_k21.hist -o SRR10052895.GS -k 21 -p.

### 1.3 Statistical analysis

All statistical analyses were performed using SPSS software. Since the data for genome size and heterozygosity violated the normality assumption (Shapiro-Wilk test, p < 0.05), non-parametric tests were employed: differences between groups were assessed using the Mann-Whitney U test, and correlations were analyzed using Spearman’s rank correlation coefficient (ρ). The p-values derived from multiple correlation analyses were adjusted using the Bonferroni method (adjusted significance threshold α’ = 0.01). To specifically investigate the potential associations between genomic features and agronomic traits, Spearman’s rank correlation analysis was conducted on genomic heterozygosity, genome size, and mean single fruit weight across 20 jujube cultivars. The Shapiro-Wilk test confirmed that the fruit weight data also significantly deviated from a normal distribution (W = 0.839, P = 0.004), further justifying the use of this non-parametric approach.

## 2 Results

### 2.1 Optimization of resequencing-based estimation of genome size and heterozygosity

#### 2.1.1 Determination of the optimal k-mer value

To establish a robust protocol for genome characterization in jujube, we systematically evaluated the influence of different k-mer values (ranging from 17 to 35) on the estimation of genome size and heterozygosity. This evaluation was performed using 30×whole-genome sequencing data from three jujube accessions: ‘Dongzao’ (*Ziziphus jujuba* Mill. ‘Dongzao’), sour jujube, and jun-jujube.

**Figure 1:**
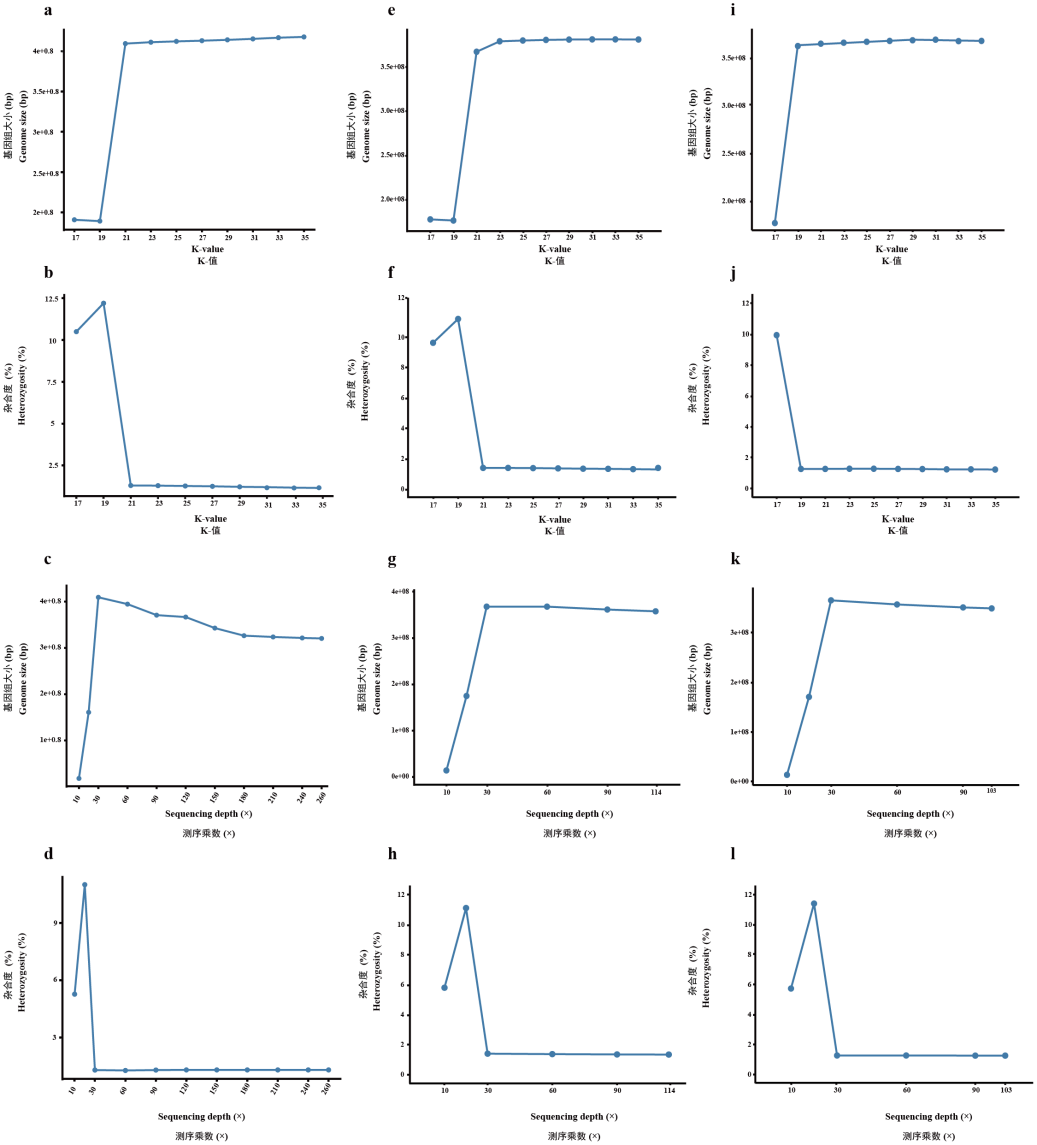
Effects of K-mer value and sequencing depth on the estimation of genomic. **(a-d)** Dongzao; **(e-h)** Sour jujube; **(i-l)** Junzao. **(a, e, i)** Relationship between K-mer value and genome size; **(b, f, j)** Relationship between K-mer value and genome heterozygosity; **(c, g, k)** Relationship between sequencing depth and genome size; **(d, h, l)** Relationship between sequencing depth and genome heterozygosity.

As illustrated in **Figure 1a**, the estimated genome size showed an increasing trend with the increase of k-value but stabilized notably once the k-value reached 21 (**Figure 1a**). Concurrently, the estimated genome heterozygosity initially increased and then decreased with the increase of k-value, also plateauing after k = 21 (**Figure 1b**).To validate the universality of this parameter, we further performed tests on sour jujube (wild type) and jun-jujube (cultivated type). The results showed that, with the sequencing depth fixed at 30×, as the k−value increased, the estimated heterozygosity of both varieties dropped significantly and stabilized at relatively low levels (~1.39% for sour jujube and ~1.26% for jun−jujube), while their estimated genome sizes also reached a stable plateau (**Figures 1e, f, i, j**). This confirms that k = 21 can effectively distinguish heterozygous signals from repetitive sequences in jujube materials with different genetic backgrounds, thereby ensuring the robustness of the analysis.

Based on these results, the k-value of 21 was identified as the optimal parameter for jujube genome analysis. This value strikes a balance between genome coverage and mapping specificity, ensuring reliable estimation of genome size and heterozygosity while maintaining computational efficiency - a crucial factor for large-scale genomic studies.

#### 2.1.2 Determination of the optimal genome resequencing depth

To determine the cost-effective sequencing depth for reliable genome characterization in jujube, we evaluated the impact of a wide range of sequencing depths (10× to 270×) for ‘Dongzao’, (10× to 114×) for sour jujube, and (10× to 103×) for jun-jujube—on genome size and heterozygosity estimation using a k-mer size of 21.

As shown in **Figure 1c**, the estimated genome size initially increased, then decreased, and gradually stabilized with the increasing of sequencing depth. At 30× depth, the estimated genome size was 409 Mb, which aligns closely with the established reference genome size of approximately 411.6 Mb (Yang et al., 2023). The genome size of ‘Dongzao’ was determined to be approximately 444 Mb using flow cytometry, with *Pyrus betulifolia* (532.7 Mb) serving as the internal reference standard. Although the physical genome size measured by flow cytometry is typically slightly larger than the total length of the assembled sequences—as it accounts for all repetitive regions that are challenging to assemble—the close agreement between these two results effectively achieves methodological cross-validation and confirms the accuracy of the findings. Beyond 30×, the estimated genome size changed slightly. A similar trend was observed for heterozygosity (**Figure 1d**): the estimated value rose initially, then declined rapidly, and kept stable after sequencing depth reached 30×. At 30× depth, the genome-wide heterozygosity was approximately 1.31%. Similarly, with k fixed at 21 for sour jujube and jun-jujube, as sequencing depth increased, both heterozygosity and genome size estimates stabilized when the depth reached 30×, and further increases in depth yielded no significant improvement (**Figures 1g, h, k, l**). Further increases in depth beyond this point yielded no significant improvement in estimation accuracy but substantially raised sequencing costs.

Therefore, a sequencing depth of 30× was identified as the optimal one for jujube genome analysis. This depth provides a balanced approach, ensuring accurate and stable estimations of both genome size and heterozygosity while maintaining cost efficiency-critical advantage for large-scale genomic studies in plants.

#### 2.1.3 Application of the optimized parameters to public genome resequencing data of jujube

To validate the above optimized parameters and assess the reliability of public genomic resources in genomic study, we analyzed the genome resequencing data from 793 jujube accessions obtained from the NCBI database (K-mer = 21). The K-mer frequency distributions were generated using KMC and profiled with GenomeScope 2.0 (**Supplementary Table 1**).

Our analysis revealed a pronounced prevalence of low sequencing coverage in the publicly available datasets. Only 11 accessions (1.39%) were sequenced to a depth greater than 30×. For these accessions, the estimated genome sizes ranged from 324 to 399 Mb (mean: 363 Mb), with heterozygosity rates between 1.20% and 1.68% (mean: 1.39%). These values are well consistent with those obtained from our deep resequencing (30×) of 510 jujube accessions, confirming the reliability of the estimates derived from sufficient sequencing depth.

In stark contrast, the vast majority of jujube accessions from NCBI database (n = 782, 98.61%) were sequenced at suboptimal depths below 30×, with 771 accessions having only 10-20× coverage. This insufficient depth led to severe inaccuracies: genome sizes were substantially underestimated (4.30–309 Mb), while heterozygosity was markedly inflated (2.17%-11.8%). These estimates deviate significantly from the benchmarks established using high-quality genome assemblies, rendering them unreliable for downstream analyses.

Collectively, the above comparative results indicated that a minimum sequencing depth of 30× is critical for obtaining accurate and biologically meaningful estimates of genome size and heterozygosity in jujube.

### 2.2 Genome size and heterozygosity estimation across 296 jujube cultivars using 30× resequencing data

Using the optimized bioinformatics workflow (KMC + GenomeScope 2.0, k-mer = 21, sequencing depth = 30×), we characterized the genome size and heterozygosity of 296 cultivated jujube accessions. Both traits exhibited an approximately normal distribution across the population.

**Figure 2:**
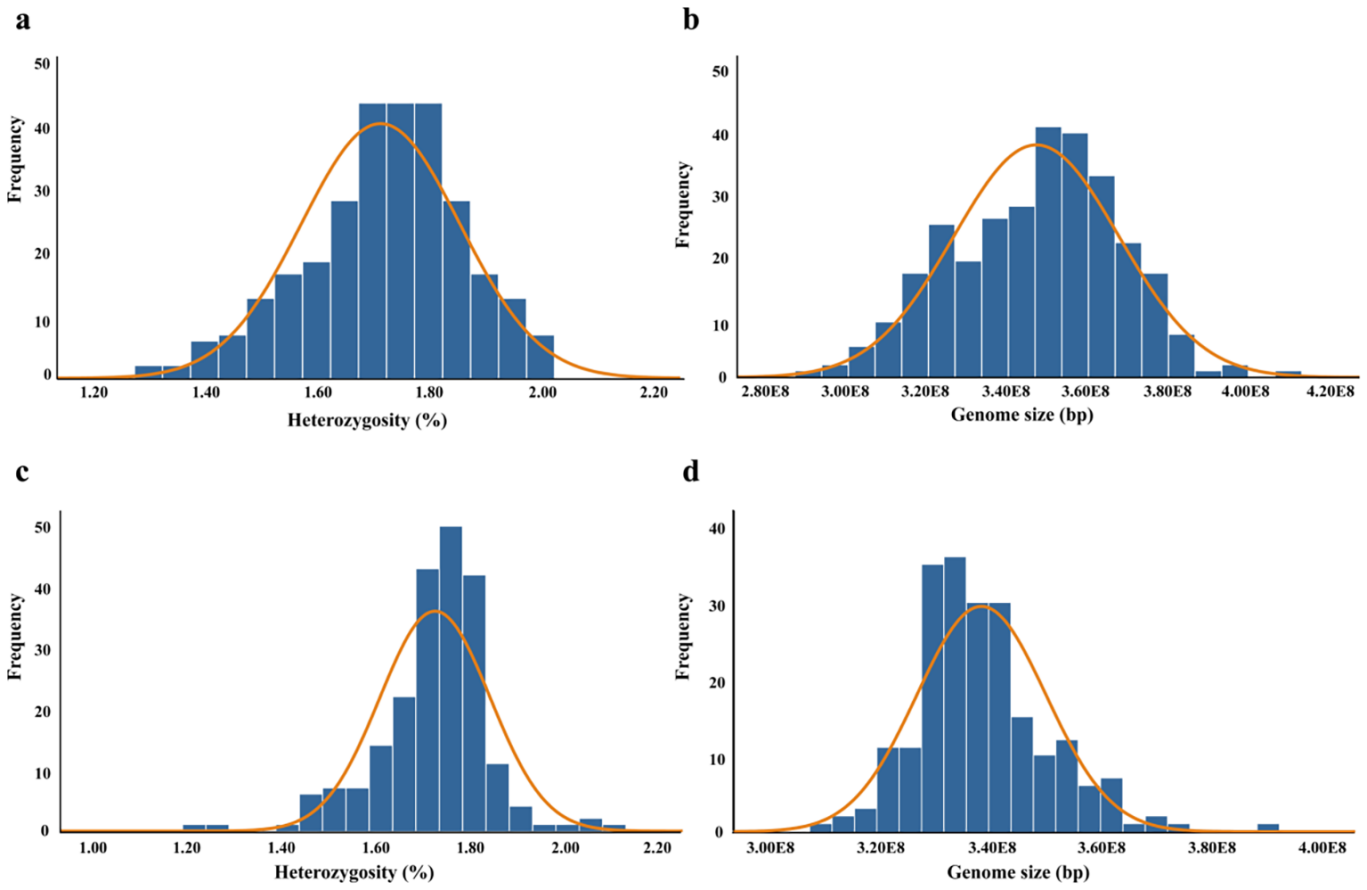
Frequency distribution of genomic characteristics in cultivated and wild jujube. **(a, b)** Frequency distribution histogram of genome heterozygosity **(a)** and genome size **(b)** in cultivated jujube; **(c, d)** Frequency distribution histogram of genome heterozygosity **(c)** and genome size **(d)** in wild jujube.

As shown in **Figure 2**; **Supplementary Table 2**, the genome heterozygosity ranged from 1.30% to 2.01%, with a mean of 1.72% (coefficient of variation, CV = 8.21%). Genome size varied from 293 Mb to 409 Mb, with a mean of 347 Mb (CV = 5.94%). ‘Neihuangpingguozao’ showed the lowest heterozygosity (1.31%) and a genome size (378 Mb) above-average. In contrast, ‘Dunhuangdazao’ displayed the highest heterozygosity (2.01%) with a genome size (345 Mb) near the mean. ‘Changxindianbaizao’ had the smallest genome (293 Mb) and a heterozygosity (1.84%) above-average, while ‘Dongzao’ possessed the largest genome (409 Mb) coupled with a heterozygosity (1.38%) below-average.

Analysis of the genetic diversity in the studied cultivated jujube cultivars revealed Shannon-Wiener index (H’) values of 2.04 for genome heterozygosity and 2.08 for genome size, demonstrating a medium level of diversity.

### 2.3 Genome size and heterozygosity estimation across 214 wild jujube genotypes using 30× resequencing data

We applied the same optimized bioinformatics workflow (KMC + GenomeScope 2.0, k-mer = 21, sequencing depth = 30×) to analyze the genomic characteristics of 214 wild jujube accessions, the wild progenitor of cultivated jujube. Both genome size and heterozygosity were found to be normally distributed across the wild population (**Figure 2**).

As shown in **Figure 2**; **Supplementary Table 3**, the genome-wide heterozygosity of wild jujube genotypes ranged from 1.22% to 2.13%, with a mean of 1.74% (CV = 6.79%). Genome size varied from 311 Mb to 391 Mb, with a mean of 339 Mb (CV = 3.40%). Notable variations were observed among genotypes. ‘LW7-20’ exhibited the lowest heterozygosity (1.22%) and an above-average genome size (372 Mb). In contrast, ‘D1Y3’ showed the highest heterozygosity (2.13%) with a genome size close to the mean (321 Mb). The smallest genome (311 Mb) was found in ‘LW4’, which also displayed above-average heterozygosity (1.91%), whereas the largest genome (391 Mb) was identified in ‘LW7-19’, accompanied by below-average heterozygosity (1.45%).

Analysis of the genetic diversity in the studied wild jujube genotypes revealed Shannon-Wiener index (H’) values of 1.88 for genome heterozygosity and 1.98 for genome size, both lower than those in cultivated jujube, also indicating a medium level of diversity.

### 2.4 Comparison of genome characteristics between cultivated jujube and wild jujube

To elucidate genomic changes associated with jujube domestication, we compared the genome size and heterozygosity between cultivated jujube and its wild ancestor, wild jujube, using a k-mer-based approach (KMC + GenomeScope 2.0, k = 21). Based on 30× whole-genome resequencing data, we systematically compared the 296 cultivated jujube varieties and 214 wild jujube accessions.

**Figure 3:**
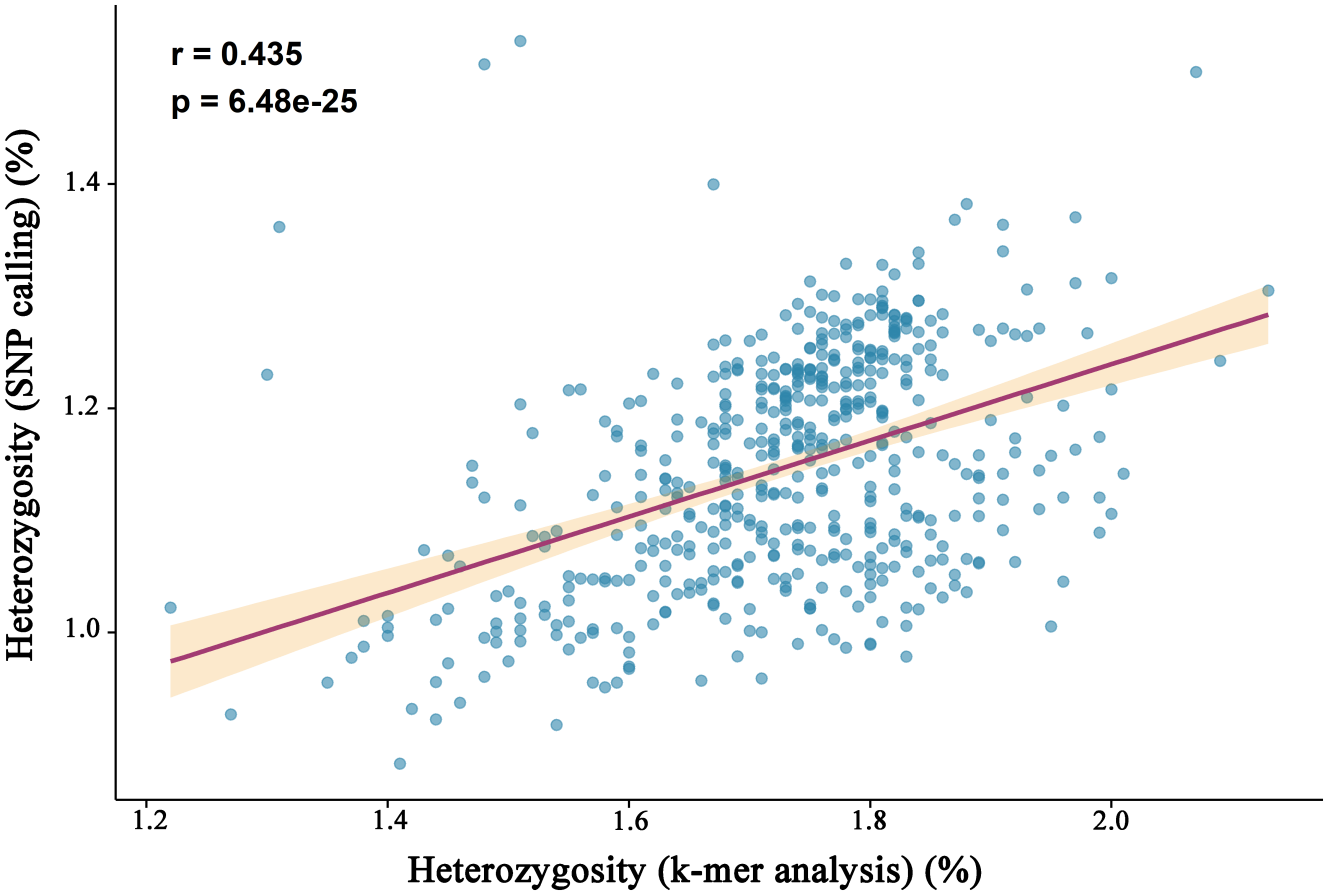
Cross-validation of heterozygosity estimation methods.

To independently validate the heterozygosity results obtained from k-mer analysis, we compared them with heterozygosity estimates derived from SNP calling. Pearson correlation analysis performed on 508 accessions revealed a significant positive correlation between the two metrics (r = 0.435, p < 0.001; **Figure 3**). This confirms that, although the absolute numerical scales of the two methods differ due to their distinct principles, our k-mer-based approach is reliable for assessing relative differences in heterozygosity among samples.We calculated Model Fit (%), number of convergence iterations, convergence tolerance, and residual standard error (RSE) to assess the goodness of fit and numerical stability of the model (detailed statistics are provided in **Supplementary Table 6**). The results show that all samples achieved stable convergence, with a final unified convergence tolerance of 1.49 × 10^-^^8^and a relatively low number of iterations (typically 6-22), indicating a stable and reliable model optimization process.

**Figure 4:**
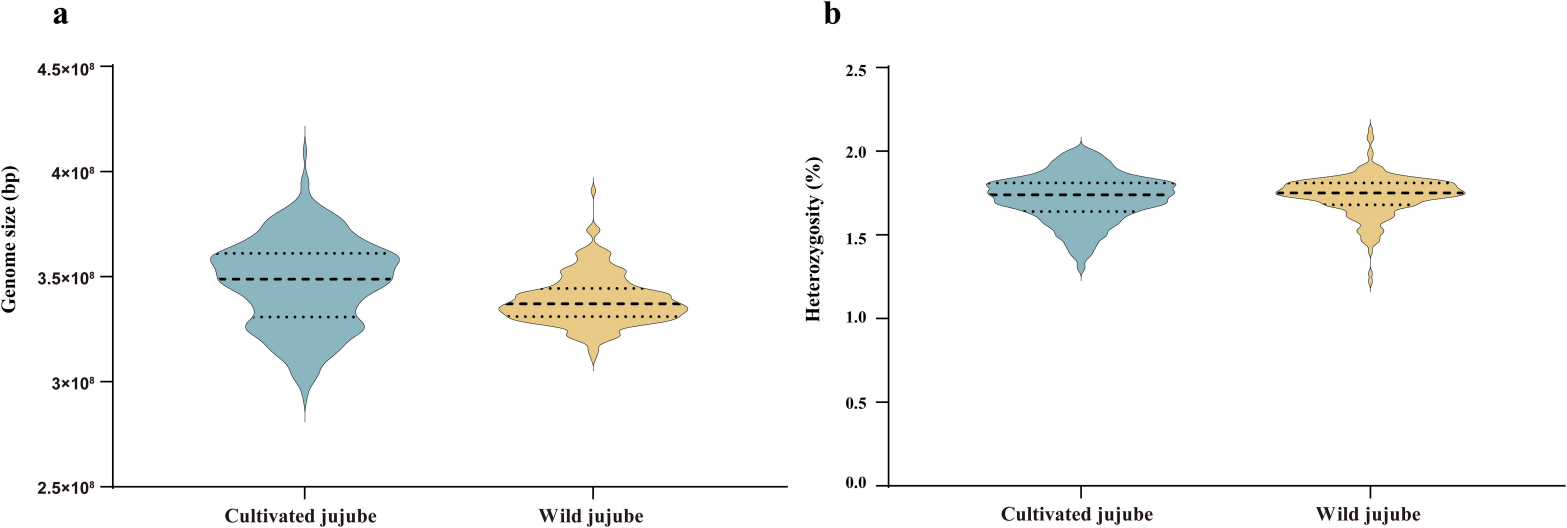
The genome size **(a)** and heterogeneity **(b)** distribution of the number of genotypes in cultivated jujube and wild jujube.

Comparative analysis revealed different genomic patterns between the cultivated jujube and wild jujube group. The 296 cultivated jujube accessions exhibited genome sizes ranging from 293 to 409 Mb (mean: 347 Mb; CV: 5.94%) and heterozygosity from 1.30% to 2.01% (mean: 1.72%; CV: 8.21%). In contrast, the 214 wild jujube accessions showed a narrower genome size range (311–391 Mb) with a lower mean (339 Mb) and substantially reduced variability (CV: 3.40%). Wild jujube also displayed a marginally higher mean heterozygosity (1.74%; range: 1.22-2.13%) but with lower variation (CV: 6.79%) compared to cultivated jujube (**Figure 4**). In case of genome dicersity, the Shannon-Wiener Indices of cultivated jujube (2.04 for genome heterozygosity and 2.08 for genome size) are bigger than those of wild jujube (1.88 and 1.98).

To evaluate the statistical significance of the observed inter-group differences and explore the intrinsic relationships between genomic features, further analyses were conducted. The Shapiro-Wilk test indicated that neither genome size nor heterozygosity followed a normal distribution in either group (all p < 0.05), leading to the use of the non-parametric Mann-Whitney U test for comparisons. The results revealed a highly significant difference in genome size between cultivated and wild jujube (U = 22494.0, p < 0.001), with the median genome size of cultivated jujube (3.49 × 10^8^) being significantly larger than that of wild jujube (3.37 × 10^8^), and the Common Language Effect Size (CL = 0.355) confirming the practical relevance of this difference. In contrast, the median heterozygosity values were similar between the two groups (cultivated: 1.74; wild: 1.75) and showed no statistically significant difference (U = 29806.5, p = 0.256, CL = 0.471). Furthermore, Spearman’s rank correlation analysis demonstrated a significant negative correlation between genome size and heterozygosity across all samples (ρ = -0.436, p < 0.001). This negative correlation was also significant within each group, being strongest in the wild jujube group (ρ = -0.527, p < 0.001) and slightly weaker but still significant in the cultivated jujube group (ρ = -0.473, p < 0.001). All p-values for correlation analyses remained significant after Bonferroni correction for multiple testing (adjusted threshold α’ = 0.01), underscoring the robustness of these findings.

Overall, wild jujube is characterized by a smaller average genome size, slightly higher genome heterozygosity and lower genome diversity relative to its cultivated counterpart. These differential patterns may reflect the combined effects of genomic structural variations, genetic drift, and sustained artificial selection during jujube domestication. Our findings provide foundational insights into the genomic consequences of domestication and will facilitate the identification of key genetic loci for future breeding and germplasm utilization in jujube.

### 2.5 Association of genomic features with fruit traits and its implications

**Figure 5:**
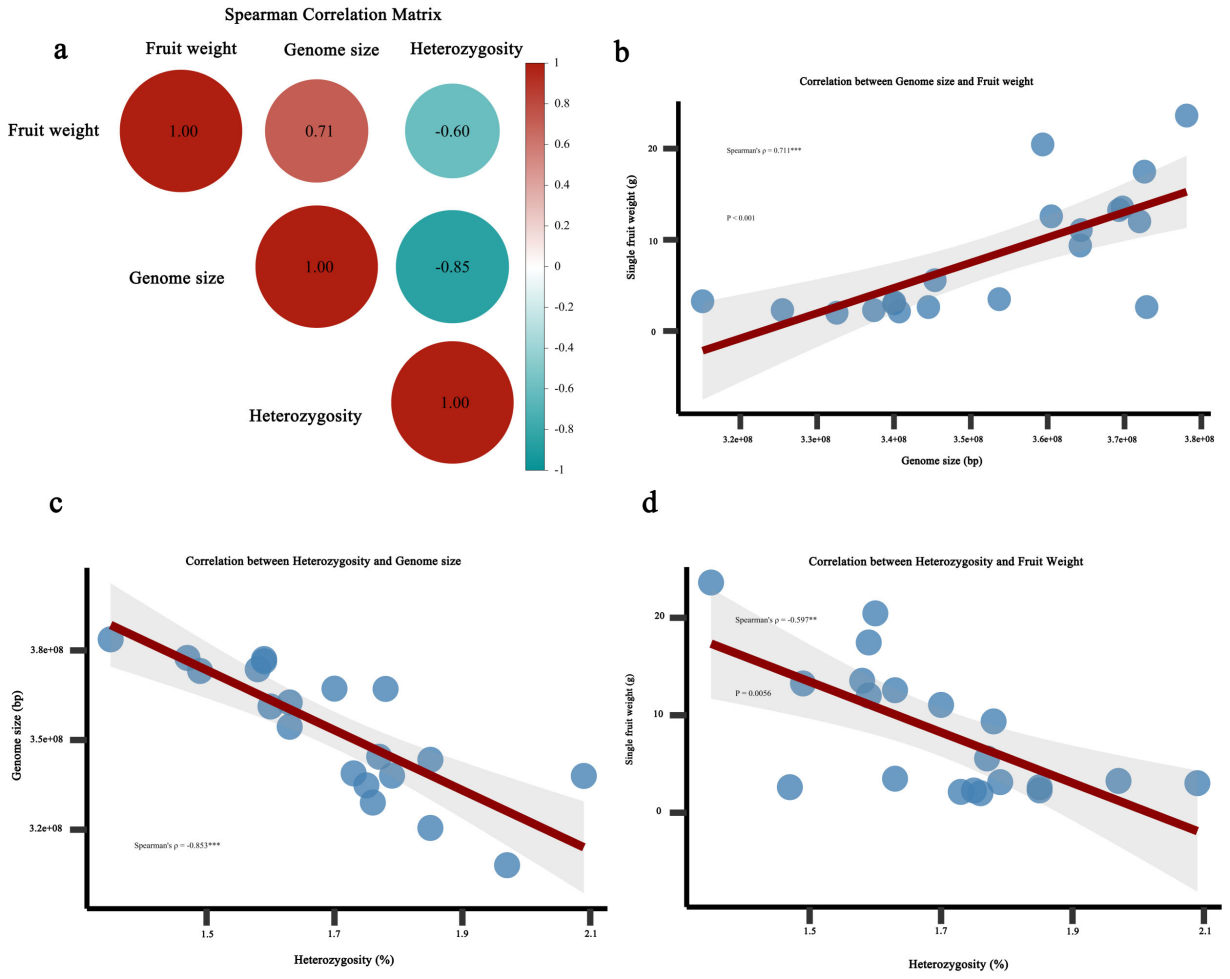
Correlations among fruit weight, genome size, and heterozygosity in jujube. **(a)** Spearman correlation matrix for the three traits, with color and circle size indicating the direction and magnitude of the correlation coefficient (ρ). **(b–d)** Scatter plots with linear regression lines and 95% confidence intervals for: **(b)** genome size vs. fruit weight, **(c)** heterozygosity vs. genome size, **(d)** heterozygosity vs. fruit weight.

To investigate the associations between genomic characteristics and agronomic traits, we performed correlation analyses between single fruit weight, genome size, and heterozygosity. Our analysis (**Figure 5**) revealed a significant negative correlation between fruit weight and heterozygosity (ρ = –0.597, P = 0.006), and a significant positive correlation between fruit weight and genome size (ρ = 0.711, P = 0.0004). Furthermore, an extremely strong negative correlation was observed between genome size and heterozygosity (ρ = –0.853, P = 1.93×10^-^^6^). This finding is consistent with the study by Wang et al. (2019) on 301 jujube cultivars, which reported a significant positive correlation between genome size and fruit weight-related traits. These results suggest that during jujube domestication, genome expansion may be linked to the artificial selection for increased fruit size, while the reduction in heterozygosity likely reflects the genetic bottleneck effect. These identified correlations preliminarily bridge the knowledge gap between genomic features and phenotypic traits, thereby enhancing the potential application value of our research in genomics-assisted breeding.

## 3 Discussion

### 3.1 The optimal sequencing depth and k-mer size for accurate estimation of genome size and heterozygosity in plants

Accurate estimation of genome size and heterozygosity is fundamental to understanding the genetic architecture and evolutionary history of a species. Previous studies have revealed considerable variation in the choice of k-mer size and sequencing depth for estimating these genomic parameters across different plant species. But most of such researches are focused on one or several species or genotypes. Li et al. (2019) employed k = 41 and 143.91× sequencing depth to estimate a genome size of ~254.4 Mbp and heterozygosity of 0.63% in *Apocynum venetum* (Apocynaceae), whereas Mgwatyu et al. (2020) used multiple k-values (17, 23, 27, 47) at 245× depth to estimate the genome of *Aspalathus linearis* (Fabaceae) at 1.03-1.24 Gb. Zhou et al. (2023) reported a genome size of ~553 Mb and heterozygosity of 1.93% for *Ilex pubescens* (Aquifoliaceae) using k = 21 and 82.2× depth, while Al-Qurainy et al. (2021) used the same k = 21 at depths of 243× and 255× to estimate genome sizes of ~867.7 Mb and ~896 Mb for *Reseda lutea* (Resedaceae) and *Reseda pentagyna* (Resedaceae) with heterozygosity rates of 1.04% and 1.37%, respectively. Pu et al. (2020) applied k = 17 and 90× depth to estimate the *Lonicera japonica* (Caprifoliaceae) genome at ~887.15 Mb with ~1.27% heterozygosity. Jia et al. (2019) used k = 17 and depths of 278× and 48× for two *Morella rubra* (Myricaceae) accessions, estimating a genome size of ~313 Mb and heterozygosity of 0.56-0.70%. More recently, Zeng et al. (2025a) reported a *Citrus changshan* (Rutaceae) genome size of ~326.65 Mb and heterozygosity of 3.07% using k = 21 and ~189× depth. Wei et al. (2024) estimated genome sizes of ~385.66 Mb and ~394.12 Mb with heterozygosity of 2.0-2.8% for two jujube cultivars using k = 21 and depths of 135× and 71×. Li et al. (2024a) estimated the genome sizes of *Malus asiatica ‘*Flame’ (Rosaceae) and *Malus asiatica* ‘Royalty’ (Rosaceae) to be 691.2 Mb and 685.4 Mb, respectively, using a K-mer value of 21 with sequencing depths of 100× and 133×. The genome heterozygosity rates were estimated at 2.89% for ‘Flame’ and 1.78% for ‘Royalty’. Huang et al. (2024) estimated the genome size of *Oryza rufipogon* ‘Y476’ (Poaceae) to be approximately 420 Mb and its heterozygosity rate to be 0.86%, using a K value of 21 and a sequencing depth of 70.9×. Li et al. (2025) applied k=43 and 126.24-179.18× depth to estimate five *Engelhardia roxburghiana* (Juglandaceae) with genome sizes of 724.5-1130.6 Mb and heterozygosity of 0.47-1.28%. Zhang et al. (2021) used k = 17 and 21.48× depth to estimate *Prunus armeniaca* ‘Yinxiangbai’ (Rosaceae) genome at ~264.4 Mb with 0.99% heterozygosity, and Yun et al. (2023) employed k = 17 and 21.5× depth to estimate a ~1 Gb genome in *Stipa breviflora* (Poaceae).

In summary, genome size and heterozygosity vary substantially across plant species, and their reliable estimation generally requires sequencing depths above 30× and k-mer sizes greater than 17. Our study demonstrates that the estimates of genome size and heterozygosity become stable in jujube in case sequencing depth beyond 30× and k-mer beyond 21, which has successfully applied in 510 accessions of both cultivated and wild jujube. Whether these parameters are suitable for other species depends on genomic features such as complexity, ploidy, repetitive content, and heterozygosity. For highly heterozygous or repeat-rich genomes, it may be necessary to increase both sequencing depth and k-mer size to improve the accuracy of genomic parameter estimation.

### 3.2 The genome size and heterozygosity of jujube compared with other plants

Extensive research has revealed substantial variation in genome size and heterozygosity across plant species. To evaluate the evolutionary position of the genomic characteristics of jujube (*Ziziphus jujuba*), the following section first presents the broad-spectrum variation background across the plant kingdom, with a focused comparison on taxa sharing similar biological characteristics. Genome sizes of plants range over several orders of magnitude, which can be roughly divided into five grades, i.e., extremely large (≥100 Gb), large (<100 Gb∼≥10 Gb), medium (<10 Gb∼≥1 Gb), medium-small (<1 Gb∼≥0.5 Gb), small (<0.5 Gb). By now, the largest one reported being that of *Tmesipteris oblanceolata* (Psilotaceae) with approximate 160.45 Gb (Fernández et al., 2024), and the smallest known plant genome being that of *Genlisea aurea* (Lentibulariaceae) with approximate 63.6 Mb (Leushkin et al., 2013). Other notably large genomes include *Paris polyphylla* var. *yunnanensis* (Melanthiaceae) at about 54.58 Gb (Zeng et al., 2025b), *Lilium sargentiae* (Liliaceae) at 35.66 Gb (Liang et al., 2025), *Pinaceae* (Pinaceae) ranging between 20 and 30 Gb (Nystedt et al., 2013), and *Picea abies* (Pinaceae) at roughly 20 Gb (Bernhardsson et al., 2019). Moderately large genomes, falling between 1 and 10 Gb, are exemplified by *Chrysanthemum morifolium* (Asteraceae) with 8.47-8.88 Gb (Song et al., 2023), *Gloriosa superba* (Colchicaceae) with 5.09 Gb (Liang et al., 2025), *Zea mays* ‘Mo17’ (Poaceae) with 2.18 Gb (Chen et al., 2023), and *Aspalathus linearis* (Fabaceae) with 1.03-1.24 Gb (Mgwatyu et al., 2020). The majority of plant species possess genomes below 1 Gb. For instance, *Engelhardia roxburghiana* (Juglandaceae) ranges from 724 to 858 Mb (Li et al., 2025), *Malus* ‘SH6’ (Rosaceae) genome at 649.37 Mb (Li et al., 2024b), and *Ilex pubescens* (Aquifoliaceae) is approximately 553 Mb (Zhou et al., 2023). Additional examples include *Vitis vinifera* (Vitaceae) at about 494.87 Mb (Wang et al., 2024), *Musa acuminata* (Musaceae) near 485 Mb (Belser et al., 2021), *Populus alba* ‘84K’ (Salicaceae) around 470.155 Mb (Qiu et al., 2019), *Citrullus lanatus* ‘97103’ (Cucurbitaceae) at 359.8 Mb (Guo et al., 2019), *Fragaria nilgerrensis* (Rosaceae) measuring 270.3 Mb (Zhang et al., 2020), *Prunus armeniaca* ‘Yinxiangbai’ (Rosaceae) estimated at 264.4 Mb (Zhang et al., 2021), and *Arabidopsis thaliana* (Brassicaceae) ranging from 135 to 155 Mb (Igolkina et al., 2025). Notably, the genomes of common fruit trees listed, such as apple (~650 Mb) and grape (~495 Mb), are significantly larger than the jujube genome estimated in this study (~409 Mb).

Genome heterozygosity levels also vary considerably among species in plants, which can be roughly grouped into five levels, i.e., extremely high (≥3%), high (<3%∼≥2%), medium (<2%∼≥1%), medium-low (<1%∼≥0.5%), low (<0.5%). Most plants exhibit heterozygosity below 4%. For example, *Prunus armeniaca* (Rosaceae) and *Prunus mume* (Rosaceae) show about 2,95% and 3.21% (Yang X. W. et al., 2024), while *Malus asiatica* ‘SH6’ (Rosaceae) displays 3.58%, respectively (Li et al., 2024a). *Lilium davidii* var. *unicolor* (Liliaceae) exhibits approximately 2.18% (Xu et al., 2024), *Solanum tuberosum* ‘RH89-039-16’ (Solanaceae) shows about 1.6% (Zhou et al., 2020), *Populus alba* ‘84K’ (Salicaceae) displays nearly 2.16% (Qiu et al., 2019), *Prunus persica* (Rosaceae) demonstrates around 1.33% (Yang X. W. et al., 2024), *Lonicera japonica* (Caprifoliaceae) shows about 1.27% (Pu et al., 2020), *Engelhardia roxburghiana* (Juglandaceae) ranges between 0.47% and 1.28% (Li et al., 2025), and *Malus domestica* (Rosaceae) varies from 0.85% to 1.28% (Sun et al., 2020). Many species demonstrate heterozygosity below 1%, such as *Prunus armeniaca* ‘Yinxiangbai’ (Rosaceae) at 0.99% (Zhang et al., 2021), *Prunus salicina* (Rosaceae) at 0.97% (Yang et al., 2024), *Oryza rufipogon* ‘Y476’ (Poaceae) at 0.86% (Huang et al., 2024), *Apocynum venetum* (Apocynaceae) species at 0.63% (Li et al., 2019), and *Fokienia hodginsii* (Cupressaceae) at 0.54% (Rong et al., 2024). *Wolffia australiana* (Araceae) represents an extreme case with a heterozygosity rate of less than 0.3% (Li et al., 2023). Among the woody fruit trees listed, the heterozygosity of species such as peach (~1.33%), apple (0.85%–1.28%), and plum (~0.97%) are all lower than that of jujube (1.30%–2.01%).

In comparison, the k-mer analysis in this study estimated the genome size of jujube to be approximately 409 Mb. This is in close agreement with the assembly result of the first jujube reference genome (437.65 Mb), jointly confirming that jujube possesses a relatively small genome among fruit trees. That reference genome study also indicated that the jujube genome is relatively small and highly complex, which is consistent with the relatively high heterozygosity level (1.30%–2.01%) observed in this study. Compared to Rosaceae fruit trees such as apple (~650 Mb) with a larger genome or peach (~0.3%–1.33%) with typically lower heterozygosity, jujube’s ‘small but complex’ genomic feature appears particularly distinctive. This genomic profile may stem from its evolutionary history, including adaptation to diverse environments and the synergistic interaction between its predominantly outcrossing reproductive system and the unique historical practice of widespread grafting for clonal propagation during domestication. This has enabled jujube to maintain a compact genome size while simultaneously fixing and transmitting advantageous heterozygous genotypes.

### 3.3 Exploration of the potential mechanisms underlying the negative correlation between genome size and heterozygosity

This study systematically estimated the genome size and heterozygosity of 296 accessions of cultivated jujube and wild sour jujubebased on K-mer analysis. Spearman correlation analysis revealed a significant negative correlation between genome size and heterozygosity across all samples (ρ = -0.436, p < 0.001). Further population stratification analysis showed that this negative correlation pattern independently existed and reached significance within both the cultivated jujube (ρ = -0.473, p < 0.001) and wild sour jujube (ρ = -0.527, p < 0.001) groups. This result suggests that the negative correlation between genome size and heterozygosity is not caused by population admixture but represents a common pattern formed during the domestication and long-term evolution of Ziziphus species. Its underlying mechanisms can be interpreted by integrating existing research findings on repetitive sequence characteristics, SNP density differentiation, and gene number variation.

#### 3.3.1 Divergence in repetitive sequence type and proportion mediates the negative correlation between genome size and heterozygosity

Repetitive sequences are the core factor driving genome size differentiation. In Ziziphus species, long terminal repeat (LTR) retrotransposons constitute the major component of genomic repetitive sequences, accounting for 35%-40% of the genome (Huang et al., 2016). Existing studies indicate that larger-genome jujube cultivars often contain a higher proportion of LTR retrotransposons during domestication, and these elements dominate the centromeric structure of the jujube genome (Tenaillon et al., 2010; Lin et al., 2025). Notably, repeat-rich regions such as centromeres typically exhibit lower recombination rates, which hinders recombination and exchange between alleles, thereby maintaining lower heterozygosity levels in these regions (Tenaillon et al., 2010). Furthermore, tandem repeats in the genome (e.g., centromeric satellite DNA, telomeric repeats) may be systematically underestimated in K-mer analysis due to assembly challenges (Wu et al., 2025). However, given that all samples in this study were analyzed using a uniform pipeline and considering the limited overall impact of such sequences on genome-wide heterozygosity estimation, the comparative results between groups remain reliable. The consistent significant negative correlation observed in both cultivated jujube and wild sour jujube groups further supports that differences in the type and content of repetitive sequences are a core factor mediating the association between genome size and heterozygosity.

#### 3.3.2 Association of SNP density differentiation with the negative correlation pattern

The population differentiation characteristics of SNP density provide another perspective for explaining the aforementioned negative correlation. The latest jujube domestication pangenome study confirmed that under strong artificial selection pressure, the average genome-wide nucleotide diversity (π) in cultivated jujube is significantly lower than in wild populations, and identified 126 selective sweep regions associated with domestication traits (Guo et al., 2024). This indicates that strong directional selection occurred in gene regions related to key agronomic traits such as fruit quality, leading to a systematic reduction in SNP density and heterozygosity in these regions. For wild sour jujube, genetic drift under small population effects can lead to random loss of SNP sites. Meanwhile, individuals with larger genomes tend to have a higher proportion of repetitive sequences, resulting in a relatively smaller proportion of effective variable regions (genic regions) and a more pronounced trend of reduced SNP density (Ferchaud et al., 2020; Liu et al., 2025). This may be an important reason why the negative correlation within the wild group (ρ = -0.527) is stronger than within the cultivated group (ρ = -0.473).

#### 3.3.3 Impact of gene number variation on genome dynamics during jujube domestication

Dynamic changes in gene number are an important feature of genome evolution during jujube domestication and are potentially linked to genome size and heterozygosity. The domestication process may lead to adaptive reshaping of gene families. For example, some gene families related to stress resistance may undergo contraction (Jiang et al., 2025), while those related to fruit quality (e.g., acidity regulation) may expand (Liu et al., 2024). Contraction of gene families is often accompanied by the loss of redundant genes, which not only makes the genome more compact but also, through the associated purifying selection process, reduces heterozygosity in these regions (Leushkin et al., 2013). On the other hand, although the expansion of certain gene families may increase genome size, the directional fixation of a few favorable alleles by artificial selection similarly leads to a decrease in heterozygosity within the expanded regions (Zhao et al., 2015). More critically, the amplification of repetitive sequences can physically displace functional gene space (Liu et al., 2025), leading to a decreased proportion of effective genic regions capable of accumulating SNP variation in the genome, thereby reducing overall SNP density. Therefore, the dynamic changes in gene number and gene families, by regulating the relative proportion of functional genic regions, indirectly mediate the negative correlation between genome size and heterozygosity. This is also an important manifestation of genome evolution during the domestication of jujube from wild to cultivated forms.

In summary, the negative correlation pattern between genome size and heterozygosity in Ziziphus species discovered in this study results from the combined effects of repetitive sequence differences, SNP density differentiation, and dynamic changes in gene number. Among these, artificial selection primarily drove the formation of this pattern in the cultivated jujube group, while genetic drift played a key role in the wild sour jujube group.

### 3.4 Clarifying the apparent paradox: higher distribution diversity in cultivated jujube

Study reveals that the distribution diversity (Shannon index) of both individual heterozygosity and genome size values within the cultivated jujube population is significantly higher than that within the wild sour jujube population. This section provides an integrated interpretation of this finding.

The cultivated population exhibits a broader distribution of individual values across two independent yet correlated genomic traits, strongly suggesting the presence of a universal driving force behind this pattern. We propose that this originates from the unique evolutionary dynamics of the jujube domestication system. Firstly, it is an open system with continuous genetic input. Gene flow, predominantly from wild populations to cultivated populations, consistently supplies cultivated varieties with genetic material encompassing diverse genomic structures and heterozygosity levels. This transforms the cultivated population’s gene pool into a dynamic “open system,” providing the material basis for the broadening of variation across multiple traits (Yang M. et al., 2024; Li et al., 2024). Secondly, diversified and strong artificial selection plays a core role. Human breeding objectives for jujube are highly diverse (e.g., large fruit, high sugar content, early ripening). To achieve these varied goals, breeders objectively select and fix genotypes with differing genome sizes and heterozygosity levels. For instance, selecting for larger fruit size may indirectly favor genomes with specific repeat sequence expansions (Huang et al., 2016), while utilizing heterosis (hybrid vigor) may fix individuals with high heterozygosity (Feldmann et al., 2024). This multi-targeted, high-intensity artificial selection acts like a “diversification engine,” synchronously driving the dispersion of multiple genomic trait values within the population.

In stark contrast, the distribution of both traits in the wild sour jujube population is more convergent. This reflects its distinct evolutionary context. On one hand, natural selection exerts a stabilizing filtering effect. In the wild environment, the fundamental selective pressures for survival and reproduction are relatively stable and intense, tending to filter out extreme phenotypes (including potentially maladapted extremely large/small genomes or abnormally high/low heterozygosity). This causes the population’s trait distribution to converge around an adaptive optimum. Even if gene introgression from cultivated varieties occurs, maladapted alleles are rapidly purged (Brown and Kelly, 2022). On the other hand, within the incompletely differentiated “sour jujube - jujube” system, the wild population primarily plays the dual role of a diversity “source” and a stabilizing “reservoir.” It serves as the source of variation output to the cultivated population, while itself being constrained by strong natural selection to maintain relative uniformity. The distribution diversity difference observed in this study is a direct manifestation of this dynamic equilibrium: the cultivated end rapidly diverges under human intervention, while the wild end remains stable through a balance of input and filtration (Li et al., 2024).

At the population level, the synergistic action of open gene flow and diversified strong selection jointly broadens the overall spectrum of variation for key genomic traits within the cultivated population. This demonstrates the capacity of artificial domestication to create and maintain a wide range of phenotypic and genotypic variation. At the individual level, despite the broadening of the variation range, the construction of an individual’s genome still follows profound intrinsic constraints, manifested as the stable negative correlation between genome size and heterozygosity. This reveals that, at the micro-scale, physical and evolutionary limitations of genomic structure (such as the suppression of recombination rate by repeat sequence amplification and the occupation of effective gene space) constitute a fundamental trade-off. Therefore, the domestication history of jujube is a dialectical process that liberates variational potential at the population level while reinforcing fundamental constraints at the individual level.

## Data Availability

The original contributions presented in the study are included in the article/**Supplementary Material**. Further inquiries can be directed to the corresponding author.

## Supplementary material

Supplementary Table 1 The estimated genome size and heterozygosity basing on NCBI resequencing data of jujube.

Supplementary Table 2 Estimation values of genome heterozygosity and genome size of 296 jujube varieties based on 30X genome resequencing.

Supplementary Table 3 Estimated genome heterozygosity and size of 214 wild jujube genotypes based on 30×genome resequencing.

Supplementary Table 4 Summary of Sequencing Output and Quality Control Metrics.

Supplementary Table 5 Sequencing Data Quality Assessment Summary for Variety 510.

Supplementary Table 6 Table of Model Fitting Parameters and Residual Results for Variety ‘510’.

## References

[B1] Al-Qurainy F. Gaafar A. Z. Khan S. Nadeem M. Alshameri A. M. Tarroum M. . (2021). Estimation of genome size in the endemic species Reseda pentagyna and the locally rare species Reseda lutea using comparative analyses of flow cytometry and k-mer approaches. Plants (Basel) 10, 1362. doi: 10.3390/plants10071362 34371565 PMC8309327

[B2] Belser C. Baurens F. C. Noel B. Martin G. Cruaud C. Istace B. . (2021). Telomere-to-telomere gapless chromosomes of banana using nanopore sequencing. Commun. Biol. 4, 1047. doi: 10.1038/s42003-021-02559-3 34493830 PMC8423783

[B3] Bernhardsson C. Vidalis A. Wang X. Scofield D. G. Schiffthaler B. Baison J. . (2019). An ultra-dense haploid genetic map for evaluating the highly fragmented genome assembly of Norway spruce (Picea abies). G3 (Bethesda) 9, 1623–1632. doi: 10.1534/g3.118.200840 30898899 PMC6505157

[B4] Biggiogera M. Cavallo M. Casali C. (2024). A brief history of the Feulgen reaction. Histochem. Cell Biol. 162, 3–12. doi: 10.1007/s00418-024-02279-9 38609528 PMC11227455

[B5] Brown K. E. Kelly J. K. (2022). Genome-wide association mapping of transcriptome variation in Mimulus guttatus indicates differing patterns of selection on cis- versus trans-acting mutations. Genetics 220, iyab189. doi: 10.1093/genetics/iyab189 34791192 PMC8733635

[B6] Čertnerová D. (2022). Meet the challenges of analyzing small genomes using flow cytometry. Cytometry Part A 101, 707–709. doi: 10.1002/cyto.a.24485 34302423

[B7] Chen M. J. Song X. B. Wu S. Yu A. J. Wei X. Qiu J. . (2025). Genomic insights into genome-wide heterozygosity and its impact on walnut adaptive evolution and improvement. Mol. Breed. 45, 50. doi: 10.1007/s11032-025-01572-2 40438424 PMC12106288

[B8] Chen J. Wang Z. J. Tan K. W. Huang W. Shi J. P. Li T. . (2023). A complete telomere-to-telomere assembly of the maize genome. Nat. Genet. 55, 1221–1231. doi: 10.1038/s41588-023-01419-6 37322109 PMC10335936

[B9] Feldmann M. J. Pincot D. D. A. Seymour D. K. Famula R. A. Jiménez N. P. López C. M. . (2024). A dominance hypothesis argument for historical genetic gains and the fixation of heterosis in octoploid strawberry. Genetics 228, iyae159. doi: 10.1093/genetics/iyae159 39385702 PMC11631417

[B10] Ferchaud A. L. Leitwein M. Laporte M. Boivin-Delisle D. Bougas B. Hernandez C. . (2020). Adaptive and maladaptive genetic diversity in small populations: Insights from the Brook Charr (Salvelinus fontinalis) case study. Mol. Ecol. 29, 3429–3445. doi: 10.1111/mec.15566 33463857

[B11] Fernández P. Amice R. Bruy D. Christenhusz M. J. M. Leitch I. J. Leitch A. L. . (2024). A 160 Gbp fork fern genome shatters size record for eukaryotes. iScience 27, 109889. doi: 10.1016/j.isci.2024.109889 39055604 PMC11270024

[B12] Greilhuber J. (2008). Cytochemistry and c-values: the less-well-known world of nuclear dna amounts. Ann. Bot. 101, 791–804. doi: 10.1093/aob/mcm250 17951594 PMC2710206

[B13] Guo M. Lian Q. Mei Y. Yang W. Zhao S. Zhang S. . (2024). Analyzes of pan-genome and resequencing atlas unveil the genetic basis of jujube domestication. Nat. Commun. 15, 9320. doi: 10.1038/s41467-024-53718-z 39472552 PMC11522667

[B14] Guo S. G. Zhao S. J. Sun H. H. Wang X. Wu S. Lin T. . (2019). Resequencing of 414 cultivated and wild watermelon accessions identifies selection for fruit quality traits. Nat. Genet. 51, 1616–1623. doi: 10.1038/s41588-019-0518-4 31676863

[B15] Hesse U. (2023). K-mer-based genome size estimation in theory and practice. Methods Mol. Biol. 2672, 79–113. doi: 10.1007/978-1-0716-3226-0_4 37335470

[B16] Huang J. F. Zhang Y. L. Li Y. P. Xing M. Lei C. L. Wang S. Z. . (2024). Haplotype-resolved gapless genome and chromosome segment substitution lines facilitate gene identification in wild rice. Nat. Commun. 15, 4573. doi: 10.1038/s41467-024-48845-6 38811581 PMC11137157

[B17] Huang J. Zhang C. Zhao X. Fei Z. Wan K. Zhang Z. . (2016). The jujube genome provides insights into genome evolution and the domestication of sweetness/acidity taste in fruit trees. PloS Genet. 12, e1006433. doi: 10.1371/journal.pgen.1006433 28005948 PMC5179053

[B18] Igolkina A. A. Vorbrugg S. Rabanal F. A. Liu H. J. Ashkenazy H. Kornienko A. E. . (2025). A comparison of 27 Arabidopsis thaliana genomes and the path toward an unbiased characterization of genetic polymorphism. Nat. Genet. 57, 2289–2301. doi: 10.1038/s41588-025-02293-0 40830656 PMC12425826

[B19] Jia H. M. Jia H. J. Cai Q. L. Wang Y. Zhao H. B. Yang W. F. . (2019). The red bayberry genome and genetic basis of sex determination. Plant Biotechnol. J. 17, 397–409. doi: 10.1111/pbi.12985 29992702 PMC6335074

[B20] Jiang F. Zhu X. Wu M. Chang P. Wu H. Li H. (2025). Domestication has reshaped gene families, gene expressions and flavonoid metabolites in green jujube (Ziziphus mauritiana Lam.) fruit. Horticulturae 11, 974. doi: 10.3390/horticulturae11080974 30654563

[B21] Kuo L. Y. Tang S. K. Kao T. T. Ebihara A. Fawcett S. Hsiao M. C. . (2021). A dormant resource for genome size estimation in ferns: c-value inference of the Ophioglossaceae using herbarium specimen spores. Appl. Plant Sci. 9, e11452. doi: 10.1002/aps3.11452 34938613 PMC8664048

[B22] Leushkin E. V. Sutormin R. A. Nabieva E. R. Penin A. A. Kondrashov A. S. Logacheva M. D. (2013). The miniature genome of a carnivorous plant Genlisea aurea contains a low number of genes and short non-coding sequences. BMC Genomics 14, 476. doi: 10.1186/1471-2164-14-476 23855885 PMC3728226

[B23] Li J. R. Cai H. C. Peng H. X. Deng Y. L. Zhou S. J. Tian J. . (2024b). The chromosome-level genome assembly of the dwarfing apple interstock Malus hybrid 'SH6'. Sci. Data 11, 552. doi: 10.1038/s41597-024-03405-x 38811578 PMC11136958

[B24] Li K. Chen R. Abudoukayoumu A. Wei Q. Ma Z. Wang Z. . (2024). Haplotype-resolved T2T reference genomes for wild and domesticated accessions shed new insights into the domestication of jujube. Hortic. Res. 11, uhae071. doi: 10.1093/hr/uhae071 38725458 PMC11079485

[B25] Li L. X. Crotty K. A. Kril J. J. Mccarthy S. W. Palmer A. A. (2000). Effect of melanin bleach on Feulgen-DNA microdensitometry in pigmented lesions. Anal. Quant. Cytol. Histol. 22, 150–154. 10800617

[B26] Li G. Q. Song L. X. Jin C. Q. Li M. Gong S. P. Wang Y. F. (2019). Genome survey and SSR analysis of Apocynum venetum. Biosci. Rep. 39, BSR20190146. doi: 10.1042/BSR20190146 31189745 PMC6591564

[B27] Li M. Su R. P. Cai X. Huang P. H. Fang O. Y. Song Y. G. . (2025). Is it time to abandon the flow cytometry in estimations of genome size when the k-mer analysis is available? The case of Engelhardia species. gComm 2, e013. doi: 10.48130/gcomm-0025-0014

[B28] Li F. Yang J. J. Sun Z. Y. Wang L. Qi L. Y. Sina A. . (2023). Plant-on-chip: core morphogenesis processes in the tiny plant Wolffia Australiana. PNAS Nexus 2, pgad141. doi: 10.1093/pnasnexus/pgad141 37181047 PMC10169700

[B29] Li H. Zhai X. Y. Peng H. X. Qing Y. Deng Y. L. Zhou S. J. . (2024a). Chromosomal level genome assemblies of two Malus crabapple cultivars 'Flame' and 'Royalty'. Sci. Data 11, 201. doi: 10.1038/s41597-024-03049-x 38351118 PMC10864326

[B30] Liang Y. W. Gao Q. Li F. Du Y. P. Wu J. Pan W. Q. . (2025). The giant genome of lily provides insights into the hybridization of cultivated lilies. Nat. Commun. 16, 45. doi: 10.1038/s41467-024-55545-8 39747119 PMC11696169

[B31] Liao X. Y. Zhu W. F. Liu C. Y. (2024). A high-precision genome size estimator based on the k-mer histogram correction. Front. Genet. 15, 1451730. doi: 10.3389/fgene.2024.1451730 39238787 PMC11374637

[B32] Lin D. Lan Y. Zhang Z. Guo J. Shen J. Wang G. . (2025). Structural composition and evolution of jujube centromere reveal a dominant role for LTR retrotransposon. Hortic. Res. 12, uhaf244. doi: 10.1093/hr/uhaf244 41209828 PMC12596083

[B33] Liu Q. Liu P. Wang S. Yang J. Dai L. Zheng J. . (2025). The dynamics of long terminal repeat retrotransposon proliferation and decay drive the evolution of genome size variation in Capsicum. Plants (Basel) 14, 2136–2153. doi: 10.3390/plants14142136 40733373 PMC12298681

[B34] Liu M. J. Wang J. R. Wang L. L. Liu P. Zhao J. Zhao Z. H. . (2020). The historical and current research progress on jujube-a superfruit for the future. Hortic. Res. 7, 119. doi: 10.1038/s41438-020-00346-5 32821402 PMC7395136

[B35] Liu H. Zhao X. Bi J. Dong X. Zhang C. (2024). A natural mutation in the promoter of the aconitase gene ZjACO3 influences fruit citric acid content in jujube. Hortic. Res. 11, uhae003. doi: 10.1093/hr/uhae003 38464475 PMC10923642

[B36] Liu M. J. Zhao J. Cai Q. L. Liu G. C. Wang J. R. Zhao Z. H. . (2014). The complex jujube genome provides insights into fruit tree biology. Nat. Commun. 5, 5315. doi: 10.1038/ncomms6315 25350882 PMC4220462

[B37] Mello M. L. S. Vidal B. C. (2017). The Feulgen reaction: a brief review and new perspectives. Acta Histochem. 119, 603–609. doi: 10.1016/j.acthis.2017.07.002 28739089

[B38] Mgwatyu Y. Stander A. A. Ferreira S. Williams W. Hesse U. (2020). Rooibos (Aspalathus linearis) genome size estimation using flow cytometry and k-mer analyses. Plants (Basel) 9, 270. doi: 10.3390/plants9020270 32085566 PMC7076435

[B39] Mochizuki T. Sakamoto M. Tanizawa Y. Nakayama T. Tanifuji G. Kamikawa R. . (2023). A practical assembly guideline for genomes with various levels of heterozygosity. Brief Bioinform. 24, bbad337. doi: 10.1093/bib/bbad337 37798248 PMC10555665

[B40] Moeckel C. Mareboina M. Konnaris M. A. Chan C. S. Y. Mouratidis I. Montgomery A. . (2024). A survey of k-mer methods and applications in bioinformatics. Comput. Struct. Biotechnol. J. 23, 2289–2303. doi: 10.1016/j.csbj.2024.05.025 38840832 PMC11152613

[B41] Nix J. Ranney T. G. Lynch N. P. Chen H. (2024). Flow cytometry for estimating plant genome size: revisiting assumptions, sources of variation, reference standards, and best practices. J. Am. Soc Hortic. Sci. 149, 131–141. doi: 10.21273/jashs05376-24

[B42] Nystedt B. Street N. R. Wetterbom A. Zuccolo A. Lin Y. C. Scofield D. G. . (2013). The Norway spruce genome sequence and conifer genome evolution. Nature 497, 579–584. doi: 10.1038/nature12211 23698360

[B43] Pu X. D. Li Z. Tian Y. Gao R. R. Hao L. J. Hu Y. T. . (2020). The honeysuckle genome provides insight into the molecular mechanism of carotenoid metabolism underlying dynamic flower coloration. New Phytol. 227, 930–943. doi: 10.1111/nph.16552 32187685 PMC7116227

[B44] Qiu D. Y. Bai S. L. Ma J. C. Zhang L. S. Shao F. J. Zhang K. K. . (2019). The genome of Populus alba x Populus tremula var. glandulosa clone 84K. DNA Res. 26, 423–431. doi: 10.1093/dnares/dsz020 31580414 PMC6796506

[B45] Roberts M. D. Davis O. Josephs E. B. Williamson R. J. (2025). K-mer-based approaches to bridging pangenomics and population genetics. Mol. Biol. Evol. 42, msaf047. doi: 10.1093/molbev/msaf047 40111256 PMC11925024

[B46] Rong J. D. Zheng Y. S. Zhang Z. Y. Zhang J. Gu Y. Y. Hua T. . (2024). De novo whole-genome assembly of the 10-gigabase Fokienia hodginsii genome to reveal differential epigenetic events between callus and xylem. Adv. Sci. (Weinh.) 11, e2402644. doi: 10.1002/advs.202402644 39229940 PMC11516051

[B47] Schmidt S. Alanko J. N. (2023). Eulertigs: minimum plain text representation of k-mer sets without repetitions in linear time. Algorithms Mol. Biol. 18, 5. doi: 10.1186/s13015-023-00227-1 37403080 PMC10318842

[B48] Song A. P. Su J. S. Wang H. B. Zhang Z. R. Zhang X. T. Van de Peer Y. . (2023). Analyses of a chromosome-scale genome assembly reveal the origin and evolution of cultivated chrysanthemum. Nat. Commun. 14, 2021. doi: 10.1038/s41467-023-37730-3 37037808 PMC10085997

[B49] Sun X. P. Jiao C. Schwaninger H. Chao C. T. Ma Y. M. Duan N. B. . (2020). Phased diploid genome assemblies and pan-genomes provide insights into the genetic history of apple domestication. Nat. Genet. 52, 1423–1432. doi: 10.1038/s41588-020-00723-9 33139952 PMC7728601

[B50] Tenaillon M. I. Hollister J. D. Gaut B. S. (2010). A triptych of the evolution of plant transposable elements. Trends Plant Sci. 15, 471–478. doi: 10.1016/j.tplants.2010.05.003 20541961

[B51] Wang Y. Ding K. Y. Li H. Y. Kuang Y. F. Liang Z. C. (2024). Biography of vitis genomics: recent advances and prospective. Hortic. Res. 11, uhae128. doi: 10.1093/hr/uhae128 38966864 PMC11220177

[B52] Wang J. Liu J. Kang M. (2015). Quantitative testing of the methodology for genome size estimation in plants using flow cytometry: a case study of the Primulina genus. Front. Plant Sci. 6, 354. doi: 10.3389/fpls.2015.00354 26042140 PMC4436564

[B53] Wang L. Luo Z. Liu Z. Zhao J. Deng W. Wei H. . (2019). Genome size variation within species of Chinese jujube (Ziziphus jujuba Mill.) and its wild ancestor sour jujube (Z. acidojujuba Cheng et Liu). Forests 10, 460. doi: 10.3390/f10050460 30654563

[B54] Wei T. J. Li H. Huang X. S. Yang P. (2024). Chromosome-level genome assembly of two cultivated jujubes. Sci. Data 11, 1144. doi: 10.1038/s41597-024-03992-9 39420037 PMC11486999

[B55] Wu H. Blanca A. Medvedev P. (2025). A k-mer-based estimator of the substitution rate between repetitive sequences. 20, 1–20. doi: 10.1101/2025.06.19.660607 PMC13087736 42006138

[B56] Xu S. Chen R. Zhang X. Wu Y. Yang L. Sun Z. . (2024). The evolutionary tale of lilies: Giant genomes derived from transposon insertions and polyploidization. Innovation (Camb) 5, 100726. doi: 10.1016/j.xinn.2024.100726 39529947 PMC11551468

[B57] Yan H. H. Martin S. L. Bekele W. A. Latta R. G. Diederichsen A. Peng Y. Y. . (2016). Genome size variation in the genus Avena. Genome 59, 209–220. doi: 10.1139/gen-2015-0132 26881940

[B58] Yang M. Han L. Zhang S. F. Dai L. Li B. Han S. K. . (2023). Insights into the evolution and spatial chromosome architecture of jujube from an updated gapless genome assembly. Plant Commun. 4, 100662. doi: 10.1016/j.xplc.2023.100662 37482683 PMC10777365

[B59] Yang X. W. Su Y. Huang S. Y. Hou Q. D. Wei P. C. Hao Y. N. . (2024). Comparative population genomics reveals convergent and divergent selection in the apricot-peach-plum-mei complex. Hortic. Res. 11, uhae109. doi: 10.1093/hr/uhae109 38883333 PMC11179850

[B60] Yang M. Zhang S. F. Li B. Lan Y. X. Yang Y. H. Liu M. J. (2024). Comparative analysis of 326 chloroplast genomes in Chinese jujube (Ziziphus jujuba): Structural variations, horizontal gene transfer events, and evolutionary patterns impacting its domestication from wild jujube. J. Syst. Evol. 62, 1069–1084. doi: 10.1111/jse.13065 40046247

[B61] Yun X. J. Wu J. R. Xu B. Lv S. J. Zhang L. Zhang W. G. . (2023). Genome survey of Stipa breviflora Griseb. using next-generation sequencing. Agriculture 13, 2243. doi: 10.3390/agriculture13122243 30654563

[B62] Zeng Z. H. Luo Y. J. Hu H. F. Lan L. Guo B. J. Zhou P. . (2025b). Highly heterozygous Citrus changshan-huyou Y. B. Chang originated from ancient hybridization between mandarin and pummelo and displayed distinct tissue-specific allelic imbalance. doi: 10.1101/2025.03.24.644872 PMC13028926 41894487

[B63] Zeng P. Zong H. Han Y. W. Zhang W. X. Tian Z. Z. Zhou B. T. . (2025a). Two Melanthiaceae genomes with dramatic size difference provide insights into giant genome evolution and maintenance. Nat. Plants 11, 1500–1513. doi: 10.1038/s41477-025-02060-3 40750694

[B64] Zhang J. X. Lei Y. Y. Wang B. T. Li S. Yu S. Wang Y. . (2020). The high-quality genome of diploid strawberry (Fragaria nilgerrensis) provides new insights into anthocyanin accumulation. Plant Biotechnol. J. 18, 1908–1924. doi: 10.1111/pbi.13351 32003918 PMC7415782

[B65] Zhang Q. P. Zhang D. Y. Yu K. Ji J. J. Liu N. Zhang Y. P. . (2021). Frequent germplasm exchanges drive the high genetic diversity of Chinese-cultivated common apricot germplasm. Hortic. Res. 8, 215. doi: 10.1038/s41438-021-00650-8 34593777 PMC8484454

[B66] Zhao S. Zheng F. He W. Wu H. Pan S. Lam H. M. (2015). Impacts of nucleotide fixation during soybean domestication and improvement. BMC Plant Biol. 15, 81. doi: 10.1186/s12870-015-0463-z 25849896 PMC4358728

[B67] Zhou Q. Tang D. Huang W. Yang Z. M. Zhang Y. Hamilton J. P. . (2020). Haplotype-resolved genome analyses of a heterozygous diploid potato. Nat. Genet. 52, 1018–1023. doi: 10.1038/s41588-020-0699-x 32989320 PMC7527274

[B68] Zhou P. Zhang Q. Li J. Li F. Huang J. Zhang M. (2023). A first insight into the genomic background of Ilex pubescens (Aquifoliaceae) by flow cytometry and genome survey sequencing. BMC Genomics 24, 270. doi: 10.1186/s12864-023-09359-5 37208610 PMC10197237

