# Supplementary material for: Identification of genome size and heterozygosity in 510 Jujube (Ziziphus jujuba Mill.) germplasms based on deep resequencing
Source: Front Plant Sci. 2026 Jul 16;17:1777223. doi: 10.3389/fpls.2026.1777223 (PMC13422227; doi:10.3389/fpls.2026.1777223)
Supplement: Supplementary Table 1 — The estimated genome size and heterozygosity basing on NCBI resequencing data of jujube. [file Table1.pdf]

Supplementary materials

Supplementary table 1 The estimated genome size and heterozygosity basing on NCBI resequencing data of jujube

Accession number	Genomic heterozygosity(%)	Genome size（bp）	Sequencing multiplier（×）	geo_loc_name	Variety name	Accession number	Genomic heterozygosity（%）	Genome size（bp）	Sequencing multiplier（×）	geo_loc_name	Variety name
SRR10052694	1.2	390573609	34	China	Suanzao-92	SRR10053063	9.55	106317530	15	China	Nanjingyazao
SRR10052695	1.24	389355393	31	China	Suanzao-91	SRR13440687	9.55	101326214	15	China: Qingjian\, Shaanxi	Wulongzhuyaozao
SRR26434559	1.25	349344775	103	China:Shaanxi	Junzao	SRR10053014	9.56	101082486	16	China	Zaoqiangcuizao
SRR28513585	1.28	324231176	93	not collected	YuhongJiaocheng 5	SRR10053031	9.56	97255674	14	China	Suanzao-234
SRR26434558	1.33	357335557	114	China:Shaanxi	Qingjian wild jujube	SRR13440586	9.56	105249859	15	China: Taigu\, Shanxi	Yuanling1Hao
SRR28513584	1.33	328754638	62	not collected	YuhongJiaocheng 5	SRR13440630	9.56	100581046	14	China: Taigu\, Shanxi	Xinzhengjixinzao
SRR10053079	1.36	399346365	36	China	Suanzao-28	SRR10052802	9.57	103723638	15	China	Shaanxiqiyuexian
SRR3095673	1.4	361125664	32	China:Taigu\, Shanxi Province	Zhongyangmuzao	SRR10052863	9.57	106962092	16	China	Suanzao-69
SRR3095687	1.59	356907076	30	China:Qingjian\, Shaanxi Province	Xingtaisuanzao_5	SRR13440607	9.58	106278651	16	China: Taigu\, Shanxi	Linyilajiaozao
SRR3095679	1.66	372599889	30	China:Qingjian\, Shaanxi Province	Dayesuanzao	SRR10052867	9.59	111155693	16	China	Suanzao-65
SRR3095682	1.68	365861750	33	China:Qingjian\, Shaanxi Province	Tiansuanzao	SRR13440632	9.59	100365543	14	China: Taigu\, Shanxi	Taigudasuanzao
SRR3095656	2.17	297695334	27	China:Taigu\, Shanxi	huizao	SRR13440726	9.59	98107748	14	China: Taigu\, Shanxi	Lianxiantangzao

				Province						
SRR3095662	2.23	288130573	28	China:Taigu\, Shanxi Province	Zhongningxiaoyuanzao	SRR10052721	9.6	100004570	14	China	Suanzao-249
SRR3095683	2.25	264313156	28	China:Qingjian\, Shaanxi Province	Qingjiansuanzao	SRR10052919	9.6	103215467	16	China	Xupudaguosuanpanzao
SRR3095667	2.28	306732582	19	China:Taigu\, Shanxi Province	Jinzao	SRR10053007	9.6	100834161	15	China	Neihuangbianhesuan
SRR3095653	2.31	280616463	25	China:Taigu\, Shanxi Province	Jinsixiaozao	SRR13440588	9.6	99284425	14	China: Taigu\, Shanxi	Dalibashenghu
SRR3095660	2.31	279399953	25	China:Taigu\, Shanxi Province	Dunhuangdazao	SRR13440603	9.6	100627329	14	China: Taigu\, Shanxi	Yunchengxiangzao
SRR3095649	2.4	271651235	24	China:Taigu\, Shanxi Province	Linyilizao	SRR10052622	9.61	99852972	14	China	Suanzao-156
SRR3095655	2.4	276928279	25	China:Taigu\, Shanxi Province	Yuanlingzao	SRR10052720	9.61	104273388	15	China	Xiajinmamazao
SRR13440609	2.46	4299764	12	China: Taigu\, Shanxi	Yutianxiaozao	SRR13440752	9.61	96611509	13	China: Taigu\, Shanxi	Sunanbaipuzao
SRR3095676	2.47	262357834	26	China:Qingjian\, Shaanxi Province	Jiaxianguoduxing	SRR10052594	9.62	106112537	15	China	Suanzao-44
SRR3095685	2.47	308698368	23	China:Taigu\, Shanxi Province	Xinjiangxiaoyuanzao	SRR13440624	9.62	102084280	14	China: Taigu\, Shanxi	Zhenpingtailihong
SRR10052868	2.51	5327043	12	China	Suanzao-213	SRR10052582	9.63	97083388	14	China	Suanzao-241
SRR3095677	2.52	262873191	27	China:Qingjian\,	Yanchuandasuanzao	SRR10052680	9.63	100875245	14	China	Suanzao-217

SRR3095678	2.55	271797239	23	Shaanxi Province China:Qingjian\, Shaanxi Province	Jiaxiantuansuanzao	SRR10052938	9.63	109247607	15	China	Tai'anlingzao
SRR3095671	2.56	296572482	25	China:Taigu\, Shanxi Province	Xishuangbannaxiaozao	SRR10052978	9.63	106027861	15	China	Jinxianmuzao
SRR3081197	2.61	6017704	17	China:Shaanxi Province	Junzao	SRR13440610	9.63	102389206	14	China: Taigu\, Shanxi	Tengzhouchanghong
SRR3095650	2.66	270367524	22	China:Taigu\, Shanxi Province	Qiyuexian	SRR10052710	9.64	113050537	15	China	Suanzao-77
SRR10052668	2.87	7305087	13	China	Suanzao-115	SRR10052717	9.64	111339293	17	China	Lengbaiyu
SRR3095669	2.98	276328482	20	China:Taigu\, Shanxi Province	Yongjihamazao	SRR10053008	9.64	107275593	15	China	Xinzhengjixinzao
SRR10052827	3.09	4719502	15	China	Suanzao-199	SRR10053044	9.64	104809350	14	China	Shaoguangdazao
SRR10052810	3.14	6471154	14	China	Yuciyazao	SRR10053094	9.64	104252160	14	China	Suanzao-208
SRR10053018	3.31	7005714	15	China	Xinledazao	SRR13440689	9.64	102182000	14	China: Taigu\, Shanxi	Jiaxiandayesuanzao
SRR13440737	3.38	8006916	12	China: Taigu\, Shanxi	Chaoyangjianjianzao	SRR13440733	9.64	91682584	13	China: Taigu\, Shanxi	Akesuxiaozao
SRR10052706	3.4	8927271	15	China	Suanzao-81	SRR10052635	9.65	93498223	13	China	Suanzao-221
SRR10052611	3.48	9355897	14	China	Suanzao-166	SRR10052918	9.65	105607187	15	China	Hengyangzhenzhuzao
SRR13440664	3.51	8625195	10	China: Qingjian\, Shaanxi	Xingtai No.32	SRR10053013	9.65	101433034	16	China	Xianxianxiaodazao
SRR10052961	3.71	10105850	13	China	Linyibobozao	SRR10052772	9.66	101098428	15	China	Taigumeimizao
SRR10052778	3.8	8907683	16	China	Yongjihamazao	SRR10052848	9.66	117170327	17	China	Suanzao-180
SRR13440674	4	8102185	13	China:	Xingtai No.25	SRR10052962	9.67	104978628	15	China	Wanrongfu

				Qingjian\, Shaanxi							zao
SRR10052728	4.05	8273017	15	China	Xiajinchahuzao	SRR13440682	9.67	100490455	14	China: Qingjian\, Shaanxi	Xingtai No.14
SRR10052768	4.07	8611205	13	China	Baodeyouzao	SRR13440699	9.67	102891284	15	China: Qingjian\, Shaanxi	Suanpoyi
SRR10053026	4.08	7572006	16	China	Xincaidayuanfeng	SRR13440744	9.67	106285662	15	China: Taigu\, Shanxi	Kashigedongxiaozao
SRR13440685	4.09	10178477	13	China: Taigu\, Shanxi	Jinancuisuanzao	SRR10052805	9.68	103436659	15	China	Xiaxianyuancuizao
SRR10052699	4.13	10289403	13	China	Suanzao-87	SRR13440650	9.68	100517471	13	China: Taigu\, Shanxi	Pingshunjunzao
SRR13440598	4.13	7971575	11	China: Taigu\, Shanxi	Zhenpingguangyangzao	SRR13440763	9.68	92178764	14	China: Taigu\, Shanxi	Xupuxiaoguosuanpan
SRR13440618	4.13	7853286	11	China: Taigu\, Shanxi	Binxianheigeda	SRR10052623	9.69	101544612	15	China	Suanzao-155
SRR3081153	4.18	10526835	17	China:Shaanxi Province	Junzao	SRR10052895	9.69	109020709	16	China	Langxiniunaizao
SRR10052702	4.2	10102546	12	China	Suanzao-215	SRR13440658	9.69	102406813	14	China: Qingjian\, Shaanxi	Xingtai No.43
SRR13440571	4.2	7266946	12	China: Taigu\, Shanxi	Qufuhoutouzao	SRR13440659	9.69	103799041	14	China: Qingjian\, Shaanxi	Xingtai No.39
SRR10052934	4.27	12544783	17	China	Suanzao-246	SRR13440706	9.69	97439667	14	China: Qingjian\, Shaanxi	Lingwuchangzao
SRR10052813	4.29	9206534	13	China	Suanzao-204	SRR10052639	9.7	102850812	14	China	Suanzao-142
SRR10052679	4.37	14082692	13	China	Suanzao-105	SRR10052782	9.72	109185097	15	China	Pinglutuntunzao
SRR10052784	4.45	7444431	15	China	Taiguheiyezao	SRR10052777	9.74	110720275	17	China	Junzao
SRR13440602	4.46	8277208	12	China: Taigu\, Shanxi	Cangxianjinsixiaozao	SRR10052793	9.74	108107025	15	China	Yanchuanniunaizao

SRR13440740	4.46	6832805	12	China: Taigu\, Shanxi	Cangxianjinsixiaozao	SRR10052897	9.74	110888060	15	China	Fuyangmutouzao
SRR13440646	4.62	6831545	12	China: Taigu\, Shanxi	Zhongyangtuanzao	SRR10052989	9.75	110356851	15	China	Hetaowen-1
SRR10052893	4.72	11336351	14	China	Yiwu e'zizao	SRR10053064	9.75	109107826	15	China	Zhongningxiaoyuanzao
SRR10053081	4.72	8327077	19	China	Suanzao-26	SRR10052566	9.76	108650610	15	China	Yucimianmeizao
SRR10053083	4.73	8765262	12	China	Suanzao-209	SRR10052943	9.76	102091861	14	China	Zhenpingjiuyuehan
SRR10052790	4.77	8415045	14	China	Suanzao-233	SRR10052980	9.76	104229651	15	China	Yutianxiaozao
SRR10052608	4.85	14277841	16	China	Suanzao-169	SRR10052593	9.77	109411701	15	China	Xinzhengjiantouhuizao
SRR10052754	4.98	13782499	16	China	Jishanliuguanzao	SRR10052723	9.77	108839334	15	China	Fulingjidanzao
SRR10053059	5.02	12705504	16	China	Linzedazao	SRR10052794	9.77	113011900	15	China	Dalishuizao
SRR10052587	5.16	12603800	16	China	Jinzao	SRR10052949	9.77	108189360	16	China	Chengwudongzao
SRR10052807	5.18	13973754	16	China	Hongzhaoxiaozao	SRR10053023	9.77	108689644	15	China	Gutouxiaozao
SRR10052816	5.19	13608140	15	China	Zhongyangtuanzao	SRR10053095	9.77	105314809	15	China	Suanzao-14
SRR10053078	5.23	19326324	17	China	Suanzao-29	SRR13440651	9.77	105806545	14	China: Taigu\, Shanxi	Pingyaokudongzao
SRR10052602	5.24	15406535	16	China	Suanzao-36	SRR13440693	9.77	103744814	14	China: Qingjian\, Shaanxi	Jiaxiantuansuanzao
SRR10052852	5.37	15085868	14	China	Suanzao-176	SRR10052700	9.78	114210697	15	China	Suanzao-86
SRR10052580	5.45	15550534	17	China	Hamidazao	SRR10052901	9.78	108570971	15	China	Xupumuzao
SRR13440665	5.45	16508657	11	China: Qingjian\, Shaanxi	Xingtai No.33	SRR13440739	9.78	120013161	16	China: Taigu\, Shanxi	Chaoyangdajianding
SRR13440681	5.49	18013106	10	China: Qingjian\, Shaanxi	Xingtai No.15	SRR10052714	9.79	108413963	15	China	Tianjin No.1
SRR10052842	5.64	20826072	13	China	Suanzao-185	SRR10052736	9.79	109989833	15	China	Songxiandazao
SRR10052838	5.67	21171339	13	China	Suanzao-189	SRR10052860	9.79	118799164	16	China	Suanzao-72
SRR10053090	7.02	49609177	19	China	Suanzao-18	SRR10053068	9.79	108322026	15	China	Gansudongzao

SRR10052708	7.09	63832808	13	China	Suanzao-79	SRR10053098	9.79	107843499	15	China	Suanzao-11
SRR10053086	7.27	50530902	18	China	Suanzao-22	SRR10052669	9.8	107547844	14	China	Suanzao-218
SRR13440769	7.27	60019139	11	China: Qingjian\, Shaanxi	Tianjingagazao	SRR10052718	9.8	112928718	16	China	Mayabaizao
SRR13440579	7.33	50122554	10	China: Taigu\, Shanxi	Jiaxianmidiancuimuzao	SRR10052823	9.8	106691522	15	China	Taiguduanzizao
SRR13440701	7.34	51172117	10	China: Qingjian\, Shaanxi	Ankangmizao	SRR10052920	9.81	113819960	15	China	Xupuchengtuozao
SRR10052954	7.41	53609725	16	China	Linyitiansuanzao	SRR10052942	9.81	113633134	15	China	Ningyangliuyuexian
SRR10052591	7.42	53224081	11	China	Shanxihuluzao	SRR10053067	9.81	113496763	15	China	Ningxiadahongzao
SRR10053047	7.5	60683511	12	China	Yixianmuzao	SRR10052646	9.82	100985289	14	China	Suanzao-135
SRR13440758	7.5	51816379	10	China: Taigu\, Shanxi	Lanximazao	SRR10052904	9.82	111252647	15	China	Xupuxiangzao
SRR3095689	7.55	53893079	10	China:Qingjian\, Shaanxi Province	Xingtaisuanzao_11	SRR13440668	9.82	105266206	14	China: Qingjian\, Shaanxi	Xingtai No.28
SRR13440721	7.56	58491115	11	China: Taigu\, Shanxi	Linyibenzao	SRR10052660	9.83	111597176	15	China	Suanzao-123
SRR10052939	7.58	57002950	11	China	Tai'angedazao	SRR10052750	9.83	111091644	15	China	Dingxiangxingxingzao
SRR10053045	7.65	62798669	12	China	Shaoguanbaizao	SRR10052791	9.83	108216117	16	China	Tengzhoutangzao
SRR10053060	7.7	62127745	12	China	Guanyangduanzao	SRR10052945	9.83	124386954	17	China	Suanzao-245
SRR13440670	7.7	55671600	10	China: Qingjian\, Shaanxi	Xingtai No.29	SRR10052963	9.83	113223994	15	China	Shanxishouxingzao
SRR13440680	7.71	58204347	11	China: Qingjian\, Shaanxi	Xingtai No.16	SRR10052726	9.84	114339364	16	China	Tengzhoudamazao
SRR10053066	7.73	58567809	14	China	Zhongningdiaolingzao	SRR10052996	9.84	109813847	15	China	Shaanxinaizao
SRR10053009	7.75	67398408	12	China	Suanzao-236	SRR10053071	9.84	110964142	15	China	Yiwumianxuzao

SRR13440686	7.75	55175074	11	China: Qingjian\, Shaanxi	Lingbaoyuanzao/Lingbaodazao	SRR13440656	9.84	100527750	14	China: Qingjian\, Shaanxi	Xingtai No.44
SRR13440728	7.75	63603980	11	China: Taigu\, Shanxi	Lianxianmuzao	SRR10052592	9.85	108897325	16	China	Puyangtangzao
SRR13440713	7.77	63952994	11	China: Taigu\, Shanxi	Beijingdalaohuyansuanzao	SRR10052870	9.85	123544725	16	China	Suanzao-63
SRR10052929	7.78	59752653	12	China	Dalilinqinzao	SRR10052898	9.85	118894616	16	China	Lanximazao
SRR10052574	7.79	66141955	12	China	Dingxiangyouhezao	SRR10052905	9.85	111097501	15	China	Xupuyanzao
SRR10052769	7.79	57972374	12	China	Lichengxiaozao	SRR10052968	9.86	113367321	16	China	Daliyuanzao
SRR13440595	7.8	54660572	10	China: Taigu\, Shanxi	Yanchuandieyazao	SRR10052983	9.86	114808412	15	China	Dayewuhe
SRR13440698	7.81	60220281	11	China: Qingjian\, Shaanxi	Xingtai No.5	SRR10052615	9.87	111719635	15	China	Suanzao-163
SRR13440626	7.83	63043045	12	China: Taigu\, Shanxi	Xinzhengdamaya	SRR10052633	9.87	110225401	15	China	Suanzao-146
SRR13440782	7.83	60239402	11	China: Taigu\, Shanxi	Taigulangzao	SRR10052688	9.87	108919527	15	China	Suanzao-97
SRR3081344	7.84	49285866	10	China:Shaanxi Province	Junzao	SRR10052701	9.87	113979935	15	China	Suanzao-85
SRR13440761	7.86	60369006	11	China: Taigu\, Shanxi	Xupuxiangzao	SRR10052811	9.87	104010884	15	China	Jiaochengduanzao
SRR10053054	7.88	65009918	12	China	Xinjiangxiaoyuanzao	SRR13440735	9.87	111977052	15	China: Taigu\, Shanxi	Chaoyangxiaopingding
SRR13440661	7.88	60270957	11	China: Qingjian\, Shaanxi	Xingtai No.38	SRR10052573	9.88	114778829	16	China	Taiyuanyuanzao
SRR13440770	7.88	61568131	11	China: Taigu\, Shanxi	Tianjinkuaizao	SRR10052908	9.88	106203622	16	China	Xiaoguosuanpanzao
SRR13440709	7.9	65253716	11	China: Qingjian\,	Yanliangxiangzao	SRR10052957	9.88	115919734	16	China	Yongjijidanzao

				Shaanxi							
SRR13440723	7.91	64577203	11	China: Taigu\, Shanxi	Linfenmugeda	SRR10052984	9.88	105605366	14	China	Xuechengdongzao
SRR13440742	7.92	59899069	11	China: Taigu\, Shanxi	Minqinxiaozao	SRR10053061	9.89	110531224	15	China	Suanzao-254
SRR13440577	7.93	63649880	12	China: Taigu\, Shanxi	Heyanglinglingzao	SRR13440614	9.89	108632145	15	China: Taigu\, Shanxi	Pinglujianzao
SRR13440741	7.95	61189018	11	China: Taigu\, Shanxi	Chaoyangxiaoyuanling	SRR10052585	9.9	109484049	16	China	Dalibashenghu
SRR13440766	7.96	66809472	12	China: Taigu\, Shanxi	Xupuchengtuozao	SRR10052590	9.9	111671338	16	China	Yanchuandieyazao
SRR13440585	7.99	65402170	11	China: Taigu\, Shanxi	Taiansuyuanling	SRR10052692	9.9	103906589	14	China	Suanzao-94
SRR13440712	7.99	68772694	12	China: Taigu\, Shanxi	Puchengzhishezao	SRR10052869	9.9	119635800	15	China	Suanzao-64
SRR10052841	8	78203045	14	China	Suanzao-186	SRR10052952	9.9	114206483	16	China	Jinsi No.3
SRR13440780	8	66773391	12	China: Taigu\, Shanxi	Tengzhouluodihong	SRR10052584	9.91	108774068	15	China	Yuanling No.1
SRR10053048	8.01	67818502	12	China	Lianxianmuzao	SRR10053055	9.91	121799826	16	China	Wukuzhakexiaozao
SRR10052760	8.02	68270885	13	China	Taigujixinmi	SRR13440644	9.91	105030664	14	China: Taigu\, Shanxi	Jiaochengdongzao
SRR10052844	8.05	77349705	14	China	Suanzao-184	SRR10052563	9.92	116900812	16	China	Wuxiangyazao
SRR13440573	8.05	60291594	11	China: Taigu\, Shanxi	Songxiandazao	SRR10052607	9.92	115553161	16	China	Suanzao-170
SRR13440743	8.06	69563917	12	China: Taigu\, Shanxi	Dunhuangdazao	SRR10052795	9.92	109572170	15	China	Dalixiaoxiaodundunzao
SRR10052829	8.07	83650789	16	China	Suanzao-197	SRR10052713	9.93	117263082	17	China	Tianjingagazao
SRR13440730	8.07	66927322	11	China: Taigu\, Shanxi	Lianxiankulianzao	SRR10053070	9.93	117691128	16	China	Yiwudazao

SRR10052758	8.08	70328945	13	China	Jishanyuanzao	SRR10053105	9.93	104507081	14	China	Suanzao-207
SRR10052849	8.08	77763806	13	China	Suanzao-179	SRR13440759	9.93	101291527	14	China: Taigu\, Shanxi	Xupumuzao
SRR10052833	8.1	72838698	13	China	Suanzao-194	SRR10052562	9.94	122545869	16	China	Linyiyuanlingzao
SRR13440714	8.13	66534845	11	China: Taigu\, Shanxi	Yanchuantiaozao	SRR10052900	9.94	113471603	17	China	Xupuyuanzao
SRR10052872	8.14	76420053	13	China	Suanzao-61	SRR10052992	9.94	109758623	14	China	Lelingxiaozao
SRR10052930	8.14	73109329	13	China	Zhishegedazao	SRR10053027	9.94	112266239	16	China	Xinzhengjidanzao
SRR10052664	8.18	72018688	13	China	Suanzao-119	SRR10052578	9.95	122077437	17	China	Taiyuanqiutuanzao
SRR13440720	8.18	58827856	11	China: Taigu\, Shanxi	Linyijidanzao	SRR10052747	9.95	123437395	16	China	Jiaxianmidiancuimuzao
SRR13440731	8.21	68289532	12	China: Taigu\, Shanxi	Xishuangbannaxiaozao	SRR10052933	9.96	124793742	16	China	Heyanglinglingzao
SRR13440762	8.22	69285342	12	China: Taigu\, Shanxi	Xupumizao	SRR13440717	9.96	117074007	16	China: Taigu\, Shanxi	Beijingyingluozao
SRR10052912	8.25	75255457	14	China	Xuputangzao	SRR10052626	9.97	107475540	14	China	Suanzao-153
SRR13440775	8.25	70057733	12	China: Taigu\, Shanxi	Xiangshanxiaobaizao	SRR13440678	9.97	110002918	14	China: Qingjian\, Shaanxi	Xingtai No.18
SRR13440690	8.27	69307699	12	China: Taigu\, Shanxi	Yanchuanlaoyasuanzao	SRR10052617	9.98	113368442	15	China	Suanzao-161
SRR13440707	8.28	71283482	12	China: Qingjian\, Shaanxi	Fengmiguan	SRR10052985	9.98	115206015	15	China	Xinzhengdazao
SRR13440724	8.28	65903938	11	China: Taigu\, Shanxi	Wuxiangyazao	SRR10052997	9.98	114436604	15	China	Dalizhizao
SRR13440756	8.28	67877530	12	China: Taigu\, Shanxi	Shexianmazao	SRR10052729	9.99	122584896	16	China	Kongfusucuizao
SRR10052704	8.29	80343430	13	China	Suanzao-83	SRR10052780	9.99	115349384	16	China	Linfentuanzao-1

SRR13440600	8.3	65999771	11	China: Taigu\, Shanxi	Puyangsanbianhong	SRR10052899	9.99	120478811	16	China	Xupubaopizao
SRR13440578	8.32	68359660	12	China: Qingjian\, Shaanxi	Dalilinqinzao	SRR10052969	9.99	110425558	16	China	Lingbaodazao
SRR10052847	8.33	91702999	15	China	Suanzao-181	SRR10052973	9.99	111916496	15	South Korea	Yuechu
SRR10053011	8.33	73084724	13	China	Jisixiaozao	SRR10052993	9.99	115992794	16	China	Tenghzouchanghong
SRR13440584	8.35	71134061	12	China: Taigu\, Shanxi	Lelingchangmuzao	SRR10053058	9.99	118745177	16	China	Linzexiaozao
SRR13440604	8.36	63892327	11	China: Taigu\, Shanxi	Xianxianyuanxiaozao	SRR13440729	9.99	109053352	14	China: Taigu\, Shanxi	Gendedazao
SRR10052835	8.38	89096917	16	China	Suanzao-192	SRR10052558	10	120557453	16	China	Linfenmugeda
SRR13440583	8.38	73167782	12	China: Taigu\, Shanxi	Zaozhuanghuluchanghong	SRR10052588	10	111450862	15	China	Dalilongzao
SRR10053042	8.39	79437207	13	China	Guangdongzhenzhuzao	SRR10052624	10	111253375	15	China	Suanzao-222
SRR13440746	8.39	73985850	12	China: Taigu\, Shanxi	Linzexiaozao	SRR10052658	10	110791561	15	China	Suanzao-219
SRR13440753	8.42	73979517	12	China: Taigu\, Shanxi	Zhongningxiaoyuanzao	SRR10052732	10	113753246	15	China	Suanzao-248
SRR13440773	8.43	70677503	12	China: Taigu\, Shanxi	Miyunxiaozao	SRR10052734	10	119236658	15	China	Lelingmayizao
SRR10052828	8.44	85620218	15	China	Suanzao-198	SRR10052783	10	120862603	18	China	Pingyaobuluosu
SRR10053103	8.44	81522034	13	China	Suanzao-6	SRR10052808	10	114460999	16	China	Yuanquzao
SRR10052684	8.45	79729988	13	China	Suanzao-101	SRR10052809	10	116868460	16	China	Jiaochengtiansuanzao
SRR13440667	8.45	69554413	12	China: Qingjian\, Shaanxi	Xingtai No.35	SRR10052862	10	126275056	17	China	Suanzao-70
SRR13440764	8.46	70956378	12	China: Taigu\, Shanxi	Xuputiansuanzao	SRR10052892	10	110414894	15	China	Suanzao-201
SRR13440688	8.47	71762146	12	China:	Qingjianlaoya	SRR10052922	10	117073030	16	China	Xupuguany

				Taigu\, Shanxi	suanzao						inzao
SRR13440704	8.47	78747013	13	China: Qingjian\, Shaanxi	Jing60	SRR10052937	10	115670653	17	China	Huluchanghong
SRR10052834	8.48	86705370	15	China	Suanzao-193	SRR10052955	10	115571734	15	China	Hejintiaozao
SRR10052850	8.5	88199323	14	China	Suanzao-178	SRR10052965	10	113106057	17	China	Suanzao-240
SRR10053046	8.5	74514394	13	China	Xishuangxiaozao	SRR10052981	10	112188817	15	China	Xianxian No.21
SRR13440669	8.5	72697321	12	China: Qingjian\, Shaanxi	Xingtai No.30	SRR10052990	10	116885022	17	China	Zhongcaobenzao
SRR10052832	8.51	67719692	12	China	Suanzao-226	SRR10053001	10	112511723	15	China	Binxianyuanzao
SRR13440781	8.51	72520795	12	China: Qingjian\, Shaanxi	Xiajinchahuzao	SRR13440587	10	109685994	14	China: Taigu\, Shanxi	Lelingwuhexiaozao
SRR10052614	8.55	83243895	14	China	Suanzao-164	SRR13440625	10	117208455	16	China: Taigu\, Shanxi	Hongzhaocuizao
SRR10052775	8.56	79912307	14	China	Hongzhaocuizao	SRR13440639	10	111506111	14	China: Taigu\, Shanxi	Taigulinglingzao
SRR10052575	8.57	79715435	13	China	Taiyuanshiyuehong	SRR13440648	10	104769452	14	China: Qingjian\, Shaanxi	Zanhuangdazao
SRR13440581	8.57	70548048	12	China: Taigu\, Shanxi	Taigudundunzao	SRR13440654	10	114228081	15	China: Taigu\, Shanxi	Xiangfenyazao
SRR10052703	8.59	78731361	13	China	Suanzao-84	SRR13440679	10	120601607	15	China: Qingjian\, Shaanxi	Xingtai No.19
SRR10052998	8.59	67091342	12	China	Suanzao-237	SRR13440691	10	115470254	15	China: Taigu\, Shanxi	Jiaxianguodongxingsuanzao
SRR13440778	8.59	73114803	12	China: Taigu\, Shanxi	Zanhuangchangzao	SRR13440776	10	106519952	14	China: Taigu\, Shanxi	Qingyunxiaolizao
SRR10052757	8.6	77893255	14	China	Jishanbanzao	SRR10052560	10.1	119909485	16	China	Suanzao-243
SRR10052762	8.6	70524870	13	China	Baodexiaozao	SRR10052589	10.1	114982376	15	China	Dalilinglingzao

SRR10052931	8.6	77561007	13	China	Jiaxianyazao	SRR10052628	10.1	116828696	15	China	Suanzao-151
SRR13440599	8.6	72650734	12	China: Qingjian\, Shaanxi	Lingbaodazao	SRR10052685	10.1	121456531	16	China	Suanzao-100
SRR13440623	8.6	66684618	11	China: Taigu\, Shanxi	Xinzhengqitoubai	SRR10052698	10.1	113481120	16	China	Suanzao-88
SRR13440725	8.6	78878963	13	China: Taigu\, Shanxi	Dingxiangxiaozao	SRR10052719	10.1	120361514	16	China	Chuanganzao
SRR10052707	8.62	92472486	14	China	Suanzao-80	SRR10052730	10.1	118464900	16	China	Dong'edaguazao
SRR10052804	8.62	77614389	13	China	Xiaxianziyuanzao	SRR10052740	10.1	127716891	16	China	Hetaowen-2
SRR10053020	8.62	86975169	14	China	Suanzao-235	SRR10052796	10.1	121510788	16	China	Xinzhengdamaya
SRR13440734	8.62	73188400	12	China: Taigu\, Shanxi	Zanxindazao	SRR10052800	10.1	119174081	15	China	Chuanlingzao
SRR13440576	8.63	74689814	12	China: Taigu\, Shanxi	Jiaxianyazao	SRR10052814	10.1	115153251	15	China	Qingxuyuanzao
SRR10052766	8.64	76688330	13	China	Miyunxiaozao	SRR10052820	10.1	120111144	16	China	Pingshunjunzao
SRR10052947	8.65	78160059	13	China	Tengzhoudamaya	SRR10052853	10.1	129740111	17	China	Suanzao-175
SRR13440747	8.65	72297352	12	China: Taigu\, Shanxi	Guanyangdongzao	SRR10052874	10.1	131949627	18	China	Suanzao-59
SRR10052821	8.66	71059004	12	China	Suanzao-227	SRR10052883	10.1	125776488	17	China	Suanzao-51
SRR10052665	8.67	82771749	13	China	Suanzao-118	SRR10052889	10.1	118557587	15	China	Suanzao-45
SRR10053057	8.69	85552701	14	China	Gansudiaodiaopo	SRR10052896	10.1	118151540	16	China	Nanjingmutouzao
SRR10052663	8.7	84571679	14	China	Suanzao-120	SRR10052909	10.1	117348027	17	China	Xupuyuansuanzao
SRR10052830	8.71	89080718	15	China	Suanzao-196	SRR10052927	10.1	114305299	15	China	Tianjinminzao
SRR10052885	8.71	88427387	13	China	Suanzao-49	SRR10052928	10.1	126569531	17	China	Beijingjidanzao
SRR10052652	8.73	82020505	13	China	Suanzao-130	SRR10052932	10.1	126126413	17	China	Jiaxianxiyaozao
SRR10052986	8.73	79404480	13	China	Puchengdundunzao	SRR10052935	10.1	118391853	16	China	Jiaxianbaizao

SRR13440700	8.73	70763108	12	China: Qingjian\, Shaanxi	Yanliangcuizao	SRR10052974	10.1	112763348	15	China	Xianxianmianzao
SRR10052616	8.74	87385312	13	China	Suanzao-162	SRR10052988	10.1	117144189	15	China	Dalibachizao
SRR13440582	8.74	75362247	12	China: Taigu\, Shanxi	Tengzhoudamaya	SRR10053005	10.1	111988789	15	China	Xinzhengqitoubai
SRR13440774	8.75	83850691	13	China: Taigu\, Shanxi	Beijingjidanzao	SRR13440589	10.1	104079095	14	China: Taigu\, Shanxi	Daligedazao
SRR10052638	8.76	84103303	13	China	Suanzao-143	SRR13440615	10.1	116586020	15	China: Taigu\, Shanxi	Daliganweiba
SRR10052812	8.77	86295308	14	China	Suanzao-231	SRR13440638	10.1	118840860	15	China: Taigu\, Shanxi	Cangxiantunzizao
SRR10052667	8.78	82569998	14	China	Suanzao-116	SRR13440655	10.1	117565085	15	China: Qingjian\, Shaanxi	Jishanbanzao
SRR10052773	8.78	75870339	13	China	Yunchengxiangzao	SRR13440716	10.1	111421970	15	China: Taigu\, Shanxi	Lichengdamazao
SRR10053053	8.8	80982564	13	China	Kuerlexiaozao	SRR13440749	10.1	117243666	15	China: Taigu\, Shanxi	Beijingpaopaozaohong
SRR10052913	8.81	87905249	14	China	Xuputiansuanzao	SRR10052561	10.2	124729099	17	China	Linyijidanzao
SRR10052940	8.81	79018396	13	China	Dabailing	SRR10052568	10.2	129279789	16	China	Wuxiangtianzao
SRR10052941	8.81	80231715	13	China	Yanzhousanbianse	SRR10052606	10.2	122082742	16	China	Suanzao-171
SRR10052944	8.81	82931349	13	China	Lelingchangmuzao	SRR10052687	10.2	131825233	18	China	Suanzao-98
SRR10052906	8.82	81355633	13	China	Xupumizao	SRR10052690	10.2	133766570	17	China	Suanzao-95
SRR10052803	8.83	89303054	14	China	Taigushenglizao	SRR10052745	10.2	122975660	16	China	Dalixiaoyuanzao
SRR10052571	8.84	86077601	14	China	Suanzao-242	SRR10052818	10.2	122833162	17	China	Pinglujianzao
SRR13440750	8.85	76162573	12	China: Taigu\, Shanxi	Nanjingyazao	SRR10052819	10.2	123138882	17	China	Lvliangmuzao
SRR13440767	8.85	78493873	12	China: Taigu\,	Hengyangzhenzhuzao	SRR10052824	10.2	115985636	16	China	Yucituanzao

				Shanxi							
SRR13440772	8.85	86758696	14	China: Taigu\, Shanxi	Beijinglangjiayuanzao	SRR10052907	10.2	122402630	17	China	Linyizhenzhulongzao
SRR13440684	8.87	84837839	13	China: Qingjian\, Shaanxi	Xingtai No.13	SRR10052910	10.2	123873642	16	China	Xupujidanzao
SRR10052915	8.88	80199295	12	China	Suanzao-206	SRR10052991	10.2	112990277	14	China	Lelingwuhexiaozao
SRR10052678	8.89	90275971	15	China	Suanzao-106	SRR10053000	10.2	118918477	15	China	Malianxiaozao
SRR10052958	8.89	84193929	13	China	Linyibenzao	SRR10053002	10.2	117302268	15	China	Puchengyuanlizao
SRR13440601	8.89	83080540	13	China: Qingjian\, Shaanxi	Xinzhenghuizao	SRR10053003	10.2	118929229	16	China	Puchengmianzao
SRR10053038	8.9	83701088	14	China	Xinguang	SRR10053012	10.2	115998165	16	China	Xianxiansuanzao
SRR10052564	8.91	88246503	14	China	Shanxidalingzao	SRR10053015	10.2	119736937	18	China	Shulutangzao
SRR10052673	8.91	80696931	13	China	Suanzao-111	SRR10053021	10.2	121489903	17	China	Xianxianlajiaozao
SRR13440617	8.91	80599479	13	China: Taigu\, Shanxi	Zaoqiangmalianxiaozao	SRR10053052	10.2	126302268	16	China	Xinjiangchangyuanzao
SRR10052763	8.92	73075083	12	China	Suanzao-229	SRR10053076	10.2	129046865	17	China	Suanzao-31
SRR10052976	8.92	80822167	13	China	Suanzao-239	SRR10053097	10.2	130839576	17	China	Suanzao-12
SRR13440715	8.92	84545145	13	China: Taigu\, Shanxi	Fulingjidanzao	SRR13440620	10.2	119332095	15	China: Taigu\, Shanxi	Dalijidanzao
SRR10052771	8.93	79480804	14	China	Taiguhupingzao	SRR13440641	10.2	119200727	15	China: Taigu\, Shanxi	Linfentuanzao
SRR10052977	8.93	84942458	14	China	Shenxianchuanganhong	SRR13440671	10.2	122973774	16	China: Taigu\, Shanxi	Jishanyuanzao
SRR10052759	8.94	86700640	14	China	Xiangfenyuanzao	SRR10052579	10.3	134503022	17	China	Linyilajiaozao
SRR10052880	8.94	88721638	13	China	Suanzao-54	SRR10052581	10.3	128883115	17	China	Yuanlingzao
SRR10052959	8.94	81765106	13	China	Shanxihabazao	SRR10052620	10.3	120204026	15	China	Suanzao-158
SRR13440611	8.94	82074394	13	China: Qingjian\,	Lelingmopanzao	SRR10052625	10.3	124037805	15	China	Suanzao-154

SRR13440663	8.94	84393666	13	Shaanxi China: Taigu\, Shanxi	Xingtai No.36	SRR10052630	10.3	111992439	15	China	Suanzao-149
SRR13440672	8.94	78631233	12	China: Taigu\, Shanxi	Taigudongzizao	SRR10052631	10.3	127962246	16	China	Suanzao-148
SRR10052831	8.95	105124090	16	China	Suanzao-195	SRR10052644	10.3	116423607	15	China	Suanzao-137
SRR10052903	8.95	82059758	13	China	Suanzao-252	SRR10052653	10.3	118700871	15	China	Suanzao-129
SRR10052946	8.95	91333790	14	China	Tai'ansuyuanling	SRR10052677	10.3	126252927	17	China	Suanzao-107
SRR10052577	8.96	87435851	14	China	Dingxiangxiaozao	SRR10052727	10.3	132931391	18	China	Shibingzao
SRR13440629	8.96	81608776	13	China: Taigu\, Shanxi	Tongbaidazao	SRR10052737	10.3	128327479	16	China	Lingbaoling No.1
SRR10052995	8.98	83895192	13	China	Xiyingbenzao	SRR10052741	10.3	128213027	16	China	Puyangxiaozao
SRR10052752	8.99	75439564	12	China	Suanzao-230	SRR10052753	10.3	122876747	17	China	Xiangfenmuzao
SRR10052826	9	102429066	16	China	Suanzao-200	SRR10052781	10.3	131220891	18	China	Linfenmizao
SRR10052765	9.01	88256573	14	China	Beijingbenzao	SRR10052797	10.3	131403534	16	China	Xinzhengchangjixin
SRR13440677	9.01	83196782	13	China: Qingjian\, Shaanxi	Xingtai No.18_2	SRR10052806	10.3	135115778	18	China	Linfentuanzao-2
SRR10052716	9.02	95462760	16	China	Xiangshanxiaobaizao	SRR10052846	10.3	133479584	17	China	Suanzao-182
SRR10053028	9.02	83303050	14	China	Dongzao	SRR10052859	10.3	140631503	19	China	Suanzao-73
SRR10053049	9.02	87965506	14	China	Lianxiantangzao	SRR10052861	10.3	134780787	17	China	Suanzao-71
SRR13440580	9.02	84089169	13	China: Taigu\, Shanxi	Ningyangxuanlingzao	SRR10052878	10.3	125319493	16	China	Suanzao-55
SRR10052951	9.03	87232167	13	China	Ningyangxuanlingzao	SRR10052886	10.3	128294194	16	China	Suanzao-48
SRR13440779	9.03	83547890	13	China: Taigu\, Shanxi	Beibeixiaozao	SRR10052902	10.3	128721875	16	China	Xupushatangzao
SRR13440784	9.03	84189377	13	China: Qingjian\,	Linyilizao	SRR10052925	10.3	129958442	17	China	Tianjinerqiuzao

SRR10052583	9.04	85102111	13	Shaanxi China	Suanzao-205	SRR10052936	10.3	128256103	17	China	Yangnaizao
SRR10052613	9.04	91938590	14	China	Suanzao-223	SRR10052970	10.3	119259788	15	China	Zaoqiangjunzao
SRR10052651	9.04	82892535	13	China	Suanzao-131	SRR10052971	10.3	129492048	18	China	Sanbianhong
SRR10052761	9.05	83236172	14	China	Taiguhupingsuan	SRR10052987	10.3	122213310	16	China	Suanzao-238
SRR10052786	9.05	79440310	13	China	Puchengzhishezao	SRR10053016	10.3	126294184	18	China	Cangxianxiaozao
SRR10052670	9.06	91408988	14	China	Suanzao-114	SRR10053029	10.3	125845794	17	China	Linxianwutouzao
SRR13440652	9.06	83186067	13	China: Taigu\, Shanxi	Jiaochengjunzao	SRR10053036	10.3	128064701	16	China	Binxianheigeda
SRR10052567	9.07	85722460	13	China	Wuxiangtanzao	SRR10053062	10.3	126270940	16	China	Guanyangchangzao
SRR13440727	9.07	86110918	13	China: Taigu\, Shanxi	Baodexiaozao	SRR10053099	10.3	123584643	16	China	Suanzao-10
SRR10052755	9.08	89175157	15	China	Hongzhaoshiyuehong	SRR13440593	10.3	127217134	15	China: Taigu\, Shanxi	Binxianyuanzao
SRR10053041	9.08	85500508	14	China	Suanzao-257	SRR13440619	10.3	117597899	15	China: Taigu\, Shanxi	Puchengmianzao
SRR13440757	9.08	90143081	13	China: Taigu\, Shanxi	Yiwudazao	SRR13440636	10.3	131488130	16	China: Taigu\, Shanxi	Yongjihamazao
SRR13440596	9.09	86944178	13	China: Taigu\, Shanxi	Neihuangbianhesuan	SRR13440640	10.3	128624827	16	China: Taigu\, Shanxi	Xiaxianziyuanzao
SRR13440628	9.09	79697384	12	China: Qingjian\, Shaanxi	Shandonglizao	SRR13440642	10.3	128040395	15	China: Taigu\, Shanxi	Jishanchangzao
SRR10052724	9.1	92189103	14	China	Tengzhouluodihong	SRR13440662	10.3	123138830	17	China: Qingjian\, Shaanxi	Xingtai No.37
SRR10052662	9.11	83023519	13	China	Suanzao-121	SRR13440694	10.3	119338438	16	China: Taigu\, Shanxi	Taiguhupingsuan
SRR10052879	9.11	87657766	13	China	Suanzao-212	SRR13440703	10.3	131285786	16	China: Taigu\, Shanxi	Jianghuaiyihao

SRR10052712	9.12	104244866	15	China	Suanzao-75	SRR13440719	10.3	118443141	15	China: Taigu\, Shanxi	Xupushatangzao
SRR13440771	9.12	86002112	13	China: Taigu\, Shanxi	Lichengxiaozao	SRR13440738	10.3	122117855	17	China: Taigu\, Shanxi	Beijingbenzao
SRR10052709	9.13	98400611	15	China	Suanzao-78	SRR3081342	10.3	90567408	13	China:Shaanxi Province	Junzao
SRR10052911	9.13	88764390	15	China	Xupuhuluzao	SRR10052572	10.4	119862980	15	China	Taiyuanchangzao
SRR13440711	9.13	87677482	13	China: Qingjian\, Shaanxi	Qiyuexian	SRR10052603	10.4	138410384	19	China	Suanzao-35
SRR10052871	9.14	105480343	15	China	Suanzao-62	SRR10052733	10.4	137050174	18	China	Tai'anmalingcui
SRR10052881	9.14	93209986	14	China	Suanzao-53	SRR10052735	10.4	141020889	17	China	Shandonglajiaozao
SRR13440647	9.14	85023347	13	China: Taigu\, Shanxi	Linfenzhenhulu	SRR10052738	10.4	121342301	15	China	Guoxingpingguozhuang
SRR13440676	9.14	86749528	13	China: Qingjian\, Shaanxi	Xingtai No.27	SRR10052744	10.4	139063413	17	China	Xinzhengjiuyueqing
SRR10052661	9.15	95697272	14	China	Suanzao-122	SRR10052746	10.4	139940092	18	China	Puchengjinzao
SRR13440768	9.15	83132210	12	China: Taigu\, Shanxi	Xuputangzao	SRR10052799	10.4	134477282	18	China	Pozao
SRR10053087	9.16	96601061	14	China	Suanzao-21	SRR10052866	10.4	132176058	17	China	Suanzao-66
SRR13440605	9.16	77581362	12	China: Taigu\, Shanxi	Xianxianmianzao	SRR10053022	10.4	129445158	17	China	Xianxianxiaoxiaozao
SRR13440683	9.16	87147756	13	China: Taigu\, Shanxi	Xiangfenyuanzao	SRR10053033	10.4	136914908	18	China	Yongchengyuanhong
SRR10052632	9.17	95935844	14	China	Suanzao-147	SRR10053034	10.4	126577178	18	China	Dalijidanzao
SRR10052825	9.17	89918546	14	China	Linyilizao	SRR10053037	10.4	130145451	16	China	Suanzao-1
SRR10052864	9.17	99553132	15	China	Suanzao-68	SRR10053104	10.4	133521768	17	China	Suanzao-5
SRR10052627	9.18	87341053	13	China	Suanzao-152	SRR13440608	10.4	131221113	16	China: Taigu\, Shanxi	Zanhuangchangzao_2
SRR10052682	9.18	94630676	14	China	Suanzao-103	SRR13440612	10.4	116219164	15	China:	Yanchuanb

										Taigu\, Shanxi	aizao
SRR10052683	9.18	91459396	14	China	Suanzao-102	SRR13440622	10.4	122864081	15	China: Qingjian\, Shaanxi	Guantan No.11
SRR10052948	9.18	91060509	14	China	Tai'andacuizao	SRR13440633	10.4	128972468	17	China: Taigu\, Shanxi	Shenxianchuanganzao
SRR13440653	9.19	84595442	13	China: Taigu\, Shanxi	Hongzhaoshiyuehong	SRR13440736	10.4	122165765	16	China: Taigu\, Shanxi	Kudonglexiaozao
SRR13440710	9.19	90685470	13	China: Qingjian\, Shaanxi	Jiaxianchangzao	SRR10052640	10.5	145807147	18	China	Suanzao-141
SRR10052770	9.2	88399274	14	China	Taigulangzao	SRR10052647	10.5	130550035	16	China	Suanzao-220
SRR10052856	9.2	98472668	14	China	Suanzao-173	SRR10052676	10.5	142664695	18	China	Suanzao-108
SRR10052982	9.2	92340094	15	China	Xianxianyuanxiaozao	SRR10052731	10.5	145893221	19	China	Qufuhoutouzao
SRR10052999	9.2	88355089	14	China	Binxiansuangeda	SRR10052748	10.5	129212864	16	China	Yanchuangoutouzao
SRR13440751	9.2	91018939	13	China: Taigu\, Shanxi	Nanjingzao	SRR10052776	10.5	138016108	19	China	Yunchengpopozao
SRR10052634	9.22	86734213	13	China	Suanzao-145	SRR10052817	10.5	131101259	18	China	Qixianlongzao
SRR10052873	9.22	103708528	15	China	Suanzao-60	SRR10052890	10.5	132357040	17	China	Suanzao-211
SRR10052749	9.23	87845667	14	China	Pingyaokuduanzao	SRR10053024	10.5	128427439	18	China	Shenxianchuanganzao
SRR10052845	9.23	100721015	15	China	Suanzao-183	SRR10053040	10.5	134125853	16	China	Suanzao-258
SRR10053019	9.23	81797281	13	China	Xianxianmuzao	SRR10053065	10.5	146794741	18	China	Hubeipopozao
SRR13440695	9.23	84823821	13	China: Qingjian\, Shaanxi	Qingjiansuanzao	SRR10053075	10.5	150118496	20	China	Suanzao-32
SRR3081351	9.24	68340439	11	China:Shaanxi Province	Junzao	SRR10053091	10.5	140050240	17	China	Suanzao-17
SRR10052787	9.25	95488079	14	China	Daliganweiba	SRR13440574	10.5	131190154	16	China: Taigu\, Shanxi	Puyangxiaozao

SRR10052916	9.25	92005526	14	China	Ruchengzao	SRR13440613	10.5	121655616	15	China: Taigu\, Shanxi	Yanchuanniunaicuizao
SRR10053043	9.25	103588123	15	China	Lianxiankulianzao	SRR13440621	10.5	116932124	14	China: Taigu\, Shanxi	Dalixiaododongdong
SRR13440755	9.26	93009088	14	China: Taigu\, Shanxi	Langxiniunaizao	SRR13440635	10.5	129319918	15	China: Qingjian\, Shaanxi	Xuechengdongzao
SRR10052604	9.27	85675607	13	China	Suanzao-210	SRR10052637	10.6	120811954	16	China	Suanzao-144
SRR13440692	9.27	94411125	14	China: Qingjian\, Shaanxi	Tiansuanzao	SRR10052693	10.6	139451777	19	China	Suanzao-93
SRR10053004	9.28	89520181	14	China	Yanchuantiaozao	SRR10052788	10.6	129071665	16	China	Yanchuanbaizao
SRR10052774	9.29	87668935	13	China	Suanzao-228	SRR10052798	10.6	142489630	19	China	Qingyuandadanzao
SRR10052854	9.29	83924141	13	China	Suanzao-224	SRR10052801	10.6	137011280	17	China	Suanzao-232
SRR3081340	9.29	71328805	12	China:Shaanxi Province	Junzao	SRR10052822	10.6	134613532	17	China	Taiguhuluzao
SRR10052711	9.3	97100290	14	China	Suanzao-76	SRR10052839	10.6	150880675	20	China	Suanzao-188
SRR10052671	9.31	99209542	14	China	Suanzao-113	SRR10052840	10.6	138861908	17	China	Suanzao-187
SRR10052851	9.31	111512299	17	China	Suanzao-177	SRR10052865	10.6	136919185	17	China	Suanzao-67
SRR10052960	9.31	93885583	14	China	Pingluchengtuozao	SRR10052876	10.6	140186497	17	China	Suanzao-57
SRR13440748	9.31	88629318	13	China: Taigu\, Shanxi	Guanyangchangzao	SRR10052888	10.6	130035870	18	China	Suanzao-46
SRR10052609	9.32	95625605	13	China	Suanzao-168	SRR10052956	10.6	135844490	17	China	Suanzao-244
SRR10052857	9.32	92387509	13	China	Suanzao-214	SRR10053025	10.6	130029861	17	China	Gusuxiaozao
SRR13440572	9.32	88050630	13	China: Taigu\, Shanxi	Neihuangpingguozao	SRR10053030	10.6	134130759	18	China	Zhenpingtailihong
SRR10052636	9.33	90882961	13	China	Suanzao-203	SRR10053050	10.6	133249697	18	China	Suanzao-255
SRR10052696	9.33	94609987	27	China	Suanzao-90	SRR10053089	10.6	127228411	18	China	Suanzao-19
SRR13440675	9.33	95076018	13	China:	Xingtai No.26	SRR10053093	10.6	145482887	17	China	Suanzao-15

				Qingjian\, Shaanxi							
SRR10052691	9.34	93551463	14	China	Suanzao-216	SRR10053100	10.6	133587321	17	China	Suanzao-9
SRR10052843	9.34	88597498	13	China	Suanzao-225	SRR13440631	10.6	136685158	16	China: Taigu\, Shanxi	Xianxiansuanzao
SRR10052917	9.34	93939889	15	China	Xupujianzao	SRR13440634	10.6	142704276	17	China: Taigu\, Shanxi	Zaoqiangpozao
SRR13440590	9.34	90964087	13	China: Taigu\, Shanxi	Binxiansuangedazao	SRR13440777	10.6	141831272	17	China: Taigu\, Shanxi	Baodingbanzao
SRR10052858	9.35	101882209	15	China	Suanzao-74	SRR10052565	10.7	144820188	19	China	Yucimaohouzao
SRR13440718	9.35	84403885	13	China: Taigu\, Shanxi	Xupujidan	SRR10052629	10.7	132436332	16	China	Suanzao-150
SRR10052953	9.36	100431157	14	China	Linyimalingzao	SRR10052689	10.7	134147674	16	China	Suanzao-96
SRR13440760	9.36	93900730	13	China: Taigu\, Shanxi	Baodeyouzao	SRR10052697	10.7	134362736	17	China	Suanzao-89
SRR10052891	9.37	95152426	13	China	Suanzao-202	SRR10052705	10.7	138473981	17	China	Suanzao-82
SRR10052950	9.37	96074953	14	China	Jinsi No.2	SRR10052739	10.7	143191539	18	China	Shidingzao
SRR13440754	9.37	93170177	13	China: Taigu\, Shanxi	Xuanchengjianzao	SRR10052779	10.7	146105967	20	China	Linfenzhenhulu
SRR10052967	9.38	91700771	14	China	Guangyangzao	SRR10052792	10.7	142641145	18	China	Lelingmopanzao
SRR13440637	9.38	91989064	13	China: Taigu\, Shanxi	Xianxianmuzao	SRR13440765	10.7	131088458	16	China: Taigu\, Shanxi	Ruchengzao
SRR13440657	9.38	96424909	13	China: Qingjian\, Shaanxi	Xingtai No.42	SRR10052586	10.8	154623937	20	China	Fengmiguan
SRR13440697	9.38	90515531	13	China: Qingjian\, Shaanxi	Xingtai No.6	SRR10052595	10.8	152553226	18	China	Suanzao-43
SRR13440705	9.38	96421534	14	China: Taigu\, Shanxi	Pingyaodazao	SRR10052621	10.8	142304860	18	China	Suanzao-157
SRR10052894	9.39	100139140	14	China	Fuyangmayizao	SRR10052654	10.8	157801065	19	China	Suanzao-128
SRR10052619	9.4	97047103	13	China	Suanzao-159	SRR10052686	10.8	140789785	17	China	Suanzao-99

SRR10053032	9.4	95205817	14	China	Yongchengchanghong	SRR10052855	10.8	146172165	18	China	Suanzao-174
SRR10053072	9.4	99784247	14	China	Suanzao-253	SRR10052887	10.8	139801803	18	China	Suanzao-47
SRR10052570	9.41	94132068	14	China	Yucijiuyueqing	SRR10053088	10.8	148438424	20	China	Suanzao-20
SRR10052921	9.41	98919970	14	China	Kangtouzao	SRR10053106	10.8	145536380	18	China	Suanzao-4
SRR10052972	9.41	91883360	15	South Korea	Wudeng	SRR13440643	10.8	152919522	19	China: Taigu\, Shanxi	Jiaochengtiansuanzao
SRR10053051	9.41	99445346	16	China	Zunyitianzao	SRR10052597	10.9	154365609	20	China	Suanzao-41
SRR13440591	9.41	93387874	13	China: Taigu\, Shanxi	Binxianjinzao	SRR10053039	10.9	150921338	20	China	Suanzao-256
SRR13440732	9.41	89484682	13	China: Taigu\, Shanxi	Zunyitianzao	SRR10053077	10.9	153837729	20	China	Suanzao-30
SRR10052599	9.42	108904286	15	China	Suanzao-39	SRR10053082	10.9	142488967	18	China	Suanzao-25
SRR10052610	9.42	99738754	14	China	Suanzao-167	SRR10052600	11	163079816	19	China	Suanzao-38
SRR10052767	9.43	102808417	15	China	Beijingpaopaozao	SRR10052601	11	154649922	18	China	Suanzao-37
SRR10053074	9.43	94484052	14	China	Suanzao-33	SRR10052612	11	148643112	18	China	Suanzao-165
SRR13440660	9.43	86777624	13	China: Taigu\, Shanxi	Hongzhaohuluzao	SRR10052641	11	156968280	20	China	Suanzao-140
SRR10052576	9.44	94609347	14	China	Dingxiangshanzao	SRR10052643	11	162321269	19	China	Suanzao-138
SRR10052785	9.44	93811591	14	China	Lintongguluzao	SRR10052645	11	161069910	20	China	Suanzao-136
SRR10053096	9.44	101555377	15	China	Suanzao-13	SRR10052672	11	149342367	19	China	Suanzao-112
SRR13440666	9.44	92735237	13	China: Qingjian\, Shaanxi	Xingtai No.34	SRR10053102	11	162027613	19	China	Suanzao-7
SRR10052914	9.46	95727116	14	China	Suanzao-251	SRR10053107	11	166618202	20	China	Suanzao-3
SRR10052923	9.46	107300720	15	China	Langjiayuanzao	SRR13440592	11	156047739	18	China: Taigu\, Shanxi	Xiangfenguantanzao
SRR10052994	9.46	99340728	15	China	Shaanximianzao	SRR13440645	11	158729933	18	China: Taigu\, Shanxi	Pingshunbenzao
SRR10052725	9.47	98451525	14	China	Beibeixiaozao	SRR10052557	11.1	168448801	20	China	Suanzao-2
SRR10053056	9.47	99271877	15	China	Kashigaerxiaozao	SRR10052648	11.1	156914009	19	China	Suanzao-134

SRR13440627	9.47	87691266	13	China: Qingjian\, Shaanxi	Huanghuadongzao	SRR10052659	11.1	161469852	19	China	Suanzao-124
SRR10052715	9.48	104228224	16	China	Yingluozao	SRR10052789	11.1	157703705	21	China	Shaanxidabaizao
SRR10052764	9.48	97178194	15	China	Beijingzhuizibai	SRR10053092	11.1	166554842	20	China	Suanzao-16
SRR10052875	9.48	107535563	16	China	Suanzao-58	SRR13440673	11.1	157897674	20	China: Qingjian\, Shaanxi	Xingtai No.31
SRR10052966	9.48	98865382	16	China	Anyangtuanzao	SRR10052596	11.2	169411645	23	China	Suanzao-42
SRR13440575	9.48	98249231	14	China: Taigu\, Shanxi	Puchengjinzao	SRR10052605	11.2	158701394	18	China	Suanzao-172
SRR13440783	9.48	96319038	14	China: Taigu\, Shanxi	Yucituanzao	SRR10052618	11.2	157093333	20	China	Suanzao-160
SRR10052559	9.49	101834066	14	China	Hejinshuizao	SRR10052657	11.2	162849848	19	China	Suanzao-125
SRR10052722	9.49	101548245	14	China	Qingyunxiaolizao	SRR10052877	11.2	166567890	21	China	Suanzao-56
SRR10052742	9.49	105009749	15	China	Puyangsanbianchou	SRR10053084	11.2	158378894	21	China	Suanzao-24
SRR10052979	9.49	97436606	15	China	Hebeilongzao	SRR10053085	11.2	166192980	23	China	Suanzao-23
SRR10052815	9.5	109477009	15	China	Pingshunbenzao	SRR3095675	11.2	130918200	17	China: Taigu\, Shanxi	Dongzao
SRR10052926	9.5	90540583	13	China	Suanzao-250	SRR10052649	11.3	170380184	21	China	Suanzao-133
SRR10052975	9.5	99159596	14	China	Cangxianchangxiaozao	SRR10052655	11.3	160790957	20	China	Suanzao-127
SRR10053017	9.5	100304850	16	China	Cangxiantunzizao	SRR10052675	11.3	170804398	22	China	Suanzao-109
SRR13440708	9.5	95175522	14	China: Taigu\, Shanxi	Yanchuangoutouzao	SRR10052882	11.3	187049602	23	China	Suanzao-52
SRR13440722	9.5	93804542	13	China: Taigu\, Shanxi	Linyitiansuanzao	SRR10052884	11.3	164690110	23	China	Suanzao-50
SRR10052756	9.51	107489797	16	China	Xiangfenyazao	SRR10053073	11.3	164969976	21	China	Suanzao-34
SRR13440597	9.51	100220274	14	China: Taigu\,	Anyangtuanzao	SRR10052650	11.4	161280167	20	China	Suanzao-132

				Shanxi							
SRR13440606	9.51	93890387	13	China: Taigu\, Shanxi	Hetaowen	SRR10052666	11.4	164333857	22	China	Suanzao-117
SRR13440745	9.51	97023851	14	China: Taigu\, Shanxi	Linzedazao	SRR10052674	11.4	166453292	21	China	Suanzao-110
SRR10052751	9.52	110897345	17	China	Taigulinglingzao	SRR10052836	11.4	177882115	25	China	Suanzao-191
SRR10052964	9.52	100398121	14	China	Wanrongbolicui	SRR3095674	11.4	133670634	18	China:Taigu\, Shanxi Province	Dongzao
SRR10053069	9.52	99686830	14	China	Hubeilingdangzao	SRR3081206	11.5	127089100	17	China:Shaanxi Province	Junzao
SRR13440702	9.52	97845421	14	China: Qingjian\, Shaanxi	Lengbaiyu	SRR3081209	11.5	127012561	17	China:Shaanxi Province	Junzao
SRR13440649	9.53	101166389	14	China: Qingjian\, Shaanxi	Zhongyangmuzao	SRR3095680	11.5	150978609	24	China:Qingjian\, Shaanxi Province	Taigusuanzao
SRR13440696	9.53	93945276	13	China: Qingjian\, Shaanxi	Xingtai No.11	SRR10052681	11.6	175655794	23	China	Suanzao-104
SRR10052598	9.54	98021725	14	China	Suanzao-40	SRR10053080	11.6	175903262	26	China	Suanzao-27
SRR10053006	9.54	94865878	14	China	Xinzhengxiaoyuanzao	SRR3095688	11.7	151360826	22	China:Qingjian\, Shaanxi Province	Xingtaisuanzao_11
SRR10053010	9.54	107819337	16	China	huizao	SRR3095672	11.8	152587783	26	China:Taigu\, Shanxi Province	Gagazao
SRR10052569	9.55	98131725	14	China	Taiyuanlvfendan						

Supplementary table 2 Estimation values of genome heterozygosity and genome size of 296 jujube varieties based on 30X genome resequencing

Genotype name	Genome heterozygosity（%）	Genome size（bp）	Number of SNPs	Genotype name	Genome heterozygosity（%）	Genome size（bp）	Number of SNPs
Dongzao	1.3	409398207	5022176	Nangufenghuluzao	1.74	323431085	3770894
Neihuangpingguozao	1.31	378020524	5147859	Weihaijinsizao2	1.74	345806067	3423876

Yongjihamazao	1.35	378089409	3612885	Xiangzao	1.74	360182942	4035948
Zhongyangmuzao	1.37	388687037	3800114	Qingyuandadanzao	1.74	357546331	3762462
Xiangfenyuanzao	1.38	395909333	4000517	Huanghuadongzao	1.74	363122508	3978892
Beibeixiaozao	1.38	382840266	3780861	Zhenpingguangyangzao	1.74	360432717	3872681
Hongzhaocuizao	1.4	394552814	4004508	Shengxianbaipuzao	1.74	331195746	3621576
Taigumeimizao	1.4	378745765	3804479	Beijingjidanzao	1.74	350583150	3780741
Pingshunjunzao	1.41	379839093	3354337	Binxianyuanzao	1.75	331810676	3827649
Xinzhengxiaoyuanzao	1.42	373861783	3484565	Xiajinchahuzao	1.75	320908005	3760094
Sunanbaipuzao	1.43	361125543	3877561	Xupumizao	1.75	318104293	3900754
Xianxianxiaoxiaozao	1.44	377032600	3478569	Xupushatangzao	1.75	322377845	3958615
Lelingwuhexiaozao	1.44	365025708	3489625	Yunchengcuizao	1.75	340789846	4010320
Xuyiyanlaihongzao	1.44	360897759	3650138	Pingyaodazao	1.75	322346071	3750822
Nanjinglengzao	1.45	373287643	3631114	Tengzhoutangzao	1.75	323742800	3829904
Lichengxiaozao	1.46	372313097	3489964	Binxianjinzao	1.75	348163470	3736311
Xiangfenmuzao	1.46	384171021	4068609	Puyanghetaowenzao	1.75	346640465	3541411
Jinzandazao	1.48	323907015	4880811	Hengshanchangdazao	1.75	352863664	3859199
Xiangfenyazao	1.48	381433561	3797336	Xupubinglangzao	1.75	344573734	3531829
Zaoqianggutouxiaozao	1.48	358428119	3443265	Zhongningxiaoyuanzao	1.75	359641423	3674921
Cangxianchangxiaozao	1.49	348948727	3458744	Jinsizao1	1.76	316738250	3617668
Taiguduanzizao	1.49	369266570	3813512	Ruchengzao	1.76	312483323	3666077
Zaoqiangmalianxiaozao	1.49	359561576	3598363	Pozaozhibianzao1	1.76	329453667	3834560
Lintongguluzao	1.5	366113529	3567544	Hubeijixinzao	1.76	332534445	4082597
Chaoyangdapingdingzao	1.5	364442541	3779167	Jiaochengduanzao	1.76	358650062	4040196
Xianxianzao21	1.51	347701000	3484577	Xiaxianziyuanzao	1.76	361043361	4075142
Zanhuangchangzao	1.51	323089914	4934893	Zaoqiangcuizao	1.76	367804819	3687009
Daliyuanzao	1.51	384916982	3898050	Tengzhouchanghongzao	1.76	361677744	3851138
Xinzhengchangjixinzao	1.51	361425064	3709944	Nanjingyazao	1.76	354995086	3692330
Xupuyuanzao	1.51	366106098	3632439	Xiajinmamazao	1.77	328616998	3836764
Xinzhengqitoubaizao	1.53	365689210	3715391	Yucichangmuzao	1.77	325360439	3725144
Guanyangduanzao	1.53	363493339	3719601	Linfenmizao	1.77	345325349	3767375
Baodeyouzao	1.54	376351495	3789150	Linfentuanzao	1.77	354973215	3882811
Taigumeixinhongzao	1.54	371971590	3711981	Cangxianjinsixiaozao	1.77	347741926	3457051
Dayewuhezao	1.54	369135294	3387444	Xuechengdongzao	1.77	349945768	3743947
Shanxiqiyuexianzao	1.55	377191180	3809961	Qingyunxiaolizao	1.77	352451247	3761384
Xinzhengjiuyueqingzao	1.55	374271537	3931810	Fuyangmayizao	1.77	338506157	3738136
Aksuxiaozao	1.55	368041141	3829783	Tianjindamayazao	1.78	304687503	3654690
Huluchanghongzao	1.55	380309859	3912099	Linyimalingzao	1.78	318236488	3815843
Shexianmazao	1.55	371920835	3664029	Beijingzhuizibaizao	1.78	321117444	3763765
Chaoyangwanzao	1.56	333455049	3495625	Xiajindabailingzao	1.78	335720556	4041796
Cangxiantunzizao	1.56	349785923	3482276	Jishanbanzao	1.78	364245030	3896479
Miyunxiaozao	1.57	333579211	3494284	Linyilizao	1.78	371823261	4028295
Xupuchengchuizao	1.57	344340957	3290273	Yutianxiaozao	1.78	330627534	3428323
Pinglutuntunzao	1.57	358156786	3580867	Chaoyangdajiandingzao	1.78	352989913	3482124
Baodexiaozao	1.57	375522412	3766821	Shandonglajiaozao	1.79	330103761	3975160

Jiaochengtiansuanzao	1.57	376615974	4228536	Wanrongfuzao	1.79	334768787	4038406
Taiguhuluzao	1.58	369733425	3876596	Binxianheigadazao	1.79	360077705	3811611
Beijingpaopaozao	1.58	374383178	3915188	Zaoqiangshazao	1.79	354308380	3626042
Kunmingzao	1.58	364544080	3467784	Shenxianchuanganhongzao	1.8	313578401	3926498
Yucituanzao	1.59	372591117	3740777	Linyibobozao	1.8	323981220	4156487
Linfenzhenhuluzao	1.59	371953573	4135831	Yunchengpopozao	1.8	350631619	3962643
Taigudundunzao	1.59	370133811	3872118	Xiaxianyuancuizao	1.8	361240749	4181533
Xinzhengjidanzao	1.59	378926321	3620321	Dalixiaodundunzao	1.8	356933875	3889218
Jiaochengjunzao	1.6	359350982	3762778	Yanchuandabaizao	1.8	365872531	4000733
Beijingbenzao	1.6	351081395	3449106	Zhenpingtailihongzao	1.8	346070022	3640412
Pingshunbenzao	1.6	376152871	3747526	Shanximianzao	1.8	345475322	3604683
Hanguowudengzao	1.6	346720157	3355327	Dalilongzao	1.8	350542740	3847062
Puyangxiaozao	1.6	355628975	3448863	Hamidazao	1.8	357661008	3996268
Hebeizao13	1.61	333953571	3809832	Ningyangxuanlingzao	1.8	365226938	3768343
Taigumuzao	1.61	349546589	3759095	Xi'anyangnaizao	1.8	348638431	3452456
Beijingyingluozao	1.61	368226430	3901618	Shanxihuluzao	1.8	341071060	3374365
Xupuxiaoguosuanpanzao	1.62	327201273	3541109	kashengaerxiaozao	1.8	381483178	4277113
Taiguheiyezao	1.62	354945120	3665592	Minqinxiaozao	1.8	344702771	3655381
Taigulangzao	1.62	351013255	3766016	Xishuangbannaxiaozao	1.8	351936196	3756814
Jiaxianyazao	1.62	374131288	3769823	Puchengjinzao	1.81	331037104	3946295
Weihaijinsizao3	1.63	321903250	3475320	Xupuguanyinzao	1.81	306809016	3668437
Jinsizao4	1.63	325321114	3312384	Tengzhoudamazao	1.81	325476076	3804885
Shanxidalingzao	1.63	339649783	3771555	Shenxianchuanganzao	1.81	316129692	3893404
Xupubaopizao	1.63	341525889	3571243	Heyanglinglingzao	1.81	328200220	4098029
Pinglujianzao	1.63	360474168	3671700	Taiguhupingzao	1.81	312994113	3746248
Xincaidayuanfengzao	1.63	376798210	3992602	Chengwudongzao	1.81	358756890	3794212
Xinzhengdazao	1.64	335132012	3466543	Tai'anmalingcuizao	1.81	360972620	3777960
Xiyingbenzao	1.64	348552843	3919243	Ningyangdashibingzao	1.81	349907874	3532316
Tai'ansuyuanlingzao	1.64	333714188	3583416	Hunanchangzao	1.81	343758286	4013094
Dingxiangxiaozao	1.64	319509919	3469847	Wuxianshuituanzao	1.81	351364190	3778754
Yanchuanniunaicuizao	1.64	347329537	3938242	Xinzhengchangjixinzao	1.82	335646547	3957907
Yongchengyuanhongzao	1.64	360652095	4041670	Jishanyuanzao	1.82	359504734	4055089
Hanguoyuechuzao	1.65	317995595	3402330	Puyangtangzao	1.82	365231433	3972621
Xinledazao	1.65	339684422	3756328	Xuputiansuanzao	1.82	342256168	3914897
Jishanliuguanzao	1.65	366842456	4047970	Xuanchengjianzao	1.82	337670260	3575611
Daliganweibazao	1.65	358732465	3863638	Hubeiyuanzao	1.82	354462052	3834129
Puyangsanbianhongzao	1.65	366697897	3798106	Ningyangliuyuexianzao	1.83	307715359	3613586
Yangjiaozao	1.65	359456632	3749727	Taiyuanchangzao	1.83	311173411	3801190
Lelingchangmuzao	1.66	328935601	3415620	Linxianwutouzao	1.83	366153066	3924802
Fuyangmutouzao	1.66	326940273	3577072	Cangxianxiaozao	1.83	346932486	3395162
Dingxiangxingxingzao	1.66	367862432	3841428	Yanchuandieyazao	1.83	367286464	3695335
Gusuxiaozao	1.66	358314540	3429778	Lengbaiyuzao	1.83	347031888	3738263
Tianjinerqiuzao	1.67	320952067	3450632	Lanximazao	1.83	348251416	3559834

Xianxianyuanxiaozao	1.67	325710856	3436236	Hubeilingdangzao	1.83	342429879	3803088
Hongzhaoshiyuehongzao	1.67	370465092	4037796	Shandonglizao	1.83	342902498	3786614
Yuciyazao	1.67	382120441	4003053	Changxindianbaizao	1.84	293090772	3924607
Xinzhengdamayazao	1.67	355502345	3642433	Xupuchengtuozao	1.84	304141605	3530979
Shaoguanbaizao	1.67	362158371	3716027	Dalizhizao	1.84	321702372	3883799
Wutaimianzao	1.67	363083637	4031435	Shanxinaizao	1.84	347794905	3550334
Xuputangzao	1.68	317514632	3752889	Zhongcaobenzao	1.84	348248755	3840419
Lejinzao4	1.68	327823568	3455766	Xupumuzao	1.84	339430280	3579119
Yunnanzao3	1.68	322120350	3586546	Gendedazao	1.84	327930027	3620932
Kongfusucuizao	1.68	318903619	3658013	Dalilinglingzao	1.85	311213136	3840380
Wuxiangtianzao	1.68	361359045	4342068	Dong'edaguazao	1.85	316750240	4048390
Gansudiaodiaopozao	1.68	327234654	3728998	Xupudaguosuanpanzao	1.85	344423997	3580414
Xiangfenguantanzao	1.68	375825014	4181687	Langxiniunaizao	1.85	337750917	3594831
Zhongyangtuanzao	1.68	357460448	3918032	Gansudongzao	1.85	351593783	3868841
Puchengyuanlizao	1.68	356285800	3935934	Yuanquzao	1.86	362582452	4199932
Anyangtuanzao	1.68	367138227	3963799	Tengzhoudamayazao	1.86	353086888	3803193
Liaochengyuanlingzao	1.68	354289392	4058705	Zunyitianzao	1.86	359283681	3827068
Lianxianmuzao	1.68	357961700	3623993	Guangdongzhenzhuzao	1.86	344193780	3550298
Tai'angedazao	1.69	318616852	3639721	Shaoguanbaizao	1.87	342558340	3602644
Tianjinminzao	1.69	314965735	3475656	Neihuangbianhesuanzao	1.87	360675928	3758567
Lejinzao2	1.69	325816793	3457341	Yanchuanbaizao	1.87	343357694	3949961
Lejinzao1	1.69	327659200	3426444	Dalixiaoyuanzao	1.87	342015186	3776412
Lingbaodazao	1.69	314298029	3576630	Shulutangzao	1.88	352430146	3651887
Nanjingzao (Lanxi)	1.69	339749930	3548522	Tai'andacuizao	1.88	329904809	3515577
Hongzhaoxiaozao	1.69	355376599	3946874	Lianxiankulianzao	1.88	338070955	3859352
Binxianshuizao	1.69	362748808	3843813	Taigulinglingzao	1.89	349893245	3989940
Puchengzhishezao	1.69	359286869	4035676	Yongchengchanghongza o	1.89	358041591	3801648
Tianjinkuaizao	1.69	351872939	3444322	Taiguguoyanhongzao	1.89	342879000	3644363
Hetaowenzaoss1	1.7	325265203	3579998	Binxiansuangedazao	1.89	346043645	3937768
Pingyaobuluosuzao	1.7	364336529	3992766	Hebeilongzao	1.89	340822058	3816173
Zaozhuanggongzao	1.7	353764702	3776314	Yixianmuzao	1.89	345231088	3811272
Lelingmopanzao	1.7	353354208	3539531	Anningxiaozao	1.9	333979384	3973227
Tengzhouluodihongzao	1.7	351702709	3590919	Fucuimizao	1.91	304984483	3877121
Shandongyuanling 78005zao	1.71	319242806	3580726	Xinzhenghuizao	1.91	352641996	3944670
Puchengdundunzao	1.71	337039701	3902568	Zhenpingjiuyuehanzao	1.91	347088367	3787940
Daligedazao	1.71	330170305	3863663	Taiyuanshiyuehongzao	1.92	299641627	3793872
Xianxianmianzao	1.71	325804797	3686066	Yuanlingzao1	1.92	299241303	3511122
Jingudazao	1.71	338819849	3819610	Beijingmayazao	1.92	338802656	3932685
Zaoqiangpozao	1.71	353286122	3826567	Yiwuezizao	1.92	336732410	3579349
Taigushenglizao	1.71	368256967	4031569	Xupujidanzao	1.93	311314568	4065817
Xianxianmuzao	1.71	359023050	3443628	Dingxiangshanzao	1.93	301270952	3809726
Qiyangkangtouzao	1.71	354519983	3546756	Guoxingpingguozhuangz	1.93	314142237	3800680

				ao			
Nanjingdamuzao	1.71	359397323	3914992	Yucimianzao	1.94	315370359	4008840
Puyangsanbianchouzao	1.72	323745864	3784007	Zhongningdiaolingzao	1.94	344231857	3940611
Pingyaokuduanzao	1.72	326468576	3826806	Xinjiangchangyuanzao	1.94	335262726	3721692
Sumuzao	1.72	326184677	3736850	Lelingxiaozao	1.95	333763790	3356107
Wenshuishazao	1.72	325601115	3661365	Yanchuangoutouzao	1.95	341905297	3958806
Xianxianlajiaozao	1.72	369409786	3949804	Dalipachizao	1.96	347684926	3896237
Yanchuantiaozao	1.72	356347297	3734356	Tianjingagazao	1.96	356260689	3724528
Xupuhuluzao	1.72	347272911	3748624	Kuerlexiaozao	1.96	349534460	4202872
Yiwudazao	1.72	334122501	3569812	Linzedazao	1.97	303810305	3985331
Hengyangzhenzhuzao	1.73	314194442	3818440	Lianxiantangzao	1.97	340389783	3959667
Yanzhousanbianshezao	1.73	352927782	3699110	Qufuhoutouzao	1.98	311446583	3946001
Hongzhaohuluzao	1.73	358101569	3726854	Songxiandazao	1.99	336352112	3663177
Dalimayazao	1.73	361726408	3950469	Fengjiejidanzao	1.99	337008735	3957848
Dalijidanzao	1.73	355306714	3995566	Guanyangchangzao	1.99	347982878	3898859
Linzexiaozao	1.73	355655942	3689639	E4nan8zao	2	308718793	4063371
Jiaxianmidiancuimuzao	1.74	325683408	3710707	Beijinglangjiayuanzao	2	354429441	3919622
Dalifengmiguangzao	1.74	328244698	3855060	Dunhuangdazao	2.01	344576849	3934359

Supplementary table 3 Estimated genome heterozygosity and size of 214 wild jujube genotypes based on 30×genome resequencing

Genotype name	Genome heterozygosity（%）	Genome size（bp）	Number of SNPs	Genotype name	Genome heterozygosity（%）	Genome size（bp）	Number of SNPs
LW7-20	1.22	371764192	3800379	Suanzao69	1.75	352751436	4421348
DY44	1.27	353750900	3280031	DY38	1.75	338228397	4349940
Xibeiduanzhi199	1.4	371904397	3708435	Madu8haowuci-2	1.75	337373283	4072092
Shandongsuanzao2	1.45	352703983	3602239	Xingtai0648	1.76	341107172	4117557
LW7-19	1.45	390806108	4176253	Suanzao44	1.76	327544452	3992293
DY37	1.47	372888383	4228308	Lanmao1	1.76	346738468	4259547
Xibeiduanzhi91	1.47	357616284	4108780	Jin1	1.76	330867472	4238827
Xibeiduanzhi171	1.48	359396788	4026864	Xingtai0613	1.76	334913244	4092319
Pingyinzimengsuanzao	1.49	344365072	3471995	Xingtai0609	1.76	332545011	4149844
LW7	1.51	361386291	4349499	C6	1.76	336797666	4162461
Beiketi2-2	1.51	353574176	3937179	Yonghesuanzao4	1.76	333488972	4207571
Xingtai0614	1.52	335730837	3647110	DY6	1.76	328739309	4132964
Suanzao36	1.52	362416773	4268861	Jingxin2	1.76	331850332	4204496
Dongbeisuanzao7	1.53	342242690	3714800	Sandaisizao	1.76	337556144	4393049
DY45	1.53	350585974	3774886	DY32	1.76	334001672	3899134
DY56	1.54	361616845	3944382	Madu8haowuci-1	1.76	332555903	4073614
Xibeiduanzhi152	1.55	353306848	4296999	LW11	1.77	331048296	3938081
Xibeiduanzhi90	1.56	357439412	4349907	Suanzao24	1.77	351171372	4381946
DY43	1.58	360349899	4107457	LW17	1.77	329832518	4019607
N2-18	1.58	355234962	4221106	Yonghesuanzao1	1.77	340929708	4066959
Beiqi11haozao	1.59	347976763	3783455	Suanzao23	1.77	335760204	4171807

Xibeiduanzhi193	1.59	360765104	4238949	Suanzao46	1.77	331186971	4306060
Xibeiduanzhi194	1.59	360980834	4259667	Suanzao32	1.77	340569412	4235066
N1-54	1.6	349360479	4207841	Taizijing	1.77	337390659	4272767
Lanmao2	1.61	356387397	3904616	Tianshi	1.77	342191506	4372510
DY39	1.61	352760306	3954832	BT34	1.78	322034550	3968253
DY20	1.61	337249423	3919707	Suanzao43	1.78	340290546	4160526
DY18	1.61	328012930	3827125	Yonghesuanzao3	1.78	341752941	4341853
LW14-2	1.61	356097020	4297060	C3	1.78	338186353	4124667
Xibeiduanzhi87	1.62	364429041	4484946	DY5	1.78	328514481	4187304
LW15	1.63	335086430	3866214	Dahuzi	1.78	322311491	4283572
Beiketi2	1.63	337342404	3801159	Wanglaofengchenzao	1.78	331824652	4001793
DY30	1.63	338633351	3852376	Xingzhou16	1.78	336690298	4015899
LW1	1.63	353693899	4021510	Shanxichangyuanzao	1.78	330002120	4169597
D6	1.64	357666182	4202570	Zi4	1.79	341534588	4168842
Wenwansuanzao	1.64	346760734	4238060	LW8	1.79	334099694	4194182
Madu8haowuci-5	1.64	340610819	4053327	Xingtai0641	1.79	343512698	4121512
DY24	1.65	327467762	3700317	Zi1	1.79	333684926	4258759
DY9	1.66	334715245	3974678	Zi3	1.79	325823633	4227354
Suanzao40	1.67	343372613	4044346	Suanzao20	1.79	338786652	4187909
DY48	1.67	331227684	4163105	Suanzao8	1.79	334971406	4156186
DY17	1.67	322138580	3807148	Shandongsuanzao5	1.79	326198376	4023542
DY16	1.67	330803836	4630397	DY40	1.79	348724346	4015277
DY2	1.67	328356194	3781618	DY50	1.79	321874894	4101343
No.194	1.67	342312633	3998533	DY35	1.79	340057906	4260162
N2-55	1.67	331527695	4072193	LW13	1.8	340864866	4110184
Dongbeisuanzao3	1.68	336797572	3964575	Xingtai0648	1.8	344536596	4290344
DY46	1.68	346265944	3929205	Yonghesuanzao5	1.8	337519396	4124797
DY21	1.68	341867528	4072461	DY28	1.8	342057736	4138547
Suanzao32	1.68	337415062	4060225	DY8	1.8	330379740	4075861
N2-17	1.68	344211800	4174371	Shanxibianzao	1.8	334160479	4180421
N1-25	1.68	341976558	4231182	N1-67	1.8	323405429	4194886
N1-10	1.68	329583447	4056594	N1-66	1.8	342561453	4263890
N1-7	1.68	339359928	4277722	Suanzao30	1.81	343823064	4276955
LW7-18	1.68	362577509	4166421	D12	1.81	326844281	4216139
Suanzao71	1.69	353128394	4357439	Suanzao11	1.81	329403285	4247247
DY13	1.69	341986060	4068050	Dongbeisuanzao5	1.81	335024352	4191420
DY11	1.69	333274306	4116108	Xingtai16	1.81	327635785	4231203
N1-72	1.69	330089177	4094635	Suanzao1	1.81	328779816	4365980
C16	1.7	333262581	4199330	Suanzao41	1.81	344794487	4130668
GaoweishengsuCsuanzao	1.7	345702243	4040961	DY29	1.81	315032638	4108919
LW14	1.71	335434542	4128082	DY55	1.81	328288950	4253863
ShandonggaoweishengsuC	1.71	336830735	4059320	Gaotaisuanzao	1.81	335360217	4304306
DY22	1.71	342902968	4115411	Suanzao47	1.82	341420895	4247031
DY55	1.71	346379282	4224895	LW7	1.82	332117389	4227600

DY57	1.71	338503064	4284381	LW16	1.82	323300522	4149389
DY33	1.71	336383576	4178708	Tesourzao4	1.82	336206663	4261978
N2-1	1.71	349393776	4129340	Shanxixiaosuanzao	1.82	328294914	4332056
N1-34	1.71	352195416	4285671	Liaoning Chaoyang2	1.82	339385502	4300251
Suanzao14	1.72	348884911	4053024	DY41	1.82	329526654	4184823
Suanliuliuzao	1.72	341086675	4194988	DY31	1.82	332894312	4222434
Suanzao42	1.72	338382894	4214061	Xingtai10	1.82	329727181	4213798
Suanzao15	1.72	346833791	4221583	Suanzao73	1.82	334591511	4230134
Xingtai10	1.72	338740281	3924731	Taihang1	1.82	339488703	4355476
DY4	1.72	328072976	3996356	Xibeiduanzhi109	1.82	350863333	4049212
D1Y2	1.72	353469079	4230254	Xingtai0608	1.83	329253463	4093156
DY53	1.72	350142002	3933069	Suanzaowang 3	1.83	335179059	4283464
Suanzao45	1.73	330582047	3974348	Suanzao48	1.83	338076175	4298045
LW66	1.73	333624552	3958015	Dongbeisuanzao4	1.83	333324070	4268920
Chengde5	1.73	347170945	4196880	DY42	1.83	337543438	4318848
Suanzao3	1.73	348579397	4217518	DY15	1.83	331637151	4102048
Henansuanzao1	1.73	330348983	3959449	Huzhuanglaozaoshu	1.83	332438465	4073348
Suanzao15	1.73	331014309	4086987	Bianhesuanzao	1.84	334034171	4235337
Beiketi1	1.73	341666917	4034374	T37	1.84	325588994	4327212
DY23	1.73	333793199	4028057	Daguofengchan3	1.84	327355694	4242985
DY3	1.73	334843414	4136159	Zhongdaguofengshaocikougan hao5	1.84	320511794	4153575
Zaoyan3	1.73	352941922	4274345	Xingzhou9	1.84	327676065	4105285
LW2	1.73	340682504	4371114	Chengwanwucizao3	1.85	335960313	3987223
Dongbeisuanzao6	1.74	338865429	4024364	DY34	1.85	325477910	4088892
C22	1.74	330754422	4084598	LW3	1.85	344480358	4283995
Shandongsuanzao3	1.74	331908517	4087071	LW14zidai55	1.85	343671770	3737413
Suanzao17	1.74	347102039	4234328	Xingtai0619	1.86	331807039	4206786
Dongbeisuanzao	1.74	334386824	3892640	Zhongdaguotefeng1	1.86	322072536	4135323
Yonghesuanzao1	1.74	333212348	4078307	Madu18	1.86	338013268	4156705
DY49	1.74	334110346	4140676	DY26	1.87	319349912	4369053
DY14	1.74	349905221	4083407	DY54	1.88	319649330	4418412
DY1	1.74	342742141	4215391	Xianshi	1.89	342122328	3962802
Xingzhou2	1.74	336698791	3992338	DY12	1.89	332157654	4218315
Sandaiyizao	1.74	336813106	4280715	LW12	1.9	322458517	4063162
F15	1.74	329656421	4262997	Cangzhou 1	1.91	319627525	4358913
Madu8haowuci-3	1.74	338590488	4097671	DY7	1.91	345650651	3946430
Madu8haowuci-4	1.74	334007144	4121139	LW4	1.91	311488539	4174014
Xingtai0610	1.75	342130856	4224829	LW5	1.97	315087204	4318284
DY19	1.75	329341404	4064896	DY25	2	334971698	4076454
Fupingcentenariansuanzao	1.75	323414279	4247275	Xibeiduanzhi67	2.07	335434578	5031518
Shanxiyuanzao	1.75	328716999	4121609	DY36	2.09	339976356	4224053
Zhongchanfengguoshaoci2	1.75	327345157	4040788	D1Y3	2.13	321106210	4190805

Supplementary table 4 Summary of Sequencing Output and Quality Control Metrics

Metric	Mean ± SD	Minimum	Maximum
Raw Reads (Million)	95.23 ± 8.18	32.71	163.39
Clean Reads (Million)	95.23 ± 8.18	32.71	163.39
Data Retention Rate(%)	99.99 ± 0.00	99.97	99.99
Q20 Rate (%)	98.52 ± 1.03	96.00	99.60
Q30 Rate (%)	95.12 ± 2.89	87.71	98.28
GC Content (%)	34.53 ± 0.85	32.07	37.94
Raw Data Volume (Gb)	27.15 ± 5.53	9.61	48.03
Clean Data Volume (Gb)	27.15 ± 5.53	9.61	48.03

Supplementary table 5 Sequencing Data Quality Assessment Summary for Variety 510

Variety name	Raw_Reads(M)	Clean_Reads(M)	Retention(%)	Q20(%)	Q30(%)	GC(%)
Suanliuliuzao	95.01	95.01	99.99	99.37	97.67	34.03
Xianshi	97.82	97.82	99.99	99.5	97.86	34.61
Chengwanwucizao3	95.79	95.79	99.99	99.18	97.03	34.26
Bianhesuanzao	96.85	96.85	99.99	99.53	97.96	34.7
Xingtai0619	93.78	93.78	99.99	99.26	97.26	34.63
Xingtai0648	97.9	97.9	99.99	99.52	97.95	34.44
Suanzao47	95.02	95.02	99.99	99.54	98.01	34.1
Zi4	93.76	93.76	99.99	99.3	97.41	34.47
Xingtai0610	96.94	96.94	99.99	99.53	98	34.03
Suanzao30	97.18	97.18	99.99	99.27	97.28	33.94
D12	99.87	99.87	99.99	99.5	97.84	34.56
BT34	97.91	97.91	99.99	99.05	96.65	34.54
Suanzao45	102.89	102.89	99.99	99.24	97.19	33.84
Suanzao43	95.23	95.23	99.99	99.21	97.05	33.87
Beiqi11haozao	97.74	97.74	99.99	99.23	97.21	33.01
Dongbeisuanzao6	94.33	94.33	99.99	99.2	97	34.21
Suanzao44	99.3	99.3	99.99	99.16	97	34.45
LW7	97.2	97.2	99.99	99.25	97.16	33.75
LW8	97.49	97.49	99.99	99.23	97.24	34.05
LW11	96.51	96.51	99.99	99.19	97.01	34.15
LW12	96.39	96.39	99.99	98.93	96.08	34.67
LW13	94.1	94.1	99.99	99.23	97.06	33.85
LW14	94.47	94.47	99.99	99.23	97.16	33.71
Suanzao24	93.81	93.81	99.99	99.18	96.98	33.47
LW15	92.65	92.65	99.99	99.23	97.2	33.91
LW16	97.83	97.83	99.99	99.13	96.83	34.35
LW17	95.05	95.04	99.99	99.22	97.13	34.24
LW66	98.72	98.72	99.99	99.33	97.56	33.34
Suanzao11	101.69	101.69	99.99	99.23	97.21	33.95
Xingtai0648	98.93	98.92	99.99	99.53	98	33.2
Lanmao1	98.94	98.94	99.99	99.55	98.08	34
Lanmao2	96.93	96.93	99.99	99.12	96.83	33.61

Tesourzao4	99.35	99.35	99.99	99.46	97.67	34.58
Yonghesuanzao1	98.33	98.33	99.99	99.24	97.2	33.43
Jin1	101.24	101.24	99.99	99.48	97.77	34.1
Xingtai0641	96.61	96.61	99.99	99.31	97.42	33.43
Xingtai0608	101.15	101.15	99.99	99.46	97.64	34.6
Zi1	107.37	107.36	99.99	99.49	97.8	34.4
Chengde5	97.92	97.92	99.99	99.5	97.89	33.78
Xingtai0613	98.19	98.19	99.99	99.28	97.31	34.71
Suanzaowang 3	90.57	90.57	99.99	99.5	97.85	34.33
C22	97.69	97.69	99.99	99.5	97.85	34.15
Zi3	100.73	100.73	99.99	99.22	97.12	34.05
T37	94.88	94.88	99.99	99.49	97.78	34.25
Dongbeisuanzao3	94.61	94.61	99.99	99.44	97.61	34.14
C16	101.72	101.72	99.99	99.55	98.05	33.79
Yonghesuanzao3	99.08	99.08	99.99	99.16	96.96	33.7
Suanzao48	98.63	98.63	99.99	99.5	97.81	34.22
Xingtai0609	99.15	99.15	99.99	99.19	96.97	33.82
Dongbeisuanzao5	96.45	96.45	99.99	99.48	97.78	34.42
Xingtai16	102.52	102.52	99.99	99.34	97.5	34.46
Suanzao20	97.06	97.06	99.99	99.48	97.75	33.93
Suanzao23	95.54	95.54	99.99	99.49	97.81	34.23
Suanzao3	96.7	96.7	99.99	99.5	97.88	34.03
ShandonggaoweishengsuC	97.97	97.97	99.99	99.25	97.29	33.97
Shanxixiaosuanzao	99.43	99.43	99.99	99.51	97.88	34.2
C3	96.25	96.25	99.99	99.16	96.85	33.67
Suanzao1	99.64	99.64	99.99	99.52	97.94	34.17
Dongbeisuanzao4	97.3	97.3	99.99	99.28	97.28	33.89
Shandongsuanzao3	101.1	101.1	99.99	99.47	97.74	33.88
C6	96.93	96.93	99.99	99.5	97.83	33.57
Suanzao71	98.66	98.66	99.99	99.57	98.16	33.65
Suanzao17	98.96	98.96	99.99	99.19	97.04	34.49
Suanzao14	94.56	94.56	99.99	99.51	97.87	34.28
Yonghesuanzao5	96.97	96.96	99.99	99.25	97.22	34.06
Suanzao8	99.74	99.74	99.99	99.47	97.7	34.38
Dongbeisuanzao	91.44	91.44	99.99	99.26	97.19	33.97
Suanzao46	114.15	114.15	99.99	99.47	97.75	33.83
Suanzao40	96.13	96.13	99.99	99.46	97.69	33.54
Shandongsuanzao2	97.53	97.53	99.99	99.51	97.91	33.24
Xingtai0614	98.94	98.94	99.99	99.25	97.17	33.77
Suanzao42	96.26	96.26	99.99	99.49	97.81	33.57
Henansuanzao1	98.44	98.44	99.99	99.1	96.75	33.9
Suanzao15	98.96	98.96	99.99	99.51	97.9	34.09
Shandongsuanzao5	95.45	95.45	99.99	99.19	97.01	34.2
Liaoning Chaoyang2	91.31	91.31	99.99	99.43	97.56	33.9

Suanzao32	94.92	94.92	99.99	99.49	97.78	33.84
Suanzao4	94.7	94.7	99.99	99.5	97.86	33.44
Dongbeisuanzao7	99.81	99.81	99.99	99.24	97.19	33.76
Yonghesuanzao4	100.47	100.47	99.99	99.5	97.85	34.38
Beiketi2	95.52	95.52	99.99	99.07	96.45	33.45
Yonghesuanzao1	98.28	98.28	99.99	99.44	97.58	34.03
Suanzao41	92.32	92.32	99.99	99.19	97.05	35.18
Beiketi1	98.73	98.73	99.99	99.16	96.94	34.32
Xingtai10	99.13	99.13	99.99	99.09	96.65	34.28
D6	99.83	99.83	99.99	99.54	98.03	33.55
GaoweishengsuCsuanzao	90.55	90.55	99.99	99.27	97.25	33.93
Wenwansuanzao	99.4	99.4	99.99	99.51	97.89	33.57
DY39	95.3	95.3	99.99	99.24	97.11	34.32
DY40	97.78	97.78	99.99	99.51	97.9	34.63
DY41	95.72	95.72	99.99	99.24	97.12	34.93
DY42	102.09	102.09	99.99	99.49	97.8	34.25
DY43	98.73	98.73	99.99	99.52	97.94	33.09
DY44	98.92	98.92	99.99	99.57	98.19	33.58
DY45	100.01	100.01	99.99	99.2	97.12	33.53
DY46	94.78	94.78	99.99	99.18	97.06	33.65
DY48	117.74	117.74	99.99	99.37	97.51	34.1
DY49	99.46	99.46	99.99	99.22	97.09	34.07
DY50	95.9	95.9	99.99	99.12	96.84	34.26
DY54	119.39	119.39	99.99	99.4	97.66	34.74
DY31	111.63	111.63	99.99	99.31	97.33	34.14
DY30	99.98	99.98	99.99	99.49	97.83	33.53
DY29	100.11	100.11	99.99	99.18	97.05	34.4
DY28	96.99	96.99	99.99	99.19	97.05	33.9
DY26	112.39	112.39	99.99	99.33	97.43	34.15
DY25	99.83	99.83	99.99	99.29	97.39	34.6
DY24	98.78	98.78	99.99	99.54	98.03	34.07
DY23	97.49	97.49	99.99	99.51	97.88	34.06
DY22	103.15	103.15	99.99	99.36	97.54	33.56
DY21	95.13	95.13	99.99	99.27	97.29	33.52
DY20	96.89	96.89	99.99	99.56	98.09	33.91
DY19	100.11	100.11	99.99	99.23	97.14	33.96
DY18	101.03	101.03	99.99	99.2	97.07	33.94
DY17	98.69	98.69	99.99	99.16	96.99	34.18
DY16	163.39	163.39	99.99	99.46	97.86	34.49
DY15	91.06	91.06	99.99	99.51	97.89	34.25
DY14	97.34	97.34	99.99	99.54	98.03	34.1
DY13	92.15	92.15	99.99	99.08	96.8	33.43
DY12	100.22	100.22	99.99	99.49	97.81	34.63
DY11	98.54	98.54	99.99	99.33	97.58	33.97

DY9	98.37	98.37	99.99	99.26	97.26	34.14
DY8	98.57	98.57	99.99	99.53	97.96	34.24
DY55	97.19	97.19	99.99	99.53	97.99	33.78
DY1	99.2	99.2	99.99	99.56	98.14	33.49
DY2	98.38	98.38	99.99	99.08	96.73	34.27
DY3	99.17	99.17	99.99	99.32	97.43	33.65
DY4	99.1	99.1	99.99	99.2	97.12	34.35
DY5	99.9	99.9	99.99	99.2	97.09	34.14
DY6	99.33	99.33	99.99	99.31	97.39	34.06
Jingxin2	100.19	100.19	99.99	99.53	97.98	34.21
Taizijing	96.13	96.13	99.99	99.54	98.04	34.02
Zaoyan3	101.57	101.57	99.99	99.32	97.47	33.7
Dahuzi	110.55	110.55	99.99	99.57	98.16	34.02
Suanzao32	96.31	96.31	99.99	99.3	97.43	33.76
Shanxibianzao	97.65	97.65	99.99	99.31	97.5	33.57
Fupingcentenariansuanzao	100.52	100.52	99.99	99.51	97.9	33.55
Shanxiyuanzao	99.52	99.52	99.99	99.3	97.42	33.82
Cangzhou 1	103.37	103.37	99.99	99.54	98.04	34.43
DY55	97.98	97.98	99.99	99.21	97.04	33.46
DY56	96.99	96.99	99.99	99.21	97.06	32.87
DY57	114.43	114.43	99.99	99.48	97.76	34
Xingtai10	97.33	97.33	99.99	99.18	97.09	34.31
Zhongdaguotefeng1	97.28	97.28	99.99	98.94	96.02	34.18
Zhongchanfengguoshaoci2	100.03	100.03	99.99	99.22	97.16	34.32
Daguofengchan3	101.87	101.87	99.99	99.2	97.06	34.66
Zhongdaguofengshaocikouganhao5	98.83	98.83	99.99	99.14	96.85	34.14
Suanzao73	100.91	100.91	99.99	99.28	97.31	34
D1Y2	100.25	100.25	99.99	99.51	97.94	33.5
Suanzao69	111.23	111.23	99.99	99.46	97.7	33.4
No.194	95.63	95.63	99.99	99.47	97.75	33.7
Huzhuanglaozaoshu	97.62	97.62	99.99	99.24	97.17	34.44
Xingzhou2	94.36	94.36	99.99	99.3	97.42	33.96
Xingzhou9	95.5	95.5	99.99	99.31	97.39	34.36
Wanglaofengchenzao	98.1	98.1	99.99	99.28	97.41	33.64
Taihang1	101.03	101.03	99.99	99.21	97.03	33.7
Tianshi	99.39	99.39	99.99	99.22	97.18	33.21
Sandaiyizao	105.64	105.64	99.99	99.45	97.66	33.92
Sandaisizao	103.67	103.67	99.99	99.45	97.76	34.01
Pingyinzimengsuanzao	93.63	93.63	99.99	99.33	97.44	34.02
Xingzhou16	93.35	93.35	99.99	99.24	97.17	33.8
D1Y3	93.05	93.05	99.99	99.16	96.92	34.83
Shanxichangyuanzao	97.43	97.43	99.99	99.24	97.18	33.83
Gaotaisuanzao	96.59	96.59	99.99	99.29	97.36	33.68
Suanzao36	99.97	99.97	99.99	99.41	97.69	32.94

F15	123.77	123.77	99.99	99.42	97.54	33.82
DY53	94.21	94.21	99.99	99.5	97.88	33.97
DY32	93.63	93.63	99.99	99.24	97.14	34.06
DY33	94.32	94.32	99.99	99.52	97.92	33.63
DY34	93.97	93.97	99.99	99.19	96.99	34.36
DY35	95.4	95.4	99.99	99.24	97.2	33.55
DY36	99.63	99.62	99.99	99.44	97.61	34.29
DY37	94.58	94.58	99.99	99.46	98	32.85
DY38	114.59	114.59	99.99	99.45	97.67	33.91
DY7	94.03	94.03	99.99	99.23	97.12	34.21
N2-1	97.02	97.02	99.99	99.22	97.06	33.68
N1-72	99.04	99.04	99.99	99.19	97.03	33.88
N1-67	92.99	92.99	99.99	99.15	96.77	34.1
N1-66	101.81	101.81	99.99	99.27	97.15	34.28
N2-17	98.7	98.7	99.99	99.27	97.31	33.78
N2-18	99.3	99.3	99.99	99.29	97.4	33.49
N1-54	100.66	100.66	99.99	99.22	97.07	33.42
N1-34	97.81	97.81	99.99	99.5	97.84	33.68
N1-25	96.97	96.97	99.99	99.25	97.22	33.33
N2-55	102.2	102.2	99.99	99.18	96.92	33.91
N1-10	100.79	100.79	99.99	99.31	97.39	34.25
N1-7	100.04	100.04	99.99	99.59	98.28	33.14
LW1	100.57	100.57	99.99	99.53	97.95	33.74
LW2	100.23	100.23	99.99	99.54	98.06	33.66
LW3	98.04	98.04	99.99	99.08	96.71	34.31
LW4	101.55	101.55	99.99	99.49	97.78	34.58
LW5	98.97	98.97	99.99	99.08	96.62	34.31
Madu8haowuci-1	93.8	93.8	99.99	99.49	97.79	34.15
Madu8haowuci-2	95.58	95.58	99.99	99.12	96.82	33.93
Madu8haowuci-3	97.46	97.46	99.99	99.53	97.97	34.48
Madu8haowuci-4	96.97	96.97	99.99	99.26	97.31	33.78
Madu8haowuci-5	98.15	98.15	99.99	99.59	98.26	33.88
LW7-18	89.13	89.12	99.98	97.85	93.79	34.28
LW7-19	80.94	80.93	99.98	98.4	95.37	33.01
LW7-20	94.58	94.57	99.98	98.19	94.77	33.81
LW14zidai55	80.86	80.84	99.98	97.79	93.61	34.57
Xibeiduanzhi67	99.54	99.54	99.99	99.53	97.96	35.03
Xibeiduanzhi87	95.32	95.31	99.98	98.45	95.62	33.46
Xibeiduanzhi90	88.9	88.88	99.98	98.45	95.5	33.93
Xibeiduanzhi91	81.53	81.52	99.98	98.18	94.73	34.45
Xibeiduanzhi109	79.09	79.08	99.99	97.99	94.28	34
Xibeiduanzhi152	90.79	90.77	99.98	98.34	95.22	34.08
Xibeiduanzhi171	80.71	80.69	99.98	98.2	94.82	34.45
LW7	92.3	92.28	99.98	98.42	95.44	33.72

Xibeiduanzhi190	93.43	93.41	99.98	98.5	95.76	33.69
Xibeiduanzhi193	94.29	94.28	99.98	98.43	95.59	33.94
Xibeiduanzhi194	91.63	91.62	99.98	98.7	96.34	33.96
Beiketi2-2	89.78	89.76	99.98	98.54	95.92	34.12
Madu18	88.66	88.65	99.98	98.33	95.25	34.56
Xibeiduanzhi199	80.2	80.2	99.99	98.13	94.72	34.13
Jishanbanzao	85.88	85.87	99.98	98.36	94.61	37.94
Linyilizao	87.06	87.05	99.98	97.95	93.31	35.71
Yucituanzao	87.39	87.37	99.98	97.87	93.01	35.41
Taiguduanzizao	87.73	87.72	99.98	97.75	92.63	35.4
Taiguhuluzao	86.68	86.66	99.98	98.1	93.75	35.32
Pingyaobuluosuzao	87.32	87.3	99.98	97.89	93.05	35.37
Pinglujianzao	86.97	86.95	99.98	97.99	93.43	35.44
Linfenmizao	88.1	88.08	99.98	97.64	92.26	35.77
Linfenzhenhuluzao	86.64	86.63	99.98	98.13	93.8	34.73
Yongjihamazao	88.07	88.05	99.98	97.59	92.24	34.82
Jiaochengjunzao	87.35	87.33	99.98	97.9	93.03	36.18
Taiguheiyezao	87.69	87.68	99.98	97.73	92.69	36.23
Yunchengpopozao	89.04	89.03	99.98	97.3	91.24	35.2
Pinglutuntunzao	86.99	86.98	99.98	98.02	93.44	35.35
Beijingpaopaozao	87.33	87.31	99.98	97.89	93.04	34.95
Baodeyouzao	87.62	87.61	99.98	97.82	92.78	35.05
Taigulangzao	86.9	86.88	99.98	98.01	93.55	35.28
Xiangzao	86.95	86.93	99.98	98.01	93.44	35.16
Hongzhaocuizao	86.79	86.77	99.98	98.09	93.69	34.58
Lichengxiaozao	87.15	87.14	99.98	97.98	93.24	34.84
Taigudundunzao	87.93	87.91	99.98	97.69	92.4	35.01
Xiangfenguantanzao	87.34	87.33	99.98	97.87	93.04	35.06
Taigumeimizao	88.56	88.55	99.98	97.33	91.75	34.85
Beijingbenzao	86.93	86.92	99.98	97.95	93.46	35.34
Xiangfenyuanzao	87.08	87.07	99.98	97.93	93.46	34.82
Hongzhaohuluzao	88.13	88.12	99.98	97.49	92.22	35.91
Baodexiaozao	87.92	87.91	99.98	97.55	92.46	35.28
Jishanyuanzao	88.49	88.47	99.98	97.35	91.8	35.89
Hongzhaoshiyuehongzao	87.29	87.27	99.98	97.91	93.11	34.99
Zhongyangmuzao	88	87.98	99.98	97.65	92.32	34.43
Pingshunjunzao	88.38	88.36	99.98	97.52	91.95	35.06
Dingxiangxingxingzao	88.12	88.1	99.98	97.61	92.24	35.13
Taigulinglingzao	86.17	86.15	99.98	98.32	94.3	36.19
Xiangfenmuzao	86.27	86.26	99.98	98.27	94.24	34.29
Jishanliuguanzao	86.76	86.74	99.98	97.97	93.69	35.18
Zhongyangtuanzao	88.03	88.02	99.98	97.52	92.35	35.16
Xiangfenyazao	87.6	87.58	99.98	97.67	92.77	35.11
Yuciyazao	87.78	87.76	99.98	97.61	92.57	35.17

Jiaochengtiansuanzao	90.11	90.1	99.98	96.77	90.19	35.16
Yuanquzao	88.83	88.82	99.98	97.26	91.5	35.53
Hongzhaoxiaozao	87	86.98	99.98	97.88	93.46	35.68
Xiaxianyuancuizao	88.55	88.53	99.98	97.32	91.76	35.36
Pingshunbenzao	88.44	88.43	99.98	97.38	91.9	35.32
Jiaochengduanzao	88.12	88.11	99.98	97.48	92.26	35.45
Xiaxianziyuanzao	87.94	87.92	99.98	97.52	92.43	35.6
Linfentuanzao	90.16	90.14	99.98	96.73	90.16	35.59
Shanxiqiyuexianzao	88.55	88.54	99.98	97.33	91.78	35.29
Zaoqiangpozao	88.77	88.76	99.98	97.23	91.54	35.48
Qingyuandadanzao	86.77	86.75	99.98	97.97	93.67	35.71
Taigushenglizao	88.82	88.8	99.98	97.27	91.51	34.97
Xinzhengdamayazao	88.99	88.97	99.98	97.19	91.32	35.57
Dalixiaodundunzao	87.68	87.66	99.98	97.61	92.7	35.17
Binxianshuizao	87.93	87.91	99.98	97.54	92.42	35.73
Daliyuanzao	87.91	87.9	99.98	97.51	92.43	35.16
Yanchuanniunaicuizao	88.13	88.12	99.98	97.49	92.21	34.56
Zaoqianggutouxiaozao	88.76	88.74	99.98	97.27	91.57	35.53
Gusuxiaozao	88.6	88.59	99.98	97.31	91.72	35.65
Xinzhengjidanzao	87.15	87.13	99.98	97.82	93.27	35.61
Huanghuadongzao	87.51	87.49	99.98	97.71	92.86	34.8
Dalimayazao	89.14	89.13	99.98	97.1	91.2	35.59
Puchengzhishezao	88.16	88.14	99.98	97.42	92.18	35.29
Yanchuandabaizao	88.75	88.74	99.98	97.25	91.6	36.01
Xianxianlajiaozao	87.61	87.59	99.98	97.65	92.77	35.57
Yongchengyuanhongzao	88.78	88.76	99.98	97.27	91.49	34.96
Lintongguluzao	87.88	87.86	99.98	97.58	92.48	35.43
Cangxiantunzizao	86.88	86.86	99.98	97.93	93.57	35.22
Xianxianxiaoxiaozao	87.91	87.89	99.98	97.57	92.45	35.07
Neihuangbianhesuanzao	88.9	88.88	99.98	97.24	91.43	35.29
Xincaidayuanfengzao	88.6	88.59	99.98	97.29	91.74	35.82
Linxianwutouzao	89.27	89.26	99.98	97.09	91.02	34.95
Dalijidanzao	89.2	89.18	99.98	97.07	91.08	35.54
Binxianheigadazao	88.91	88.89	99.98	97.23	91.37	35.35
Daliganweibazao	88.38	88.37	99.98	97.34	91.95	35.45
Xianxianmuzao	88.52	88.5	99.98	97.2	91.81	35.05
Zhenpingtailihongzao	88.87	88.85	99.98	97.06	91.41	35.2
Yongchengchanghongzao	90.65	90.63	99.98	96.42	89.62	35.53
Yanchuanbaizao	88.05	88.03	99.98	97.37	92.32	35.65
Zaoqiangcuizao	90.83	90.82	99.98	96.35	89.41	36.24
Xinzhengqitoubaizao	88.69	88.68	99.98	97.12	91.58	34.61
Zaoqiangmalianxiaozao	90.96	90.95	99.98	96.45	89.34	34.54
Cangxianxiaozao	90.03	90.02	99.98	96.69	90.23	35.89
Taigumeixinhongzao	88.83	88.81	99.98	97.11	91.55	35.32

Taiguguoyanhongzao	89.63	89.62	99.98	96.79	90.69	35.72
Yanchuandieyazao	89.62	89.61	99.98	96.85	90.71	36.77
Shanxinaizao	89.74	89.73	99.98	96.85	90.57	36.1
Tengzhouchanghongzao	90.12	90.1	99.98	96.6	90.16	34.67
Shulutangzao	89.85	89.84	99.98	96.73	90.46	35.57
Cangxianjinsixiaozao	90.09	90.08	99.98	96.62	90.19	35.64
Xinzhenghuizao	89.72	89.7	99.98	96.96	90.6	35.66
Xinzhengxiaoyuanzao	88.2	88.19	99.98	97.48	92.15	35.6
Yanchuantiaozao	89.63	89.61	99.98	97.02	90.73	35.5
Puchengyuanlizao	90.11	90.09	99.98	96.83	90.19	34.44
Binxiansuangedazao	92.64	92.63	99.98	96	87.71	35.57
Shanximianzao	89.72	89.71	99.98	96.98	90.59	35.56
Lelingwuhexiaozao	89.2	89.18	99.98	97.17	91.1	35.17
Lelingxiaozao	89.83	89.82	99.98	96.97	90.47	36.9
Dayewuhezao	90.43	90.41	99.98	96.71	89.86	34.56
Dalipachizao	89.55	89.54	99.98	97.03	90.72	35.9
Zhongcaobenzao	90.25	90.24	99.98	96.79	90	35.25
Yutianxiaozao	89.93	89.92	99.98	96.88	90.37	35.34
Puyangsanbianhongzao	89.22	89.21	99.98	97.12	91.05	34.64
Hebeilongzao	90.65	90.63	99.98	96.68	89.64	35.38
Hanguowudengzao	88.33	88.32	99.98	97.43	91.98	35.57
Anyangtuanzao	89.19	89.17	99.98	97.08	91.18	35.28
Dalilongzao	88.42	88.4	99.98	97.35	91.91	35.8
Binxianjinzao	87.53	87.51	99.98	97.66	92.84	35.41
Liaochengyuanlingzao	89.73	89.71	99.98	96.89	90.58	34.63
Puyangtangzao	88.56	88.54	99.98	97.33	91.75	35.36
Zhenpingguangyangzao	88.04	88.02	99.98	97.5	92.31	35.33
Zaozhuanggongzao	87.34	87.32	99.98	97.74	93.05	35.5
Hamidazao	88.76	88.74	99.98	97.21	91.54	35.27
Zaoqiangshazao	88.55	88.53	99.98	97.32	91.78	35.9
Xuechengdongzao	88.29	88.27	99.98	97.4	92.04	35.5
Xinzhengchangjixinzao	88.37	88.36	99.98	97.37	91.95	35.25
Yangjiaozao	88.77	88.76	99.98	97.25	91.56	35.03
Lelingmopanzao	89.07	89.05	99.98	97.16	91.24	35.48
Ningyangxuanlingzao	90.63	90.62	99.98	96.7	89.66	35.45
Tai'andacuizao	90.31	90.3	99.98	96.86	89.98	35.76
Chengwudongzao	89.45	89.44	99.98	97.09	90.85	35.64
Tengzhoudamayazao	88.51	88.5	99.98	97.36	91.81	35.71
Zhenpingjiuyuehanzao	89.39	89.38	99.98	96.99	90.92	35.84
Lengbaiyuzao	89.16	89.14	99.98	97.18	91.19	35.68
Xi'anyangnaizao	89.29	89.27	99.98	97.1	91.02	35.59
Jiaxianyazao	89.34	89.33	99.98	97.11	90.97	34.85
Yanchuangoutouzao	91.2	91.18	99.98	96.47	89.08	35.71
Shanxihuluzao	90.24	90.22	99.98	96.79	90.07	36.11

Dalixiaoyuanzao	89.91	89.89	99.98	96.92	90.41	35.63
Xinzhengjiuyueqingzao	89.21	89.2	99.98	97.15	91.13	35.05
Puyangxiaozao	89.26	89.25	99.98	97.1	91	35.15
Puyanghetaowenzao	89.05	89.03	99.98	97.2	91.25	35.3
Songxiandazao	89.59	89.57	99.98	96.99	90.69	36.25
Tai'anmalingcuizao	90.18	90.17	99.98	96.79	90.07	35.42
Ningyangdashibingzao	90.12	90.11	99.98	96.84	90.14	35.39
Beibeixiaozao	90.02	90	99.98	96.89	90.3	34.5
Tengzhouluodihongzao	89.93	89.91	99.98	96.95	90.34	36.27
Fengjiejidanzao	90.2	90.19	99.98	96.79	90.08	36.04
Qingyunxiaolizao	89.32	89.31	99.98	97.1	90.98	35.31
Beijingmayazao	90.13	90.12	99.98	96.84	90.16	35.74
Beijingyingluozao	88.81	88.79	99.98	97.28	91.52	35.4
Tianjingagazao	89.12	89.11	99.98	97.15	91.16	36.29
Tianjinkuaizao	90.67	90.65	99.98	96.67	89.63	35.3
Beijinglangjiayuanzao	90.4	90.38	99.98	96.72	89.87	36.03
Qiyangkangtouzao	89.58	89.57	99.98	97.02	90.68	35.16
Xupudaguosuanpanzao	87.87	87.85	99.98	97.46	92.5	36.1
Hengshanchangdazao	87.69	87.67	99.98	97.51	92.66	35.33
Xuputiansuanzao	89.37	89.35	99.98	97.16	90.96	35.76
Xupuhuluzao	88.22	88.21	99.98	97.32	92.12	34.85
Hunanchangzao	87.77	87.76	99.98	97.5	92.58	35.23
Xupubinglangzao	89.16	89.14	99.98	97.06	91.15	35.43
Xupumuzao	88.06	88.04	99.98	97.4	92.3	35.76
Xupuyuanzao	88.83	88.82	99.98	97.26	91.5	35.5
Lanximazao	87.98	87.97	99.98	97.43	92.36	35.89
Shengxianbaipuzao	88.04	88.02	99.98	97.44	92.33	35.36
Xuanchengjianzao	88.51	88.49	99.98	97.24	91.82	35.53
Nanjingdamuzao	88.06	88.04	99.98	97.39	92.3	35.05
Langxiniunaizao	88.37	88.35	99.98	97.28	91.95	35.51
Yiwuezizao	88.3	88.28	99.98	97.31	92.12	35.94
Fuyangmayizao	88.22	88.2	99.98	97.38	92.17	35.24
Yiwudazao	87.75	87.74	99.98	97.5	92.6	35.26
Hubeilingdangzao	88.94	88.92	99.98	97.09	91.36	35.32
Gansudongzao	91.99	91.97	99.98	96.38	88.38	36.04
Hubeiyuanzao	88.56	88.54	99.98	97.25	91.78	35.44
Zhongningdiaolingzao	88.34	88.32	99.98	97.29	91.97	35.56
Zhongningxiaoyuanzao	89.4	89.38	99.98	96.97	90.9	35.57
Guanyangchangzao	88.82	88.81	99.98	97.14	91.46	35.74
Nanjingyazao	88.1	88.09	99.98	97.39	92.24	35.35
Guanyangduanzao	87.64	87.63	99.98	97.54	92.72	35.3
Wuxianshuituanzao	88.13	88.12	99.98	97.38	92.2	35.76
Nanjinglengzao	88.38	88.37	99.98	97.3	91.95	34.71
kashengaerxiaozao	87.79	87.78	99.98	97.5	92.55	35

Linzexiaozao	88.16	88.14	99.98	97.37	92.16	35.41
Dunhuangdazao	88.66	88.65	99.98	97.18	91.67	35.94
Chaoyangdajiandingzao	87.81	87.8	99.98	97.5	92.5	35.98
Kuerlexiaozao	87.84	87.82	99.98	97.46	92.49	35.74
Xinjiangchangyuanzao	87.79	87.77	99.98	97.49	92.57	36.5
Chaoyangdapingdingzao	87.91	87.9	99.98	97.47	92.46	34.38
Anningxiaozao	89.04	89.02	99.98	97.09	91.27	35.47
Minqinxiaozao	88.46	88.45	99.98	97.24	91.87	35.44
Aksuxiaozao	89.24	89.23	99.98	96.99	91.04	35.17
Zunyitianzao	89.06	89.04	99.98	97.05	91.19	35.36
Lianxiantangzao	89.42	89.4	99.98	96.96	90.86	35.6
Lianxianmuzao	88.14	88.13	99.98	97.36	92.17	35.19
Yixianmuzao	88.11	88.1	99.98	97.39	92.27	35.79
Shaoguanbaizao	87.77	87.75	99.98	97.52	92.59	34.8
Xishuangbannaxiaozao	88.49	88.47	99.98	97.24	91.82	35.42
Gendedazao	88.47	88.46	99.98	97.23	91.84	35.96
Lianxiankulianzao	89.39	89.38	99.98	97.04	90.92	35.54
Guangdongzhenzhuzao	89.44	89.42	99.98	96.94	90.84	36.01
Kunmingzao	89.42	89.41	99.98	97.05	90.9	35.22
Huluchanghongzao	87	86.98	99.98	97.76	93.35	34.55
Neihuangpingguozao	88.19	88.18	99.98	97.32	92.13	34.36
Beijingjidanzao	88.74	88.72	99.98	97.1	91.55	35.32
Shandonglizao	89.71	89.7	99.98	97.2	90.58	35.51
Shexianmazao	89.85	89.83	99.98	97.07	90.43	34.68
Sunanbaipuzao	90.82	90.81	99.98	96.81	89.44	33.97
Xuyiyanlaihongzao	89.53	89.52	99.98	97.2	90.74	34.41
Wutaimianzao	90.47	90.45	99.98	97.01	89.78	34.74
Jinzandazao	98.1	98.1	99.99	99.33	97.4	34.12
Shandonglajiaozao	98.02	98.02	99.99	99.29	97.28	34.06
Xuputangzao	101.42	101.42	99.99	99.35	97.37	33.88
Shandongyuanling 78005zao	103.78	103.78	99.99	99.24	97.03	34.14
Hengyangzhenzhuzao	102.45	102.45	99.99	99.31	97.27	33.74
Weihaijinsizao3	103.54	103.54	99.99	99.34	97.38	33.98
Tai'angedazao	102.15	102.15	99.99	99.28	97.15	33.93
Xiajinmamazao	107.73	107.73	99.99	99.26	97.01	33.59
Xinzhengdazao	103.61	103.61	99.99	99.29	97.16	34.38
Xinzhengchangjixinzao	103.49	103.49	99.99	99.2	96.85	33.78
Binxianyuanzao	99.78	99.77	99.99	99.22	97.07	34.1
Puchengjinzao	102.66	102.66	99.99	99.35	97.4	34.47
Yucichangmuzao	104.22	104.22	99.99	99.34	97.36	34.43
Jinsizao1	104.28	104.28	99.99	99.18	96.75	34.17
Ruchengzao	105.98	105.98	99.99	99.27	97.06	34.3
Lelingchangmuzao	95.1	95.1	99.99	99.26	97.17	34.14
Puyangsanbianchouzao	100.74	100.74	99.99	99.47	97.55	34.06

Changxindianbaizao	135.34	135.34	99.99	99.47	97.95	35.21
Jinsizao4	100.2	100.2	99.99	99.51	97.72	34.45
Xupuchengtuozao	90.69	90.69	99.99	99.24	97.07	34.37
Tianjindamayazao	102.88	102.88	99.99	99.6	98.13	34.71
Xupuguanyinzao	107.64	107.64	99.99	99.27	97.04	34.5
Tianjinminzao	104.66	104.66	99.99	99.22	96.91	34.02
Xiyingbenzao	102.5	102.5	99.99	99.32	97.32	33.09
Tianjinerqiuzao	99.27	99.27	99.99	99.54	97.84	34.2
Xupujidanzao	104.07	104.07	99.99	99.34	97.37	33.99
Dalizhizao	102.75	102.75	99.99	99.55	97.85	34.59
Lejinzao4	105.72	105.72	99.99	99.36	97.44	34.5
Yucimianzao	102.54	102.54	99.99	99.13	96.55	34.65
Lejinzao2	104.39	104.39	99.99	99.33	97.26	34.29
Lejinzao1	102.57	102.57	99.99	99.27	97.15	34.62
Tai'ansuyuanlingzao	102.56	102.56	99.99	99.32	97.3	33.54
Tengzhoudamazao	102.53	102.53	99.99	99.56	97.92	34.47
Dalilinglingzao	103.68	103.68	99.99	99.32	97.3	34.19
Lingbaodazao	110.36	110.36	99.99	99.54	97.83	34.65
Shaoguanbaizao	101.93	101.91	99.98	99.32	97.01	34.49
Linzedazao	101.43	101.43	99.99	99.3	97.29	34.72
Yunnanzao3	107.7	107.7	99.99	99.32	97.17	33.95
Shenxianchuanganzao	107.4	107.4	99.99	99.22	96.89	33.9
Pingyaokuduanzao	92.01	92.01	99.99	99.32	97.39	33.83
Kongfusucuizao	103.52	103.52	99.99	99.55	97.9	33.78
Taiyuanshiyuehongzao	102.52	102.52	99.99	99.33	97.38	34.25
Ningyangliuyuexianzao	103.68	103.68	99.99	99.38	97.16	34.43
Yuanlingzao1	103.25	103.25	99.99	99.3	97.21	35.08
Dong'edaguazao	99.34	99.34	99.99	99.52	97.75	34.21
Xiajinchahuzao	109.3	109.3	99.99	99.23	96.89	33.98
Xupumizao	104.31	104.31	99.99	99.28	97.11	33.47
Heyanglinglingzao	101.34	101.34	99.99	99.3	97.26	33.69
Xupushatangzao	103.76	103.76	99.99	99.54	97.86	33.61
Jiaxianmidiancuimuzao	96.5	96.5	99.99	99.29	97.29	34.18
Xianxianyuanxiaozao	100.17	100.17	99.99	99.52	97.73	34.29
Sumuzao	103.52	103.52	99.99	99.25	97.03	34.07
Linyimalingzao	102.58	102.58	99.99	99.52	97.73	34.01
Dingxiangxiaozao	101.81	101.81	99.99	99.28	97.15	33.74
Wanrongfuzao	103.26	103.26	99.99	99.25	97.02	33.06
Nanjingzao (Lanxi)	102.44	102.44	99.99	99.13	96.47	33.53
Hebeizao13	99.59	99.59	99.99	99.54	97.87	33.33
Beijingzhuizibaizao	101.78	101.78	99.99	99.32	97.36	34.31
Hanguoyuechuzao	101.07	101.07	99.99	99.31	97.3	33.81
Puchengdundunzao	100.63	100.63	99.99	99.55	97.9	33.79
Pozaozhibianzao1	103.25	103.25	99.99	99.35	97.41	33.8

Xinledazao	102.66	102.66	99.99	99.3	97.23	33.37
Cangxianchangxiaozao	98.96	98.96	99.99	99.28	97.24	32.79
Dalifengmiguangzao	101.09	101.09	99.99	99.55	97.9	33.62
Nangufenghuluzao	105.13	105.13	99.99	99.29	97.2	34.08
Dingxiangshanzao	100.62	100.62	99.99	99.56	97.94	34.69
Yunchengcuizao	101.43	101.43	99.99	99.56	97.93	33.02
Daligedazao	101.97	101.97	99.99	99.34	97.36	33.51
Wenshuishazao	104.33	104.33	99.99	99.28	97.09	33.88
Xianxianmianzao	100.8	100.8	99.99	99.22	96.91	33.65
Shenxianchuanganhongzao	106.26	106.26	99.99	99.21	97	33.69
Wuxiangtianzao	98.74	98.74	99.99	99.52	97.74	32.98
Yanzhousanbianshezao	99.24	99.24	99.99	99.33	97.34	33.74
Taiguhupingzao	98.42	98.42	99.99	99.53	97.77	34.57
Fuyangmutouzao	101.4	101.4	99.99	99.29	97.24	33.43
Miyunxiaozao	105.86	105.86	99.99	99.34	97.39	33.31
Xianxianzao21	106.13	106.13	99.99	99.51	97.72	33.24
Xupuxiaoguosuanpanzao	102.4	102.4	99.99	99.49	97.67	34.04
Jingudazao	106.07	106.07	99.99	99.28	97.17	33.29
Fucuimizao	97.97	97.97	99.99	99.56	97.93	33.58
Xupuchengchuizao	90.79	90.79	99.99	99.27	97.19	33.31
Zanhuangchangzao	101.91	101.91	99.99	99.54	97.85	33.43
Pingyaodazao	103.16	103.16	99.99	99.34	97.43	34.01
Guoxingpingguozhuangzao	90.44	90.44	99.99	99.16	96.72	33.91
Hetaowenzaoss1	103.27	103.27	99.99	99.17	96.71	33.45
Taigumuzao	102.57	102.57	99.99	99.3	97.21	33.1
Hubeijixinzao	108.27	108.27	99.99	99.55	97.89	33.36
Shanxidalingzao	104.25	104.25	99.99	99.16	96.72	32.87
Xupubaopizao	99.45	99.45	99.99	99.25	97.02	32.75
Tengzhoutangzao	93.44	93.44	99.99	99.36	97.52	33.74
Chaoyangwanzao	108.36	108.36	99.99	99.36	97.39	32.93
Weihaijinsizao2	101.29	101.29	99.99	99.26	97.04	33.08
Gansudiaodiaopozao	99.79	99.79	99.99	99.52	97.77	33.97
Xiajindabailingzao	101.67	101.67	99.99	99.28	97.18	33.68
Linyibobozao	105.79	105.79	99.99	99.38	97.55	34.08
Qufuhoutouzao	98.64	98.64	99.99	99.27	97.16	34.6
Taiyuanchangzao	104.3	104.3	99.99	99.23	96.89	34.26
E4nan8zao	103.33	103.33	99.99	99.23	96.9	34.99
Dongzao	102.8	102.8	99.99	99.59	98.04	34.39

Supplementary table 6 Table of Model Fitting Parameters and Residual Results for Variety '510'

Variety name	ModelFit_min(%)	ModelFit_max(%)	Iterations	Tolerance	Residual_SE
Jinzandazao	66.96%	97.67%	8	1.49e-08	1778000
Shandonglajiaozao	66.88%	98.67%	7	1.49e-08	1732000
Xuputangzao	69.11%	98.49%	7	1.49e-08	1789000
Shandongyuanling 78005zao	67.52%	98.37%	7	1.49e-08	1787000

Hengyangzhenzhuzao	68.86%	98.16%	7	1.49e-08	1779000
Weihaijinsizao3	67.91%	98.40%	7	1.49e-08	1747000
Tai'angedazao	68.23%	98.38%	7	1.49e-08	1794000
Xiajinmamazao	68.01%	98.83%	6	1.49e-08	1858000
Xinzhengdazao	65.62%	98.54%	7	1.49e-08	1674000
Xinzhengchangjixinzao	66.59%	98.63%	7	1.49e-08	1815000
Binxianyuanzao	66.07%	98.46%	8	1.49e-08	1748000
Puchengjinzao	65.73%	98.63%	7	1.49e-08	1714000
Yucichangmuzao	66.04%	98.16%	7	1.49e-08	1754000
Jinsizao1	66.91%	98.18%	7	1.49e-08	1771000
Ruchengzao	68.22%	98.43%	6	1.49e-08	1762000
Lelingchangmuzao	66.53%	98.37%	7	1.49e-08	1720000
Puyangsanbianchouzao	67.45%	98.13%	7	1.49e-08	1820000
Changxindianbaizao	68.91%	97.57%	6	1.49e-08	2120000
Jinsizao4	66.39%	98.28%	7	1.49e-08	1788000
Xupuchengtuozao	68.03%	98.18%	8	1.49e-08	1727000
Tianjindamayazao	68.04%	97.84%	7	1.49e-08	1789000
Xupuguanyinzao	67.08%	97.85%	7	1.49e-08	1836000
Tianjinminzao	68.16%	98.07%	7	1.49e-08	1815000
Xiyingbenzao	66.60%	99.04%	7	1.49e-08	1858000
Tianjinerqiuzao	67.35%	98.22%	7	1.49e-08	1724000
Xupujidanzao	67.98%	98.27%	8	1.49e-08	1712000
Dalizhizao	64.89%	98.25%	7	1.49e-08	1738000
Lejinzao4	65.61%	98.35%	7	1.49e-08	1709000
Yucimianzao	66.41%	98.30%	7	1.49e-08	1725000
Lejinzao2	66.23%	98.19%	7	1.49e-08	1775000
Lejinzao1	65.49%	98.50%	8	1.49e-08	1693000
Tai'ansuyuanlingzao	67.50%	98.84%	7	1.49e-08	1833000
Tengzhoudamazao	65.99%	98.50%	7	1.49e-08	1791000
Dalilinglingzao	67.42%	98.09%	7	1.49e-08	1779000
Lingbaodazao	67.59%	98.26%	7	1.49e-08	1792000
Shaoguanbaizao	67.09%	98.56%	10	1.49e-08	2082000
Linzedazao	66.25%	97.93%	7	1.49e-08	1749000
Yunnanzao3	68.46%	98.10%	7	1.49e-08	1917000
Shenxianchuanganzao	68.77%	98.38%	7	1.49e-08	1839000
Pingyaokuduanzao	67.44%	98.39%	7	1.49e-08	1705000
Kongfusucuizao	68.37%	98.13%	7	1.49e-08	1863000
Taiyuanshiyuehongzao	68.95%	98.24%	7	1.49e-08	1783000
Ningyangliuyuexianzao	67.60%	98.03%	6	1.49e-08	1824000
Yuanlingzao1	66.68%	98.06%	7	1.49e-08	1762000
Dong'edaguazao	67.57%	98.15%	7	1.49e-08	1715000
Xiajinchahuzao	68.26%	98.37%	7	1.49e-08	1894000
Xupumizao	69.01%	98.31%	7	1.49e-08	1921000
Heyanglinglingzao	67.39%	98.59%	7	1.49e-08	1836000

Xupushatangzao	68.44%	98.39%	6	1.49e-08	1785000
Jiaxianmidiancuimuzao	66.40%	98.25%	7	1.49e-08	1787000
Xianxianyuanxiaozao	67.19%	98.49%	7	1.49e-08	1760000
Sumuzao	66.57%	98.49%	7	1.49e-08	1669000
Linyimalingzao	67.94%	98.15%	7	1.49e-08	1807000
Dingxiangxiaozao	68.80%	98.41%	7	1.49e-08	1792000
Wanrongfuzao	68.69%	98.96%	8	1.49e-08	1867000
Nanjingzao (Lanxi)	67.35%	99.09%	8	1.49e-08	1790000
Hebeizao13	69.11%	99.10%	7	1.49e-08	1769000
Beijingzhuizibaizao	66.32%	98.19%	7	1.49e-08	1770000
Hanguoyuechuzao	68.56%	98.33%	7	1.49e-08	1820000
Puchengdundunzao	65.90%	98.63%	6	1.49e-08	1802000
Pozaozhibianzao1	66.99%	98.62%	7	1.49e-08	1785000
Xinledazao	67.79%	99.08%	7	1.49e-08	1839000
Cangxianchangxiaozao	68.80%	98.92%	8	1.49e-08	1834000
Dalifengmiguangzao	67.98%	98.47%	7	1.49e-08	1801000
Nangufenghuluzao	67.72%	98.79%	7	1.49e-08	1733000
Dingxiangshanzao	67.12%	97.90%	7	1.49e-08	1714000
Yunchengcuizao	67.66%	99.08%	8	1.49e-08	1787000
Daligedazao	68.93%	99.16%	7	1.49e-08	1738000
Wenshuishazao	67.56%	98.79%	7	1.49e-08	1795000
Xianxianmianzao	67.64%	98.44%	7	1.49e-08	1778000
Shenxianchuanganhongzao	69.33%	98.52%	6	1.49e-08	1808000
Wuxiangtianzao	66.72%	98.86%	8	1.49e-08	1805000
Yanzhousanbianshezao	70.77%	98.98%	13	1.49e-08	1689000
Taiguhupingzao	67.24%	98.44%	7	1.49e-08	1664000
Fuyangmutouzao	68.08%	98.78%	6	1.49e-08	1792000
Miyunxiaozao	69.15%	98.90%	8	1.49e-08	1856000
Xianxianzao21	67.45%	98.96%	7	1.49e-08	1889000
Xupuxiaoguosuanpanzao	68.05%	98.38%	7	1.49e-08	1791000
Jingudazao	68.07%	98.83%	7	1.49e-08	1827000
Fucuimizao	69.18%	97.99%	8	1.49e-08	1698000
Xupuchengchuizao	67.28%	99.04%	10	1.49e-08	1683000
Zanhuangchangzao	68.83%	98.78%	7	1.49e-08	1801000
Pingyaodazao	67.42%	98.49%	7	1.49e-08	1822000
Guoxingpingguozhuangzao	68.04%	98.90%	8	1.49e-08	1616000
Hetaowenzaoss1	68.39%	98.75%	7	1.49e-08	1852000
Taigumuzao	67.39%	98.97%	8	1.49e-08	1862000
Hubeijixinzao	68.77%	98.74%	8	1.49e-08	1957000
Shanxidalingzao	69.33%	98.89%	8	1.49e-08	1877000
Xupubaopizao	69.31%	98.94%	10	1.49e-08	1798000
Tengzhoutangzao	67.83%	98.57%	7	1.49e-08	1716000
Chaoyangwanzao	70.24%	98.89%	8	1.49e-08	1852000
Weihaijinsizao2	70.36%	99.09%	10	1.49e-08	1773000

Gansudiaodiaopozao	67.47%	98.44%	7	1.49e-08	1775000
Xiajindabailingzao	67.39%	98.82%	7	1.49e-08	1719000
Linyibobozao	67.97%	98.63%	7	1.49e-08	1802000
Qufuhoutouzao	66.68%	98.54%	8	1.49e-08	1735000
Taiyuanchangzao	68.20%	98.31%	7	1.49e-08	1789000
E4nan8zao	70.12%	98.57%	9	1.49e-08	1574000
Suanliuliuzao	67.18%	99.02%	6	1.49e-08	1767000
Xianshi	65.16%	98.37%	7	1.49e-08	1855000
Chengwanwucizao3	66.01%	98.80%	7	1.49e-08	1757000
Bianhesuanzao	65.84%	98.48%	7	1.49e-08	1764000
Xingtai0619	65.96%	98.61%	7	1.49e-08	1648000
Xingtai0648	65.31%	98.50%	7	1.49e-08	1734000
Suanzao47	65.61%	98.78%	7	1.49e-08	1745000
Zi4	66.25%	98.86%	7	1.49e-08	1704000
Xingtai0610	66.19%	98.49%	6	1.49e-08	1797000
Suanzao30	65.78%	98.37%	7	1.49e-08	1815000
D12	66.76%	98.34%	6	1.49e-08	1865000
BT34	66.19%	98.10%	6	1.49e-08	1769000
Suanzao45	67.31%	98.14%	7	1.49e-08	1951000
Suanzao43	65.70%	98.37%	7	1.49e-08	1796000
Beiqi11haozao	68.20%	98.78%	7	1.49e-08	1867000
Dongbeisuanzao6	66.81%	98.75%	6	1.49e-08	1812000
Suanzao44	66.45%	98.40%	6	1.49e-08	1765000
LW7	67.45%	98.60%	7	1.49e-08	1834000
LW8	67.80%	98.69%	6	1.49e-08	1848000
LW11	67.26%	98.31%	7	1.49e-08	1821000
LW12	65.72%	98.48%	6	1.49e-08	1714000
LW13	65.91%	98.55%	7	1.49e-08	1769000
LW14	67.47%	98.90%	6	1.49e-08	1702000
Suanzao24	66.85%	98.98%	6	1.49e-08	1844000
LW15	67.37%	98.54%	7	1.49e-08	1755000
LW16	66.69%	98.32%	7	1.49e-08	1741000
LW17	67.34%	98.55%	7	1.49e-08	1769000
LW66	68.16%	98.15%	7	1.49e-08	1788000
Suanzao11	67.50%	98.43%	7	1.49e-08	1881000
Xingtai0648	66.67%	98.76%	7	1.49e-08	1847000
Lanmao1	66.01%	98.67%	6	1.49e-08	1833000
Lanmao2	66.07%	99.08%	6	1.49e-08	1776000
Tesourzao4	65.68%	98.06%	7	1.49e-08	1888000
Yonghesuanzao1	66.97%	98.58%	7	1.49e-08	1816000
Jin1	67.32%	98.47%	7	1.49e-08	1804000
Xingtai0641	67.12%	98.34%	7	1.49e-08	1796000
Xingtai0608	66.03%	98.29%	6	1.49e-08	1837000
Zi1	65.55%	98.31%	6	1.49e-08	1928000

Chengde5	67.44%	98.76%	7	1.49e-08	1871000
Xingtai0613	66.12%	98.39%	7	1.49e-08	1806000
Suanzaowang 3	66.58%	98.71%	7	1.49e-08	1734000
C22	66.53%	98.52%	6	1.49e-08	1691000
Zi3	67.98%	98.71%	6	1.49e-08	1832000
T37	67.68%	98.68%	6	1.49e-08	1800000
Dongbeisuanzao3	67.20%	98.52%	7	1.49e-08	1710000
C16	67.79%	98.80%	6	1.49e-08	1844000
Yonghesuanzao3	67.08%	98.99%	6	1.49e-08	1868000
Suanzao48	65.83%	98.56%	6	1.49e-08	1834000
Xingtai0609	67.32%	98.37%	7	1.49e-08	1789000
Dongbeisuanzao5	65.75%	98.31%	7	1.49e-08	1734000
Xingtai16	66.88%	98.76%	7	1.49e-08	1748000
Suanzao20	66.37%	98.49%	7	1.49e-08	1775000
Suanzao23	67.03%	98.71%	6	1.49e-08	1797000
Suanzao3	66.07%	99.03%	6	1.49e-08	1765000
ShandonggaoweishengsuC	67.85%	99.02%	6	1.49e-08	1771000
Shanxixiaosuanzao	67.10%	98.45%	6	1.49e-08	1829000
C3	66.74%	98.33%	7	1.49e-08	1816000
Suanzao1	67.73%	98.54%	6	1.49e-08	1850000
Dongbeisuanzao4	66.64%	98.43%	6	1.49e-08	1795000
Shandongsuanzao3	66.98%	98.51%	7	1.49e-08	1867000
C6	67.52%	98.51%	7	1.49e-08	1830000
Suanzao71	66.76%	98.83%	6	1.49e-08	1870000
Suanzao17	66.01%	99.03%	6	1.49e-08	1794000
Suanzao14	65.04%	98.58%	7	1.49e-08	1756000
Yonghesuanzao5	65.61%	98.47%	7	1.49e-08	1800000
Suanzao8	66.17%	98.49%	7	1.49e-08	1860000
Dongbeisuanzao	67.38%	98.40%	7	1.49e-08	1731000
Suanzao46	68.05%	98.46%	6	1.49e-08	2013000
Suanzao40	66.64%	98.77%	6	1.49e-08	1795000
Shandongsuanzao2	67.60%	98.91%	6	1.49e-08	1897000
Xingtai0614	68.90%	98.91%	6	1.49e-08	1834000
Suanzao42	67.85%	98.95%	6	1.49e-08	1867000
Henansuanzao1	67.41%	98.46%	6	1.49e-08	1814000
Suanzao15	67.78%	98.26%	6	1.49e-08	1819000
Shandongsuanzao5	66.85%	98.49%	6	1.49e-08	1818000
Liaoning Chaoyang2	66.56%	98.66%	7	1.49e-08	1793000
Suanzao32	67.13%	98.77%	6	1.49e-08	1825000
Suanzao4	67.55%	98.88%	6	1.49e-08	1831000
Dongbeisuanzao7	67.70%	98.86%	6	1.49e-08	1836000
Yonghesuanzao4	66.14%	98.55%	6	1.49e-08	1762000
Beiketi2	67.93%	99.03%	6	1.49e-08	1749000
Yonghesuanzao1	67.33%	98.71%	7	1.49e-08	1739000

Suanzao41	64.74%	98.61%	6	1.49e-08	1671000
Beiketi1	65.46%	98.62%	6	1.49e-08	1725000
Xingtai10	66.72%	98.77%	6	1.49e-08	1799000
D6	66.35%	98.79%	6	1.49e-08	1886000
GaoweishengsuCsuanzao	66.68%	98.84%	6	1.49e-08	1722000
Wenwansuanzao	67.69%	99.08%	6	1.49e-08	1831000
DY39	66.17%	98.90%	7	1.49e-08	1835000
DY40	64.66%	98.74%	7	1.49e-08	1813000
DY41	66.07%	98.54%	7	1.49e-08	1704000
DY42	65.91%	98.49%	6	1.49e-08	1838000
DY43	67.22%	98.69%	7	1.49e-08	1936000
DY44	67.47%	98.85%	6	1.49e-08	1835000
DY45	66.62%	98.92%	6	1.49e-08	1863000
DY46	66.73%	98.77%	7	1.49e-08	1807000
DY48	68.55%	98.34%	6	1.49e-08	2009000
DY49	67.11%	98.34%	7	1.49e-08	1759000
DY50	67.57%	98.49%	7	1.49e-08	1694000
DY54	66.93%	97.83%	6	1.49e-08	1960000
DY31	66.38%	98.26%	6	1.49e-08	1845000
DY30	68.47%	98.52%	6	1.49e-08	1848000
DY29	67.72%	98.08%	6	1.49e-08	1823000
DY28	65.81%	98.44%	7	1.49e-08	1802000
DY26	67.95%	98.07%	6	1.49e-08	1913000
DY25	64.77%	98.28%	8	1.49e-08	1822000
DY24	67.59%	98.08%	7	1.49e-08	1828000
DY23	66.98%	98.74%	7	1.49e-08	1833000
DY22	67.80%	98.54%	7	1.49e-08	1888000
DY21	67.82%	98.96%	6	1.49e-08	1804000
DY20	67.50%	98.58%	6	1.49e-08	1808000
DY19	67.38%	98.42%	7	1.49e-08	1808000
DY18	67.92%	98.37%	6	1.49e-08	1855000
DY17	67.40%	98.48%	6	1.49e-08	1809000
DY16	69.01%	96.06%	6	1.49e-08	2792000
DY15	66.86%	98.72%	7	1.49e-08	1724000
DY14	66.94%	98.70%	6	1.49e-08	1893000
DY13	67.83%	99.09%	6	1.49e-08	1674000
DY12	65.37%	98.27%	7	1.49e-08	1769000
DY11	67.61%	98.64%	7	1.49e-08	1773000
DY9	67.20%	98.57%	6	1.49e-08	1843000
DY8	66.56%	98.25%	7	1.49e-08	1840000
DY55	67.54%	98.26%	7	1.49e-08	1751000
DY1	67.16%	98.77%	6	1.49e-08	1874000
DY2	67.37%	98.51%	6	1.49e-08	1809000
DY3	67.57%	98.52%	6	1.49e-08	1806000

DY4	66.93%	98.30%	6	1.49e-08	1788000
DY5	67.28%	98.55%	6	1.49e-08	1766000
DY6	67.13%	98.57%	6	1.49e-08	1789000
Jingxin2	67.06%	98.34%	6	1.49e-08	1820000
Taizijing	66.41%	98.68%	7	1.49e-08	1811000
Zaoyan3	66.04%	98.94%	6	1.49e-08	1876000
Dahuzi	67.76%	98.52%	6	1.49e-08	1842000
Suanzao32	67.52%	98.43%	7	1.49e-08	1773000
Shanxibianzao	68.22%	98.68%	7	1.49e-08	1758000
Fupingcentenariansuanzao	69.45%	98.70%	6	1.49e-08	1835000
Shanxiyuanzao	67.53%	98.60%	7	1.49e-08	1810000
Cangzhou 1	67.01%	98.29%	6	1.49e-08	1824000
DY55	66.96%	98.91%	7	1.49e-08	1821000
DY56	67.15%	99.04%	6	1.49e-08	1799000
DY57	67.29%	98.77%	6	1.49e-08	1948000
Xingtai10	66.18%	98.06%	7	1.49e-08	1818000
Zhongdaguotefeng1	67.70%	98.50%	7	1.49e-08	1771000
Zhongchanfengguoshaoci2	67.01%	98.36%	6	1.49e-08	1826000
Daguofengchan3	66.63%	98.49%	6	1.49e-08	1821000
Zhongdaguofengshaocikouganhao5	68.38%	98.51%	6	1.49e-08	1814000
Suanzao73	67.12%	98.66%	6	1.49e-08	1886000
D1Y2	67.88%	98.91%	6	1.49e-08	1962000
Suanzao69	66.74%	98.47%	7	1.49e-08	1962000
No.194	66.69%	98.90%	6	1.49e-08	1810000
Huzhuanglaozaoshu	66.37%	98.43%	7	1.49e-08	1851000
Xingzhou2	66.47%	98.50%	7	1.49e-08	1808000
Xingzhou9	66.48%	98.55%	7	1.49e-08	1782000
Wanglaofengchenzao	68.08%	98.42%	7	1.49e-08	1798000
Taihang1	66.74%	98.30%	7	1.49e-08	1821000
Tianshi	68.32%	99.05%	7	1.49e-08	1927000
Sandaiyizao	67.82%	98.97%	6	1.49e-08	1860000
Sandaisizao	66.85%	98.67%	6	1.49e-08	1858000
Pingyinzimengsuanzao	67.20%	98.47%	6	1.49e-08	1801000
Xingzhou16	66.94%	98.36%	7	1.49e-08	1752000
D1Y3	64.78%	98.07%	8	1.49e-08	1783000
Shanxichangyuanzao	67.49%	98.51%	6	1.49e-08	1793000
Gaotaisuanzao	67.30%	98.79%	6	1.49e-08	1856000
Suanzao36	67.46%	98.43%	7	1.49e-08	1913000
F15	68.34%	98.28%	6	1.49e-08	2073000
DY53	65.53%	98.58%	7	1.49e-08	1796000
DY32	66.91%	98.26%	7	1.49e-08	1805000
DY33	67.57%	98.48%	7	1.49e-08	1744000
DY34	66.88%	98.33%	7	1.49e-08	1716000
DY35	67.18%	98.72%	7	1.49e-08	1771000

DY36	65.32%	98.34%	10	1.49e-08	1880000
DY37	66.94%	98.46%	6	1.49e-08	1887000
DY38	67.56%	98.34%	6	1.49e-08	2024000
DY7	66.11%	98.35%	9	1.49e-08	1874000
N2-1	66.11%	99.11%	6	1.49e-08	1766000
N1-72	67.53%	98.62%	6	1.49e-08	1801000
N1-67	68.29%	98.73%	7	1.49e-08	1717000
N1-66	66.79%	98.89%	6	1.49e-08	1881000
N2-17	66.93%	99.01%	6	1.49e-08	1848000
N2-18	66.86%	98.67%	6	1.49e-08	1834000
N1-54	67.62%	98.69%	7	1.49e-08	1808000
N1-34	68.11%	98.89%	6	1.49e-08	1854000
N1-25	68.05%	99.03%	6	1.49e-08	1873000
N2-55	68.29%	98.86%	6	1.49e-08	1818000
N1-10	67.59%	98.63%	7	1.49e-08	1782000
N1-7	68.84%	98.90%	6	1.49e-08	1882000
LW1	65.72%	98.91%	6	1.49e-08	1810000
LW2	67.65%	98.76%	6	1.49e-08	1812000
LW3	65.02%	98.72%	7	1.49e-08	1732000
LW4	67.11%	97.99%	6	1.49e-08	1826000
LW5	67.48%	98.14%	6	1.49e-08	1799000
Madu8haowuci-1	66.40%	98.40%	7	1.49e-08	1711000
Madu8haowuci-2	65.51%	98.52%	7	1.49e-08	1703000
Madu8haowuci-3	65.80%	98.52%	7	1.49e-08	1733000
Madu8haowuci-4	67.39%	98.80%	6	1.49e-08	1762000
Madu8haowuci-5	67.46%	98.79%	6	1.49e-08	1870000
Xibeiduanzhi109	54.47%	88.73%	10	1.49e-08	1853000
Xibeiduanzhi152	64.64%	98.67%	6	1.49e-08	1950000
Xibeiduanzhi171	58.55%	89.10%	16	1.49e-08	1590000
LW7-18	68.15%	98.17%	7	1.49e-08	1524000
LW7	55.21%	87.02%	13	1.49e-08	2476000
LW7-19	68.58%	98.80%	14	1.49e-08	1644000
Xibeiduanzhi190	68.80%	98.59%	6	1.49e-08	1744000
Xibeiduanzhi193	67.94%	98.91%	7	1.49e-08	1713000
Xibeiduanzhi194	54.25%	87.58%	16	1.49e-08	2415000
Beiketi2-2	66.86%	98.66%	6	1.49e-08	1816000
Madu18	63.34%	98.29%	7	1.49e-08	1934000
Xibeiduanzhi199	53.10%	88.36%	12	1.49e-08	1854000
LW7-20	60.96%	97.92%	6	1.49e-08	2430000
LW14zidai55	54.41%	89.13%	15	1.49e-08	1956000
Xibeiduanzhi67	64.36%	97.23%	15	1.49e-08	2188000
Xibeiduanzhi87	61.35%	97.93%	7	1.49e-08	2447000
Xibeiduanzhi90	62.19%	98.31%	6	1.49e-08	2237000
Xibeiduanzhi91	64.58%	98.56%	6	1.49e-08	1807000

Jishanbanzao	63.68%	97.80%	17	1.49e-08	1665000
Linyilizao	62.98%	96.89%	5	1.49e-08	1733000
Yucituanzao	64.49%	96.57%	7	1.49e-08	1779000
Taiguduanzizao	65.48%	97.71%	6	1.49e-08	1827000
Taiguhuluzao	65.23%	97.06%	7	1.49e-08	1800000
Pingyaobuluosuzao	65.07%	96.79%	7	1.49e-08	1807000
Pinglujianzao	64.16%	96.68%	7	1.49e-08	1806000
Linfenmizao	65.56%	96.56%	6	1.49e-08	1876000
Linfenzhenhuluzao	66.30%	97.36%	5	1.49e-08	1862000
Yongjihamazao	65.22%	97.66%	5	1.49e-08	1952000
Jiaochengjunzao	65.48%	97.50%	6	1.49e-08	1778000
Taiguheiyezao	65.05%	97.49%	6	1.49e-08	1778000
Yunchengpopozao	65.42%	96.29%	7	1.49e-08	1816000
Pinglutuntunzao	65.00%	96.78%	7	1.49e-08	1832000
Beijingpaopaozao	65.11%	96.89%	7	1.49e-08	1869000
Baodeyouzao	64.83%	97.03%	7	1.49e-08	1905000
Taigulangzao	66.54%	96.74%	6	1.49e-08	1894000
Xiangzao	65.11%	96.88%	7	1.49e-08	1809000
Hongzhaocuizao	64.81%	97.98%	7	1.49e-08	1864000
Lichengxiaozao	65.15%	96.70%	5	1.49e-08	1879000
Taigudundunzao	65.39%	97.07%	6	1.49e-08	1837000
Xiangfenguantanzao	64.54%	96.92%	5	1.49e-08	1874000
Taigumeimizao	65.48%	97.76%	6	1.49e-08	1862000
Beijingbenzao	64.67%	96.65%	7	1.49e-08	1851000
Xiangfenyuanzao	65.04%	98.15%	5	1.49e-08	1915000
Hongzhaohuluzao	63.76%	96.76%	7	1.49e-08	1842000
Baodexiaozao	64.24%	96.82%	5	1.49e-08	1903000
Jishanyuanzao	63.98%	96.90%	5	1.49e-08	1755000
Hongzhaoshiyuehongzao	65.10%	96.97%	7	1.49e-08	1845000
Zhongyangmuzao	65.41%	98.08%	6	1.49e-08	1933000
Pingshunjunzao	64.12%	96.56%	7	1.49e-08	1845000
Dingxiangxingxingzao	64.42%	97.00%	6	1.49e-08	1850000
Taigulinglingzao	64.10%	96.84%	8	1.49e-08	1718000
Xiangfenmuzao	66.15%	97.97%	6	1.49e-08	1813000
Jishanliuguanzao	65.22%	97.21%	7	1.49e-08	1786000
Zhongyangtuanzao	65.96%	96.00%	6	1.49e-08	1838000
Xiangfenyazao	65.18%	97.10%	6	1.49e-08	1922000
Yuciyazao	63.72%	97.24%	7	1.49e-08	1778000
Jiaochengtiansuanzao	65.78%	97.78%	6	1.49e-08	1866000
Yuanquzao	64.10%	96.85%	5	1.49e-08	1798000
Hongzhaoxiaozao	65.74%	97.45%	5	1.49e-08	1817000
Xiaxianyuancuizao	64.71%	96.88%	6	1.49e-08	1819000
Pingshunbenzao	64.00%	96.40%	7	1.49e-08	1892000
Jiaochengduanzao	64.88%	96.99%	7	1.49e-08	1794000

Xiaxianziyuanzao	65.45%	96.79%	6	1.49e-08	1813000
Linfentuanzao	65.61%	96.76%	7	1.49e-08	1803000
Shanxiqiyuexianzao	64.86%	96.70%	6	1.49e-08	1850000
Zaoqiangpozao	63.66%	98.08%	6	1.49e-08	1827000
Qingyuandadanzao	64.53%	96.67%	7	1.49e-08	1821000
Taigushenglizao	66.25%	96.14%	6	1.49e-08	1901000
Xinzhengdamayazao	62.73%	96.52%	6	1.49e-08	1857000
Dalixiaodundunzao	66.12%	96.21%	7	1.49e-08	1799000
Binxianshuizao	63.49%	96.58%	6	1.49e-08	1810000
Daliyuanzao	64.05%	96.87%	7	1.49e-08	1881000
Yanchuanniunaicuizao	64.68%	97.14%	5	1.49e-08	1903000
Zaoqianggutouxiaozao	65.99%	96.30%	5	1.49e-08	1867000
Gusuxiaozao	64.93%	97.69%	6	1.49e-08	1853000
Xinzhengjidanzao	63.84%	96.73%	5	1.49e-08	1832000
Huanghuadongzao	65.47%	97.21%	5	1.49e-08	1834000
Dalimayazao	64.59%	96.88%	7	1.49e-08	1825000
Puchengzhishezao	65.13%	97.09%	7	1.49e-08	1769000
Yanchuandabaizao	64.39%	97.51%	7	1.49e-08	1770000
Xianxianlajiaozao	63.55%	96.47%	6	1.49e-08	1810000
Yongchengyuanhongzao	64.53%	96.37%	7	1.49e-08	1905000
Lintongguluzao	63.68%	96.60%	6	1.49e-08	1859000
Cangxiantunzizao	64.45%	97.12%	7	1.49e-08	1845000
Xianxianxiaoxiaozao	66.74%	96.37%	7	1.49e-08	1891000
Neihuangbianhesuanzao	64.97%	97.68%	6	1.49e-08	1913000
Xincaidayuanfengzao	63.29%	96.35%	8	1.49e-08	1754000
Linxianwutouzao	64.41%	96.96%	7	1.49e-08	1915000
Dalijidanzao	63.18%	96.50%	6	1.49e-08	1767000
Binxianheigadazao	64.75%	96.37%	7	1.49e-08	1848000
Daliganweibazao	63.40%	96.12%	6	1.49e-08	1864000
Xianxianmuzao	64.37%	96.49%	7	1.49e-08	1847000
Zhenpingtailihongzao	64.39%	96.26%	7	1.49e-08	1875000
Yongchengchanghongzao	63.75%	96.37%	8	1.49e-08	1838000
Yanchuanbaizao	65.25%	96.98%	6	1.49e-08	1800000
Zaoqiangcuizao	62.75%	97.74%	6	1.49e-08	1800000
Xinzhengqitoubaizao	66.91%	97.24%	5	1.49e-08	1898000
Zaoqiangmalianxiaozao	67.62%	96.23%	5	1.49e-08	1952000
Cangxianxiaozao	64.59%	96.73%	7	1.49e-08	1808000
Taigumeixinhongzao	65.05%	97.68%	6	1.49e-08	1811000
Taiguguoyanhongzao	63.48%	96.16%	8	1.49e-08	1855000
Yanchuandieyazao	62.35%	97.51%	22	1.49e-08	1710000
Shanxinaizao	63.45%	96.14%	8	1.49e-08	1777000
Tengzhouchanghongzao	64.70%	96.11%	6	1.49e-08	1888000
Shulutangzao	63.62%	96.02%	5	1.49e-08	1848000
Cangxianjinsixiaozao	64.32%	95.73%	6	1.49e-08	1824000

Xinzhenghuizao	64.41%	96.64%	6	1.49e-08	1845000
Xinzhengxiaoyuanzao	64.97%	97.70%	7	1.49e-08	1858000
Yanchuantiaozao	64.84%	96.44%	6	1.49e-08	1901000
Puchengyuanlizao	66.83%	96.18%	5	1.49e-08	1879000
Binxiansuangedazao	65.64%	96.03%	6	1.49e-08	1857000
Shanximianzao	64.31%	96.36%	6	1.49e-08	1817000
Lelingwuhexiaozao	65.90%	97.58%	6	1.49e-08	1900000
Lelingxiaozao	63.25%	95.73%	7	1.49e-08	1794000
Dayewuhezao	64.43%	96.44%	6	1.49e-08	1937000
Dalipachizao	63.70%	96.25%	6	1.49e-08	1846000
Zhongcaobenzao	64.00%	96.39%	6	1.49e-08	1888000
Yutianxiaozao	66.63%	95.80%	6	1.49e-08	1902000
Puyangsanbianhongzao	64.94%	96.41%	5	1.49e-08	1897000
Hebeilongzao	64.94%	96.24%	6	1.49e-08	1884000
Hanguowudengzao	65.31%	96.73%	6	1.49e-08	1838000
Anyangtuanzao	65.15%	97.10%	7	1.49e-08	1873000
Dalilongzao	65.07%	96.73%	6	1.49e-08	1824000
Binxianjinzao	64.65%	96.68%	6	1.49e-08	1807000
Liaochengyuanlingzao	68.05%	96.45%	5	1.49e-08	1892000
Puyangtangzao	63.86%	96.17%	6	1.49e-08	1872000
Zhenpingguangyangzao	64.38%	96.23%	5	1.49e-08	1872000
Zaozhuanggongzao	64.94%	97.14%	7	1.49e-08	1792000
Hamidazao	64.38%	96.73%	7	1.49e-08	1870000
Zaoqiangshazao	63.39%	96.59%	6	1.49e-08	1811000
Xuechengdongzao	64.84%	96.34%	7	1.49e-08	1827000
Xinzhengchangjixinzao	65.73%	97.79%	7	1.49e-08	1887000
Yangjiaozao	65.14%	96.30%	7	1.49e-08	1826000
Lelingmopanzao	64.62%	95.89%	6	1.49e-08	1884000
Ningyangxuanlingzao	63.90%	96.47%	6	1.49e-08	1863000
Tai'andacuizao	63.61%	96.22%	8	1.49e-08	1887000
Chengwudongzao	63.55%	96.49%	8	1.49e-08	1768000
Tengzhoudamayazao	63.42%	96.86%	8	1.49e-08	1802000
Zhenpingjiuyuehanzao	63.45%	95.79%	6	1.49e-08	1844000
Lengbaiyuzao	64.59%	95.92%	7	1.49e-08	1847000
Xi'anyangnaizao	63.84%	96.10%	8	1.49e-08	1826000
Jiaxianyazao	64.34%	96.54%	6	1.49e-08	1930000
Yanchuangoutouzao	64.78%	96.62%	8	1.49e-08	1769000
Shanxihuluzao	64.15%	96.62%	6	1.49e-08	1867000
Dalixiaoyuanzao	64.26%	96.57%	6	1.49e-08	1763000
Xinzhengjiuyueqingzao	64.76%	97.70%	7	1.49e-08	1918000
Puyangxiaozao	65.28%	96.48%	6	1.49e-08	1837000
Puyanghetaowenzao	64.26%	96.36%	5	1.49e-08	1904000
Songxiandazao	63.10%	96.22%	5	1.49e-08	1788000
Tai'anmalingcuizao	63.66%	96.54%	7	1.49e-08	1872000

Ningyangdashibingzao	63.92%	95.40%	8	1.49e-08	1913000
Beibeixiaozao	65.56%	97.74%	6	1.49e-08	1904000
Tengzhouluodihongzao	63.39%	97.32%	5	1.49e-08	1827000
Fengjiejidanzao	64.33%	96.71%	8	1.49e-08	1779000
Qingyunxiaolizao	64.46%	96.51%	6	1.49e-08	1875000
Beijingmayazao	64.11%	96.79%	8	1.49e-08	1868000
Beijingyingluozao	64.63%	97.55%	7	1.49e-08	1898000
Tianjingagazao	62.17%	96.71%	8	1.49e-08	1810000
Tianjinkuaizao	64.91%	96.29%	7	1.49e-08	1869000
Beijinglangjiayuanzao	62.40%	95.46%	6	1.49e-08	1910000
Qiyangkangtouzao	64.13%	96.24%	7	1.49e-08	1856000
Xupudaguosuanpanzao	63.38%	96.49%	8	1.49e-08	1778000
Hengshanchangdazao	64.88%	96.22%	7	1.49e-08	1881000
Xuputiansuanzao	67.01%	96.63%	8	1.49e-08	1804000
Xupuhuluzao	66.47%	95.91%	6	1.49e-08	1893000
Hunanchangzao	65.93%	96.55%	7	1.49e-08	1895000
Xupubinglangzao	64.77%	95.56%	6	1.49e-08	1837000
Xupumuzao	63.88%	96.36%	8	1.49e-08	1818000
Xupuyuanzao	64.73%	97.46%	7	1.49e-08	1796000
Lanximazao	63.49%	96.15%	8	1.49e-08	1847000
Shengxianbaipuzao	67.38%	96.73%	5	1.49e-08	1899000
Xuanchengjianzao	65.08%	95.90%	6	1.49e-08	1870000
Nanjingdamuzao	66.15%	96.39%	5	1.49e-08	1830000
Langxiniunaizao	65.56%	95.88%	8	1.49e-08	1903000
Yiwuezizao	64.09%	95.75%	6	1.49e-08	1901000
Fuyangmayizao	67.28%	95.96%	6	1.49e-08	1880000
Yiwudazao	66.84%	96.57%	6	1.49e-08	1925000
Hubeilingdangzao	64.25%	96.55%	6	1.49e-08	1862000
Gansudongzao	64.61%	97.31%	8	1.49e-08	1820000
Hubeiyuanzao	64.18%	96.39%	6	1.49e-08	1854000
Zhongningdiaolingzao	64.18%	96.25%	8	1.49e-08	1840000
Zhongningxiaoyuanzao	63.93%	96.43%	6	1.49e-08	1845000
Guanyangchangzao	63.28%	96.13%	5	1.49e-08	1770000
Nanjingyazao	64.04%	96.49%	6	1.49e-08	1842000
Guanyangduanzao	65.06%	97.49%	5	1.49e-08	1858000
Wuxianshuituanzao	65.83%	96.10%	6	1.49e-08	1900000
Nanjinglengzao	64.83%	97.64%	7	1.49e-08	1894000
kashengaerxiaozao	63.19%	96.78%	5	1.49e-08	1816000
Linzexiaozao	64.46%	96.51%	6	1.49e-08	1849000
Dunhuangdazao	62.75%	95.70%	8	1.49e-08	1845000
Chaoyangdajiandingzao	62.63%	96.25%	8	1.49e-08	1829000
Kuerlexiaozao	64.87%	97.26%	6	1.49e-08	1739000
Xinjiangchangyuanzao	63.99%	95.99%	8	1.49e-08	1809000
Chaoyangdapingdingzao	67.64%	96.89%	5	1.49e-08	1951000

Anningxiaozao	65.83%	96.62%	6	1.49e-08	1894000
Minqinxiaozao	64.14%	96.43%	6	1.49e-08	1851000
Aksuxiaozao	65.66%	97.46%	7	1.49e-08	1835000
Zunyitianzao	63.45%	96.29%	8	1.49e-08	1825000
Lianxiantangzao	63.94%	96.47%	8	1.49e-08	1824000
Lianxianmuzao	64.41%	96.52%	5	1.49e-08	1873000
Yixianmuzao	63.28%	96.09%	8	1.49e-08	1827000
Shaoguanbaizao	64.46%	96.17%	7	1.49e-08	1920000
Xishuangbannaxiaozao	64.57%	96.32%	6	1.49e-08	1806000
Gendedazao	65.97%	96.63%	6	1.49e-08	1866000
Lianxiankulianzao	65.38%	96.24%	6	1.49e-08	1905000
Guangdongzhenzhuzao	63.90%	95.70%	8	1.49e-08	1860000
Kunmingzao	65.39%	96.47%	6	1.49e-08	1917000
Huluchanghongzao	64.67%	97.35%	7	1.49e-08	1864000
Neihuangpingguozao	64.13%	96.83%	8	1.49e-08	1860000
Beijingjidanzao	65.84%	95.92%	6	1.49e-08	1905000
Shandonglizao	66.15%	96.67%	8	1.49e-08	1875000
Shexianmazao	64.96%	96.77%	7	1.49e-08	1883000
Sunanbaipuzao	69.62%	97.41%	5	1.49e-08	1938000
Xuyiyanlaihongzao	68.95%	97.21%	6	1.49e-08	1966000
Wutaimianzao	65.98%	96.77%	6	1.49e-08	1826000
Dongzao	67.58%	97.47%	8	1.49e-08	1863000
